# ChatGPT (GPT-4) passed the Japanese National License Examination for Pharmacists in 2022, answering all items including those with diagrams: a descriptive study

**DOI:** 10.3352/jeehp.2024.21.4

**Published:** 2024-02-28

**Authors:** Hiroyasu Sato, Katsuhiko Ogasawara

**Affiliations:** 1 Department of Pharmacy, Abashiri Kosei General Hospital, Abashiri, Japan; 2 Graduate School of Health Sciences, Hokkaido University, Sapporo, Japan; 3 Graduate School of Engineering, Muroran Institute of Technology, Muroran, Japan; Hallym University, Korea

**Keywords:** Artificial intelligence, Japan, Pharmacists, Pharmacy licensure, Task performance

## Abstract

**Purpose:**

The objective of this study was to assess the performance of ChatGPT (GPT-4) on all items, including those with diagrams, in the Japanese National License Examination for Pharmacists (JNLEP) and compare it with the previous GPT-3.5 model’s performance.

**Methods:**

The 107th JNLEP, conducted in 2022, with 344 items input into the GPT-4 model, was targeted for this study. Separately, 284 items, excluding those with diagrams, were entered into the GPT-3.5 model. The answers were categorized and analyzed to determine accuracy rates based on categories, subjects, and presence or absence of diagrams. The accuracy rates were compared to the main passing criteria (overall accuracy rate ≥62.9%).

**Results:**

The overall accuracy rate for all items in the 107th JNLEP in GPT-4 was 72.5%, successfully meeting all the passing criteria. For the set of items without diagrams, the accuracy rate was 80.0%, which was significantly higher than that of the GPT-3.5 model (43.5%). The GPT-4 model demonstrated an accuracy rate of 36.1% for items that included diagrams.

**Conclusion:**

Advancements that allow GPT-4 to process images have made it possible for LLMs to answer all items in medical-related license examinations. This study’s findings confirm that ChatGPT (GPT-4) possesses sufficient knowledge to meet the passing criteria.

## Graphical abstract

[Fig f3-jeehp-21-04]

## Introduction

### Background/rationale

A formidable exemplar among large language models (LLM), the Chat Generative Pre-training Transformer (ChatGPT) harbors immense promise for the field of healthcare. ChatGPT, an advanced example of an LLM, utilizes a transformer model trained on a substantial volume of data encompassing numerous parameters. This chatbot-style LLM is notable for its ability to provide highly accurate answers in natural language and is freely accessible for public use. GPT-3.5, the first model released in November 2022, has 175 billion parameters and is available for free to all users. GPT-4, which was released in March 2023, is even more accurate, with over a trillion parameters, and is only available to paid users. ChatGPT (GPT-4) has continued to evolve, with an update in September 2023 introducing image recognition capabilities and another in October 2023 releasing image generation features.

Numerous studies have evaluated ChatGPT’s performance in answering items related to medical knowledge, including several concerning licensing exams [1-9]. ChatGPT mainly comprises GPT-3.5 and GPT-4 as generation models, with the more recent GPT-4 model having higher accuracy. Early studies evaluating the performance of ChatGPT for medical licensing exams were based on the GPT-3.5 model; thus, many failed to meet the passing criteria [1-3]. Subsequently, numerous studies surpassed the acceptance criteria for performance evaluations using the GPT-4 model [6-9].

However, because both ChatGPT iterations (GPT-3.5 and the original version of GPT-4) are incapable of interpreting images, existing studies omitted items that include diagrams, tables, and complex mathematical formulae. However, an update for GPT-4 was released on September 25, 2023, with image handling capabilities, enabling a comprehensive evaluation of the effectiveness of LLMs across all items covered in the medical certification exams.

### Objectives

The purpose of this study is to evaluate the performance of ChatGPT (GPT-4) for all items on the 107th Japanese National License Examination for Pharmacists (JNLEP), conducted in 2022. Furthermore, after excluding the image items, we compared the performance of GPT-3.5 and GPT-4 on the same items from the 107th JNLEP and evaluated the differences in accuracy.

## Methods

### Ethics statement

Ethical approval was not necessary as the study did not use any personal data and no human subjects were involved.

### Study design

This is a descriptive study comparing the performance of GPT-3.5 and GPT-4 to solve the licensing examination.

### Setting

The JNLEP is conducted once a year and can be taken by those who have completed (or are expected to complete) a 6-year pharmacy education program at a university. The latest criteria [10], formulated in 2016 and adapted from the 106th JNLEP conducted in 2021, stipulate the following:

The items evaluate the basic knowledge, skills, and attitudes that pharmacists must have. Consideration will be given to confirming high ethical standards, education as a medical professional, and practical skills that can be used in the medical field. Also considered are items using images, photographs, diagrams, and tables [10].

In this study, the 107th JNLEP conducted in February 2022 was used. The 107th JNLEP consists of 345 items covering the following 9 subjects: physics, chemistry, biology, hygiene, pharmacology, pharmaceuticals, pathophysiology, regulations, and practice. Items are divided into 2 categories (90 essential and 255 general items). All the items were asked in a multiple-choice format. Essential items are formatted such that 1 answer is selected from 5 options, whereas general items are formatted such that 1 or 2 answers are selected from 5 options. For items requiring the selection of 2 answers, both answers must be correct for the response to be scored.

The passing criteria for the 107th JNLEP are the following 3 conditions: (1) a score of 434 points or higher on all items (each question is worth 2 points, equating to a minimum overall accuracy rate of 62.9% or higher); (2) for all essential items, the score must be 70% or more and for each subject of the essential items, 30% or more (the subjects of physics, chemistry, and biology are treated as 1 group); and (3) no more than 2 contraindicated choices. As it has not been announced which options are contraindicated, parameter C was not considered in this study.

In the published correct answers, 1 question was defined as having “no solution (any option choice considered correct),” therefore, it was not entered into ChatGPT, but a score was still assigned.

### Variables

Outcome variables were the accuracy rates of GPT-3.5 and GPT-4’s answers to the JNLEP in 2021 according to examination subject.

### Data sources and measurement

The items and answers to the 107th JNLEP are available as open-source material at https://www.mhlw.go.jp/stf/seisakunitsuite/bunya/0000198924.html in Japanese (Supplements 1–3).

**Fig. 1.**
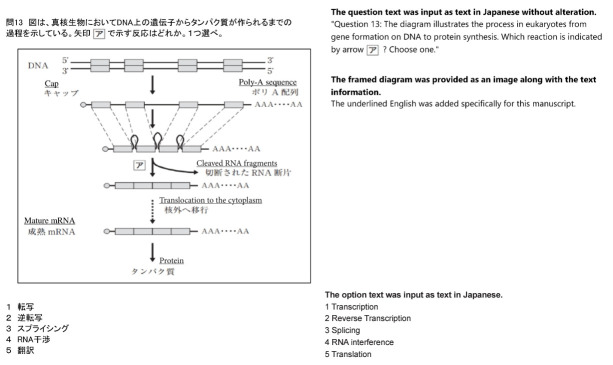
Example of entering items with diagrams into GPT-4.

A total of 284 items, excluding those with diagrams and complex equations that were difficult to input, were entered into GPT-3.5 in their original Japanese text between February 13 and 26, 2023. Simple tables were input using delimiters such as colons or hyphens. When the options were numerical, option numbers were changed from digits to letters. For GPT-4, the full set of 344 items was used, including those with diagrams and complex formulas in their original Japanese text, between November 15 and 19, 2023. The input text for the GPT-4 model was the same as that for the GPT-3.5 model. For items that included diagrams, only the diagrams were input as images (Fig. 1).

The answers from ChatGPT (GPT-3.5 and GPT-4) were scored by cross-referencing them with published correct answers. The performance of each model was evaluated based on the total score, category score, subject score, and comparison with the passing criteria. Additionally, for each model, the accuracy rates in relation to the presence of diagrams and text volume were evaluated. Response data of the GPT-3.5 and GPT-4 models for all items in this study is available at Dataset 1.

### Bias

There was no bias in the prompt input.

### Study size

There was no need to calculate the study size. Only 2 generative artificial intelligence platforms were used.

### Statistical methods

The accuracy rates of both ChatGPT models were statistically compared using the chi-square test. Statistical tests were conducted at a 5% significance level and calculated using R software (The R Foundation for Statistical Computing).

## Results

In the GPT-3.5 model, 284 items were evaluated, excluding items that contained diagrams. The overall accuracy rate was 43.5% (124/285). Details by category and subject are presented in Table 1. Because the GPT-3.5 model did not meet the required accuracy rates for both the essential and general items, it failed to pass the 107th JNLEP. Conversely, it achieved a passing rate of more than 30% accuracy for all subjects for the essential items (based on the items that could be input). For the subjects of physics, chemistry, and biology, which frequently feature diagrammatic items, the number of items that could be input into GPT-3.5 was restricted.

In the GPT-4 model, all items, including those with diagrams, were inputted, and the overall accuracy rate was 72.5% (250/345 answers). When limited to items without diagrams for comparison with GPT-3.5, the overall accuracy rate was 80.0% (228/285) (Table 2). The GPT-4 model met all the passing criteria and successfully passed the 107th JNLEP. Compared with GPT-3.5, the GPT-4 model demonstrated exceptionally high performance (P<0.001, chi-square test) in the knowledge required for pharmacists in Japan. For GPT-4, the accuracy rates for subjects such as physics, chemistry, and pharmaceuticals were below the required minimum overall accuracy rate.

**Fig. 2.**
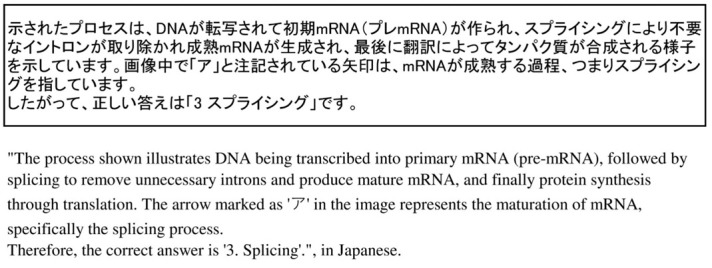
Example of GPT-4’s response to a question with a diagram (for the question in Fig. 1).

For items that included diagrams, GPT-4 was able to comprehend and respond to some of the items (Fig. 2); however, the accuracy rate was 36.1% (22/61 answers), indicating insufficient performance.

In GPT-3.5’s performance on 285 items without diagrams, high accuracy was noted in subjects such as regulations and pathophysiology (53.8% and 51.4%, respectively). In contrast, it showed lower accuracy for physics/chemistry/biology (34.4%), and practice (39.1%). For GPT-4, with 285 items, the subjects with high accuracy rates were pharmacology (92.3%) and practice (87.0%). Even for GPT-4, physics/chemistry/biology (65.6%) had the lowest accuracy rate. Of the 284 items without diagrams, the accuracy rate for the 146 single-choice items was 50.0% for GPT-3.5 and 87.7% for GPT-4. The accuracy rates for the 138 multiple-choice items were 35.5% and 72.5%, respectively.

## Discussion

### Key results

GPT-4 answered all items in the 107th JNLEP and passed. In the upgraded GPT-4 (September 2023 release), it was possible to answer items that included diagrams.

### Interpretation

The overall accuracy of the GPT-4 model for the 107th JNLEP was 72.5%. Compared with the GPT-3.5 model, there was a significant improvement in performance. This is likely because GPT-4 was trained using a more complex neural network and larger training dataset than GPT-3.5. The overall average accuracy rate of the major candidates (out of a total of 14,124 examinees, the results of 12,116 who reported their self-scoring) in the 107th JNLEP was 68.2% [11] and the results achieved by GPT-4 in this study exceeded the human average.

For the limited question set, excluding images, the overall accuracy rate was 43.5% for GPT-3.5 and 80.0% for GPT-4. The GPT-4 model met all passing criteria, but the GPT-3.5 model did not meet the criteria (overall accuracy rate and accuracy rate for essential items) and did not pass the 107th JNLEP.

For GPT-4, the accuracy rate for items containing diagrams (36.1%) was substantially lower than that for items without diagrams (80.0%). The theoretical accuracy rate for randomly selecting 1 of 5 choices was 20%, and GPT-4 exceeded this. However, for some items, there were instances in which no answer was input because of a failure to understand the diagram, indicating that further improvements in image comprehension abilities are expected in future versions of ChatGPT.

Physics/chemistry/biology had the lowest accuracy rate in both GPT-3.5 (34.4%) and GPT-4 (65.6%). These subjects involve many problems that require calculations and an understanding of relationships. Although GPT-4 has improved accuracy over GPT-3.5, it has been suggested that there are fields that have not been fully overcome. In contrast, practice had one of the lowest accuracy rates in GPT-3.5 (39.1%), but it had a very high accuracy rate in GPT-4 (87.0%). The items in the practice subject were complex, applied problems, and had a high text volume. The GPT-4 model, trained with considerably more complex parameters than GPT-3.5, has an improved ability to understand the context of long sentences. This is considered to be the reason why the accuracy rate in the practice subject greatly improved in GPT-4.

### Comparison with previous studies

In previous studies on the performance of ChatGPT on medical exams, the GPT-4 model was reported to have high accuracy rates on various tests, including the Peruvian Medical Licensing Examination (86.0% [4]), Japanese Medical Licensing Examination (JMLE) (81.5% [7], 79.9% [8], 82.8% [12], 78.6% [12]), Japanese Nursing Licensing Examination (79.7% [6]), and the JNLEP (78.2% [9], 75.3% [9]). The GPT-4 accuracy rates in this study are in line with these results, at 72.5% (all items) and 80.0% (without diagrams).

Using the upgraded GPT-4 model, this study evaluated all items on the JNLEP, including those with diagrams. There have been several reports of GPT-4 passing medical licensing exams; however, items containing diagrams were excluded in all those studies [6-9,12,13]. In the literature evaluating the performance of GPT-4 on the JMLE, the number of items that could be input varied depending on the study. For example, for the 117th JMLE conducted in 2023 (a total of 400 items), Takagi et al. [8] input 254 items, Tanaka et al. [12] entered 290 items, and Kaneda et al. [13] input 389 items. Because the passing criteria and average response rate of candidates for text-only items are unknown, it is difficult to compare the accuracy rate of ChatGPT with human capabilities on a limited set of items.

Kunitsu [9] reported the performance of GPT-4 for both the 107th and 108th JNLEP, and excluded items containing diagrams. The number of items input for the 107th JNLEP was the same as that used in this study for the GPT-3.5 model, totaling 284 items. Kunitsu [9] reported an overall accuracy rate of 78.2% (222/284), which is slightly lower than that obtained for our limited set of items, at 80.0% (228/285). It is known that ChatGPT can provide different responses even when the same prompt is input. It is unclear whether this difference in accuracy rates is due to the phenomenon controlled by the “temperature” parameter, or if it results from updates to the GPT-4 model because the study of Kunitsu [9] did not disclose the input date.

This study used input items from the original Japanese text. Three studies compared ChatGPT’s (probably GPT-3.5 model based on the date of publication) performance on the same examination in English and another language, all showing a higher accuracy rate with English language inputs: on the Taiwan Pharmacist License Examination [5], in Chinese (54.5%) versus English (63.5%); the JMLE [12] in Japanese (52.8%) versus English (56.2%); and on the Israeli Hebrew OBGYN National Residency Examinations [14], in Hebrew (38.7%) versus English (60.7%). Furthermore, it has been confirmed that tuning the input prompts also improves the accuracy rate [12]. In the context of JNLEP, there is also potential for higher performance by improving the input prompts (translation to English and/or prompt engineering).

ChatGPT has not been specifically trained using medical expertise. However, the GPT-4 model, in particular, has been confirmed to possess sufficient knowledge to pass national examinations in the pharmacy field.

### Limitations

In recent years, there have been remarkable improvements in the accuracy of LLMs, and ChatGPT has been frequently upgraded. Even within the same ChatGPT model, the accuracy may vary depending on the timing of the input test, owing to such updates. This study involved different input timings for the GPT-3.5 (February 2023) and GPT-4 models (November 2023). It is possible that the performance of the latest GPT-3.5 model has improved compared with the results of this study.

### Conclusion

ChatGPT (the upgraded GPT-4 model) has been confirmed to have the capability to input all items of the national pharmacist examination in Japan and meet the passing criteria. It appears to be a potentially powerful support tool for healthcare professionals and their students, when used with an understanding of its features and limitations.

## Figures and Tables

**Figure f3-jeehp-21-04:**
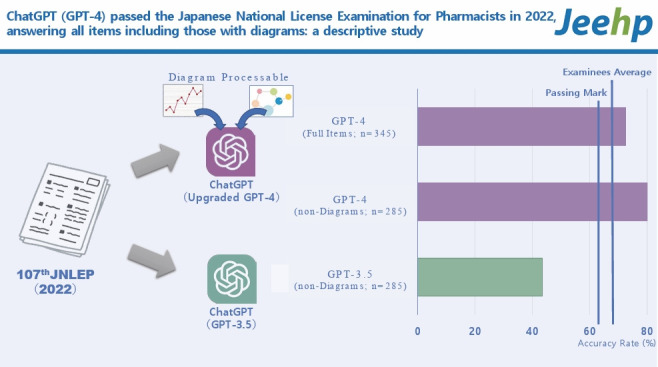


**Table 1.** The results for answers in the 107th Japanese National License Examination for Pharmacists (JNLEP) by GPT-3.5

Variable	Total	Physics	Chemistry	Biology	Hygiene	Pharmacology	Pharmaceutics	Pathophysiology	Regulations	Practice
Essential items										
No. of all items	90	5	5	5	10	15	15	15	10	10
No. of entered items	74	3	1	2	8	15	12	14	9	10
No. of correct answers	46	2	1	1	5	5	7	12	7	6
Accuracy rate 1 (%)	62.2	66.7	66.7	66.7	62.5	33.3	58.3	85.7	77.8	60.0
Accuracy rate 2 (%)	51.1	26.7	26.7	26.7	50.0	33.3	46.7	80.0	70.0	60.0
Passing criteria (%)	≥70	≥30	≥30	≥30	≥30	≥30	≥30	≥30	≥30	≥30
General items										
No. of all items^a)^	255	15	15	15	30	25	25	25	20	85
No. of entered items	211	13	5	8	19	24	18	23	19	82
No. of correct answers^a)^	78	3	0	4	8	13	5	7	8	30
Accuracy rate 1 (%)	37.0	26.9	26.9	26.9	42.1	54.2	27.8	30.4	42.1	36.6
Accuracy rate 2^a^ (%)	30.6	15.6	15.6	15.6	26.7	52.0	20.0	28.0	40.0	35.3
Passing criteria (%)	-	-			-	-	-	-	-	-
Overall items										
No. of all items^a)^	345	20	20	20	40	40	40	40	30	95
No. of entered items	285	16	6	10	27	39	30	37	28	92
N. of correct answers^a)^	124	5	1	5	13	18	12	19	15	36
Accuracy rate 1 (%)	43.5	34.4	34.4	34.4	48.1	46.2	40.0	51.4	53.6	39.1
Accuracy rate 2^a)^ (%)	35.9	18.3	18.3	18.3	32.5	45.0	30.0	47.5	50.0	37.9
Passing criteria (%)	≥62.9	-	-	-	-	-	-	-	-	-

Accuracy rate 1: correct answers/entered items; Accuracy rate 2: correct answers/all items.

a) Includes one question without a solution.

**Table 2.** The results of answers in the 107th Japanese National License Examination for Pharmacists (JNLEP) by GPT-4

Variable	Total	Physics	Chemistry	Biology	Hygiene	Pharmacology	Pharmaceutics	Pathophysiology	Regulations	Practice
Essential items										
No. of all items	90	5	5	5	10	15	15	15	10	10
No. of entered items	90	5	5	5	10	15	15	15	10	10
No. of non-diagram items	74	3	1	2	8	15	12	14	9	10
No. of correct answers 1	73	4	1	3	9	15	13	13	6	9
No. of correct answers 2	67	2	1	2	8	15	11	13	6	9
Accuracy rate 1 (%)	81.1	53.3	53.3	53.3	90.0	100.0	86.7	86.7	60.0	90.0
Accuracy rate 2 (%)	90.5	83.3	83.3	83.3	100.0	100.0	91.7	92.9	66.7	90.0
Passing criteria (%)	≥70	≥30	≥30	≥30	≥30	≥30	≥30	≥30	≥30	≥30
General items										
No. of all items^a)^	255	15	15	15	30	25	25	25	20	85
No. of entered items	254	15	15	15	30	25	25	25	20	85
No. oof non-diagram items	211	13	5	8	19	24	18	23	19	82
No. of correct answers 1^a)^	177	7	6	10	19	21	12	15	14	73
No. of correct answers 2	161	6	4	6	15	21	10	14	14	71
Accuracy rate 1^a)^ (%)	69.4	51.1	51.1	51.1	63.3	84.0	48.0	60.0	70.0	85.9
Accuracy rate 2 (%)	76.3	61.5	61.5	61.5	78.9	87.5	55.6	60.9	73.7	86.6
Passing criteria (%)	-	-	-	-	-	-	-	-	-	-
Overall items										
No. of all items^a)^	345	20	20	20	40	40	40	40	30	95
No. of entered items	344	20	20	20	40	40	40	40	30	95
No. of non-diagram items	285	16	6	10	27	39	30	37	28	92
No. of correct answers 1^a)^	250	11	7	13	28	36	25	28	20	82
No. of correct answers 2	228	8	5	8	23	36	21	27	20	80
Accuracy rate 1^a)^ (%)	72.7	51.7	51.7	51.7	70.0	90.0	62.5	70.0	66.7	86.3
Accuracy rate 2 (%)	80.0	65.6	65.6	65.6	85.2	92.3	70.0	73.0	71.4	87.0
Passing criteria (%)	≥62.9	-	-	-	-	-	-	-	-	-

Accuracy rate 1: correct answers/all items; Accuracy rate 2: correct answers/non-diagram items.

a) Includes 1 question without a solution.

## Footnotes

**Data availability**
Data files are available from Harvard Dataverse: https://doi.org/10.7910/DVN/NDOXMJ Dataset 1. Response data of the GPT-3.5 and GPT-4 models for all items in this study.

## Supplementary materials

Supplementary files are available from Harvard Dataverse: https://doi.org/10.7910/DVN/NDOXMJ

Supplement 1. The essential items of the 107th Japanese National License Examination for Pharmacists (JNLEP) (in Japanese).

Supplement 2. The general items of the 107th Japanese National License Examination for Pharmacists (JNLEP) (in Japanese).

Supplement 3. The correct answers of the 107th Japanese National License Examination for Pharmacists (JNLEP) (in Japanese).

Supplement 4. Audio recording of the abstract.
