# Supplementary material for: ChatGPT (GPT-4) passed the Japanese National License Examination for Pharmacists in 2022, answering all items including those with diagrams: a descriptive study
Source: J Educ Eval Health Prof. 2024 Feb 28;21:4. doi: 10.3352/jeehp.2024.21.4 (PMC10948916; doi:10.3352/jeehp.2024.21.4)
Supplement: Supplementary file 2 — Supplement 1. The essential items of the 107th Japanese National License Examination for Pharmacists (JNLEP) (in Japanese). [file jeehp-21-04-suppl1.pdf]

107　１日目①　　必須問題

【物理･化学･生物、衛生、薬理、薬剤、病態･薬物治療、法規･制度･倫理、実務】

◎指示があるまで開いてはいけません。

注　意　事　項

1　試験問題の数は、**問１から問９０までの９０問**。
９時３０分から１１時までの９０分以内で解答すること。

2　解答方法は次のとおりである。

⑴　必須問題の各問題の正答数は、**1つ**である。
　問題の選択肢の中から答えを１つ選び、次の例にならって答案用紙に記入すること。なお、**２つ以上解答すると、誤りになる**から注意すること。

（例）**問 400**　次の物質中、常温かつ常圧下で液体のものはどれか。**1つ**選べ。

1　塩化ナトリウム　　2　プロパン　　3　ナフタレン
4　エタノール　　5　炭酸カルシウム

正しい答えは「4」であるから、答案用紙の

問 400
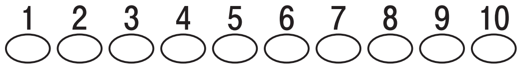

のうち 4 を塗りつぶして

問 400
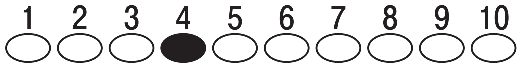

とすればよい。

⑵　解答は、　の中全体をＨＢの鉛筆で濃く塗りつぶすこと。塗りつぶしが薄い場合は、解答したことにならないから注意すること。

悪い解答例
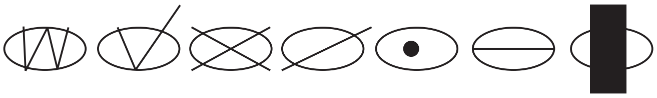
（採点されない）

⑶　解答を修正する場合は、必ず「消しゴム」で跡が残らないように完全に消すこと。鉛筆の跡が残ったり、「　」のような消し方などをした場合は、修正又は解答したことにならないから注意すること。

⑷　答案用紙は、折り曲げたり汚したりしないよう、特に注意すること。

3　設問中の科学用語そのものやその外国語表示（化合物名、人名、学名など）には誤りはないものとして解答すること。ただし、設問が科学用語そのもの又は外国語の意味の正誤の判断を求めている場合を除く。

4　問題の内容については質問しないこと。

必須問題　【物理・化学・生物】

問 1　0.010 mol/L 水酸化ナトリウム水溶液の pH として最も近い値はどれか。**1 つ**選べ。ただし、水のイオン積 $K_w = [H^+][OH^-] = 1.0 \times 10^{-14}(mol/L)^2$ とする。

1　1
2　2
3　7
4　12
5　13

問 2　平衡状態にある次の化学反応系に関する記述のうち、正しいのはどれか。**1 つ**選べ。

$$\frac{3}{2}H_2(g) \quad + \quad \frac{1}{2}N_2(g) \rightleftharpoons NH_3(g) \qquad \Delta_f H^\circ = -46.1\ kJ/mol$$

$\Delta_f H^\circ$ は標準生成エンタルピー、(g)は気体状態を表す。

1　系の温度を下げると、平衡は右側へ移動する。
2　系の圧力を下げると、平衡は右側へ移動する。
3　系に水素ガスを加えると、平衡は左側へ移動する。
4　この反応は吸熱反応である。
5　この反応の平衡定数は系の温度に依存しない。

問 3　図は、電位の基準となる標準水素電極の模式図である。図中の空欄 ア にあてはまる数値はどれか。**1つ**選べ。なお、1 atm は 1.013×10^5 Pa を表す。

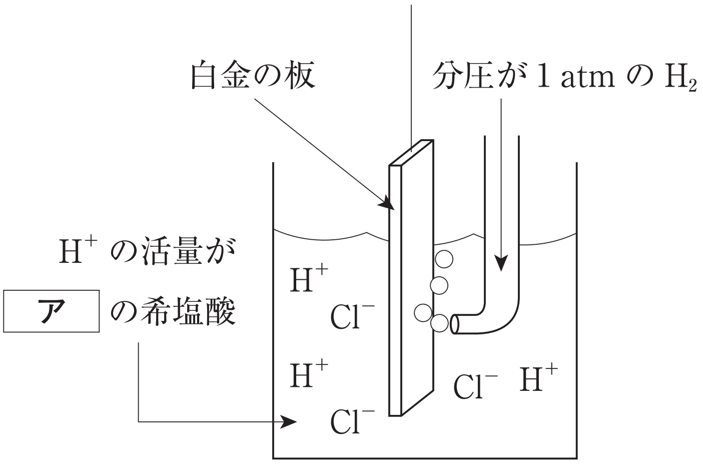

1　0
2　0.1
3　1
4　7
5　14

問 4　0.10 mol/L 硫酸ナトリウム水溶液中における硫酸バリウムの溶解度に最も近いのはどれか。**1つ**選べ。ただし、温度は 25 ℃とし、同温度における硫酸バリウムの溶解度積を 1.0×10^{-10} $(\mathrm{mol/L})^2$ とし、硫酸バリウムの溶解による溶液の体積変化は無視できるものとする。

1　1.0×10^{-19} mol/L
2　1.0×10^{-11} mol/L
3　1.0×10^{-9} mol/L
4　1.0×10^{-5} mol/L
5　1.0×10^{-4} mol/L

問 5　液体クロマトグラフィー/質量分析法において、用いられるイオン化法はどれか。**1 つ**選べ。

1　エレクトロスプレーイオン化（ESI）法
2　化学イオン化（CI）法
3　高速原子衝撃（FAB）法
4　電子イオン化（EI）法
5　マトリックス支援レーザー脱離イオン化（MALDI）法

問 6　3-メチルブタ-2-エン-1-オール（IUPAC 命名法）はどれか。**1 つ**選べ。

1

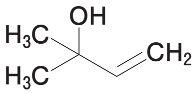

2

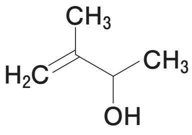

3

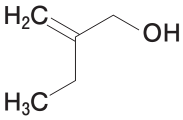

4

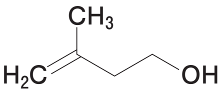

5

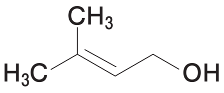

問 7　結合している原子に着目したとき、三角錐型の構造をもつのはどれか。**1 つ**選べ。

1

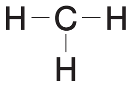

2

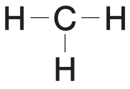

3

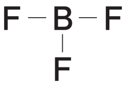

4

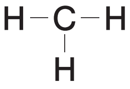

5

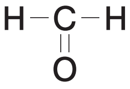

問 8　次の２つの薬物に関する記述のうち、正しいのはどれか。**1 つ**選べ。

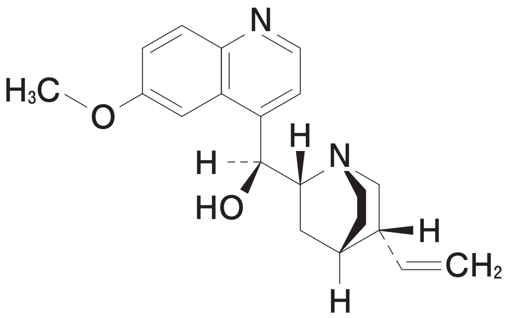

キニジン

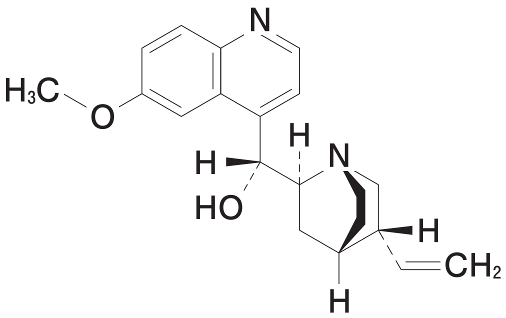

キニーネ

1　エナンチオマーの関係にある。

2　ジアステレオマーの関係にある。

3　構造異性体の関係にある。

4　融点が同じである。

5　比旋光度 $[\alpha]_{D}^{20}$ の絶対値が同じである。

問 9　不対電子を１つもつのはどれか。**1 つ**選べ。

1　CO

2　NO

3　SO_3

4　O_2

5　N_2

問 10　トリプトファン由来のアルカロイドはどれか。1つ選べ。

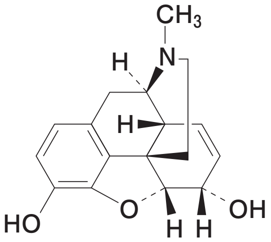

1

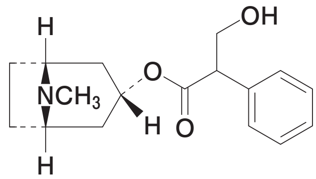

2

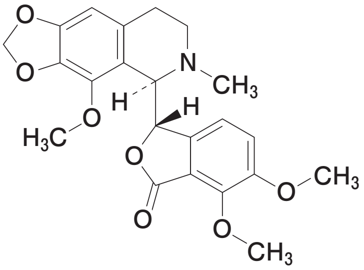

3

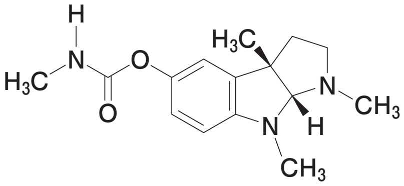

4

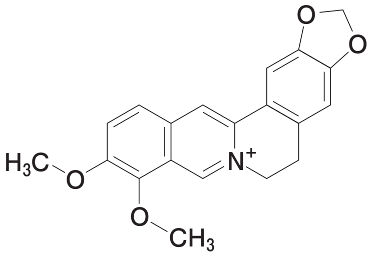

5

問 11　女性の月経周期において、排卵直前に分泌量が急激に増加してピークに達し、排卵の開始を誘発するホルモンはどれか。1つ選べ。

1　黄体形成ホルモン（LH）
2　オキシトシン
3　プロラクチン
4　ヒト絨毛性性腺刺激ホルモン（hCG）
5　プロゲステロン

問 12　尿素回路の代謝中間体であるオルニチンはどれか。**1つ**選べ。

1

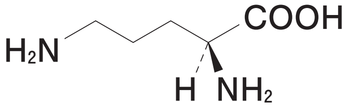

2

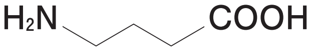

3

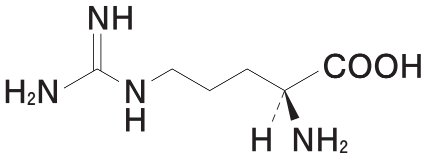

4

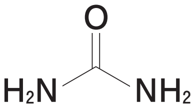

5

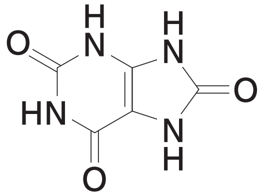

問 13　図は、真核生物においてDNA上の遺伝子からタンパク質が作られるまでの過程を示している。矢印 ア で示す反応はどれか。**1つ**選べ。

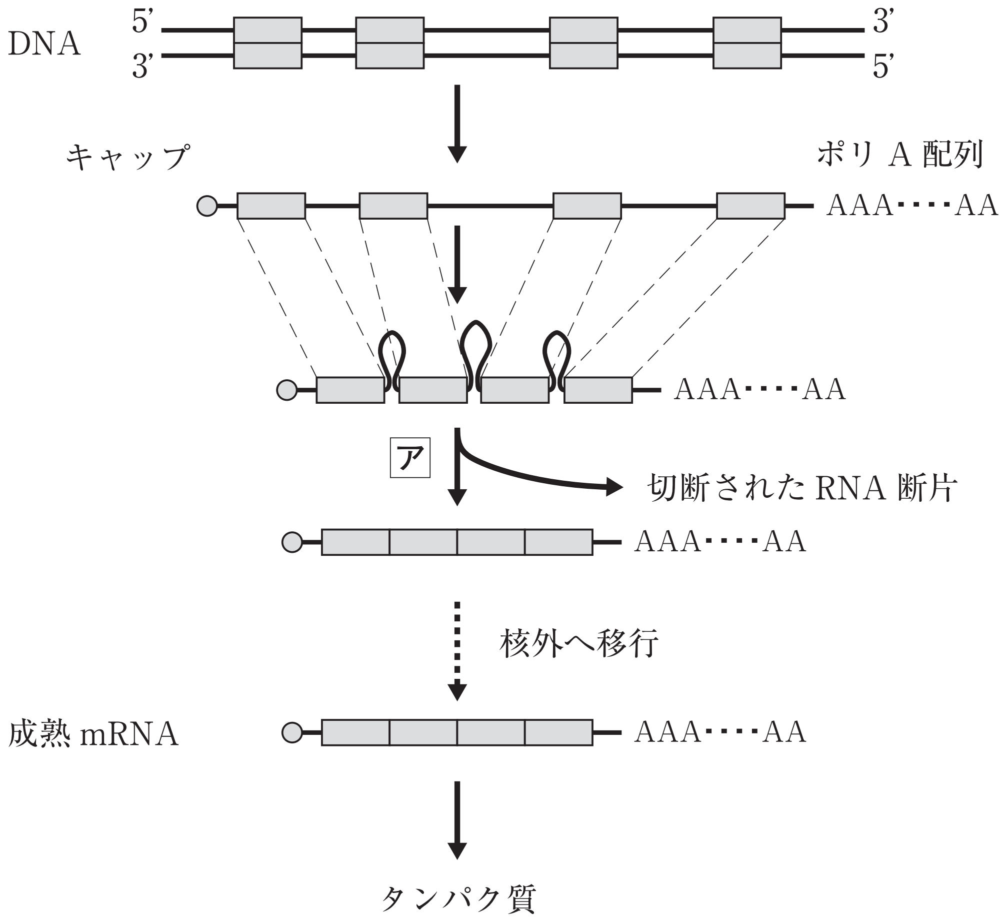

1　転写
2　逆転写
3　スプライシング
4　RNA干渉
5　翻訳

問 14　図は、細胞分裂後期にある細胞の様子を示している。図中の A で示された構造体はどれか。1 つ選べ。

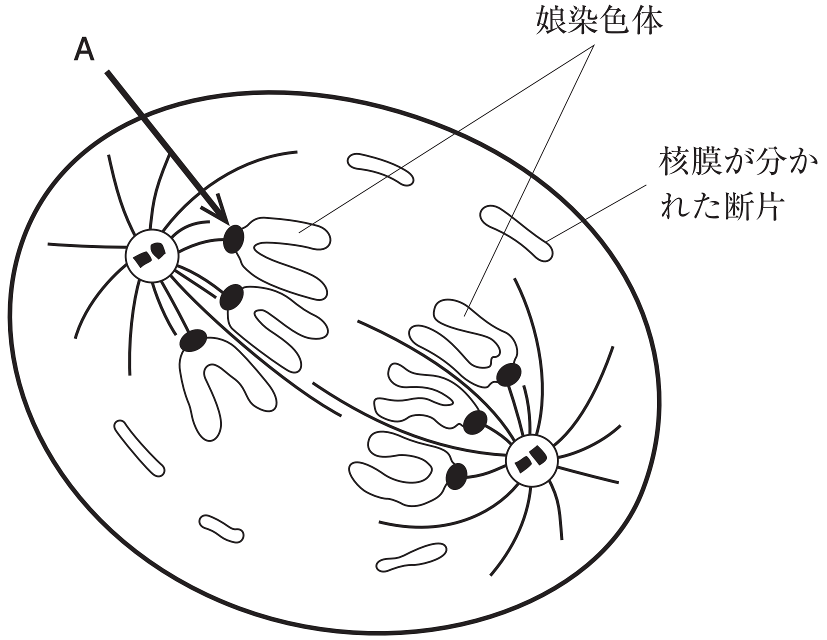

1　紡錘体
2　中心体
3　核小体
4　動原体
5　収縮環

問 15　リケッチアを病原体とする感染症はどれか。1 つ選べ。

1　梅毒
2　熱帯熱マラリア
3　オンコセルカ症（河川盲目症）
4　ツツガムシ病
5　日本脳炎

必須問題　【衛生】

問 16　体格指数の１つである BMI を算出する式はどれか。**１つ**選べ。

1　$\frac{身長（m）}{体重（kg）}$　　**2**　$\frac{身長（m）}{[体重（kg）]^2}$　　**3**　$\frac{体重（kg）}{身長（m）}$

4　$\frac{[体重（kg）]^2}{身長（m）}$　　**5**　$\frac{体重（kg）}{[身長（m）]^2}$

問 17　自然毒のうち、魚介類による食中毒の原因となるのはどれか。**１つ**選べ。

1　アコニチン
2　ソラニン
3　アミグダリン
4　テトロドトキシン
5　チャコニン

問 18　食品表示法に基づき、用途名と物質名を併記する必要がある食品添加物はどれか。**１つ**選べ。

1　香料
2　甘味料
3　調味料
4　乳化剤
5　pH 調整剤

問 19　精白米の第一制限アミノ酸はどれか。**1 つ**選べ。

1　バリン
2　リシン
3　ロイシン
4　イソロイシン
5　トリプトファン

問 20　食品の腐敗を防ぐ方法として、誤っているのはどれか。**1 つ**選べ。

1　くん煙
2　冷凍保存
3　加湿
4　加熱
5　保存料の添加

問 21　図は、我が国の薬物事犯について、2010 年から 2019 年の法律別検挙人数を示したものである。法律 A ～ E は、覚醒剤取締法、大麻取締法、麻薬及び向精神薬取締法、あへん法、毒物及び劇物取締法のいずれかである。近年、法律 B による検挙人数が増加傾向にある。法律 B として正しいのはどれか。 1 つ選べ。

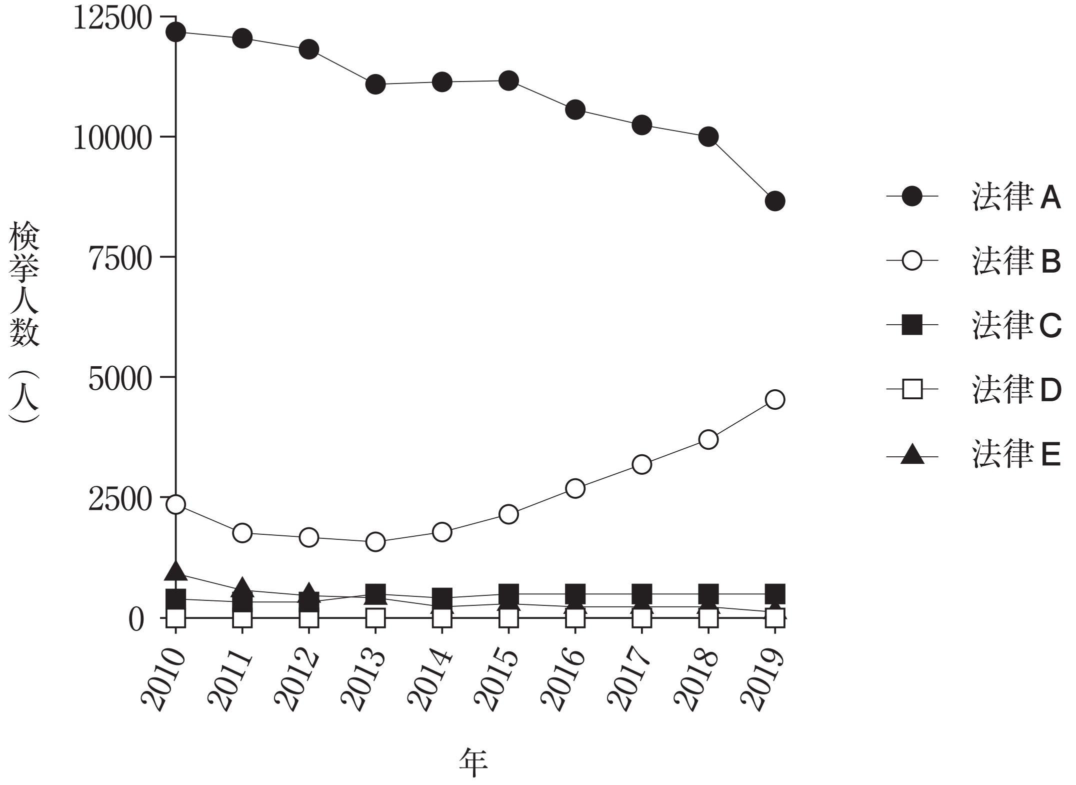

（注）覚醒剤取締法、大麻取締法、麻薬及び向精神薬取締法、あへん法違反の検挙人数は特別司法警察員が検挙した者を含む。
令和 2 年版 犯罪白書を基に作成

1　覚醒剤取締法
2　大麻取締法
3　麻薬及び向精神薬取締法
4　あへん法
5　毒物及び劇物取締法

問 22　化学物質のリスク分析において、「消費者、事業者、行政担当者などの関係者の間で情報及び意見を共有することで相互に意思疎通を図ること」を意味するのはどれか。**1つ**選べ。

1　リスク評価
2　リスク管理
3　リスクコミュニケーション
4　安全データシート（SDS）制度
5　マニフェスト制度

問 23　2-ナフチルアミンが生体内で代謝的活性化されてニトレニウムイオンを生じる過程において、最初に起こる代謝反応はどれか。**1つ**選べ。

1　エポキシ化
2　*N*-ヒドロキシ化
3　グルクロン酸抱合
4　硫酸抱合
5　アセチル抱合

問 24　公共用水域の水質汚濁に関する「人の健康の保護に関する環境基準」において、基準値が「検出されないこと」と定められているのはどれか。**1 つ**選べ。

1　カドミウム
2　ヒ素
3　アルキル水銀
4　トリクロロエチレン
5　硝酸性窒素及び亜硝酸性窒素

問 25　医療機関により廃棄される未使用の注射針が該当する区分として、最も適切なのはどれか。**1 つ**選べ。

1　事業系一般廃棄物
2　家庭系一般廃棄物
3　特別管理一般廃棄物
4　特別管理産業廃棄物
5　非感染性廃棄物

必須問題　【薬理】

問 26　Cl^- チャネル内蔵型受容体はどれか。**1つ**選べ。

1　アセチルコリン N_M 受容体
2　γ-アミノ酪酸 $GABA_A$ 受容体
3　セロトニン 5-HT_3 受容体
4　グルタミン酸 NMDA 受容体
5　ATP P2X 受容体

問 27　麻酔したラットにおいてフェントラミン処置後にアドレナリンを静脈内投与すると、アドレナリンの昇圧作用は認められず、降圧作用のみが見られた。この現象を生じさせたフェントラミンの機序はどれか。**1つ**選べ。

1　アドレナリン α_1 受容体刺激
2　アドレナリン α_1 受容体遮断
3　アドレナリン β_1 受容体刺激
4　アドレナリン β_1 受容体遮断
5　アドレナリン β_2 受容体遮断

問 28　メピバカインの局所麻酔作用の機序はどれか。**1つ**選べ。

1　ATP 感受性 K^+ チャネルの刺激
2　アセチルコリン N_N 受容体の遮断
3　電位依存性 Na^+ チャネルの遮断
4　セロトニン 5-HT_{1A} 受容体の遮断
5　電位依存性 T 型 Ca^{2+} チャネルの遮断

問 29　ミルタザピンがシナプス間隙のセロトニン及びノルアドレナリンを増加させる機序はどれか。1つ選べ。

1　アドレナリン α_2 受容体遮断
2　セロトニン 5-HT_{2A} 受容体刺激
3　セロトニン 5-HT_3 受容体刺激
4　モノアミン酸化酵素阻害
5　セロトニン及びノルアドレナリンの再取り込み阻害

問 30　抗てんかん薬ガバペンチンの作用点はどれか。1つ選べ。

1　電位依存性 Ca^{2+} チャネル
2　電位依存性 Na^+ チャネル
3　シナプス小胞タンパク質 SV2A
4　γ-アミノ酪酸 $GABA_A$ 受容体
5　グルタミン酸 AMPA 受容体

問 31　T 細胞のカルシニューリンを阻害する免疫抑制薬はどれか。1つ選べ。

1　シクロホスファミド
2　アザチオプリン
3　レフルノミド
4　バシリキシマブ
5　シクロスポリン

問 32　骨粗しょう症治療薬テリパラチドの作用点はどれか。**1 つ**選べ。

1　ヒドロキシアパタイト
2　オステオカルシン
3　カルシトニン受容体
4　副甲状腺ホルモン受容体
5　エストロゲン受容体

問 33　心筋収縮力及び心拍数を低下させ、労作性狭心症発作を予防するのはどれか。**1 つ**選べ。

1　硝酸イソソルビド
2　チクロピジン
3　ピモベンダン
4　アテノロール
5　デノパミン

問 34　プラスミンによるフィブリン溶解を抑制することで、止血作用を示すのはどれか。**1 つ**選べ。

1　トラネキサム酸
2　アルテプラーゼ
3　カルバゾクロム
4　プロタミン
5　フィトナジオン

問 35　利尿薬の作用機序でないのはどれか。1つ選べ。

1　バソプレシン V_2 受容体遮断
2　心房性ナトリウム利尿ペプチド（ANP）受容体刺激
3　アルドステロン受容体刺激
4　炭酸脱水酵素阻害
5　Na^+-K^+-$2Cl^-$ 共輸送系阻害

問 36　肺サーファクタント分泌を促進する去痰薬はどれか。1つ選べ。

1　オキシメテバノール
2　アセチルシステイン
3　カルボシステイン
4　ドルナーゼ アルファ
5　アンブロキソール

問 37　Cl^- チャネル 2（ClC-2）を活性化する慢性便秘症治療薬はどれか。1つ選べ。

1　センノシド
2　カルメロース
3　ラクツロース
4　ビサコジル
5　ルビプロストン

問 38　メチラポンによるコルチゾール産生抑制の機序はどれか。**1つ**選べ。

1　副腎皮質刺激ホルモン（ACTH）の分泌抑制
2　ソマトスタチンの分泌亢進
3　副腎皮質細胞壊死
4　3 β-ヒドロキシステロイド脱水素酵素の阻害
5　11 β-水酸化酵素の阻害

問 39　腎尿細管の尿酸トランスポーター（URAT1）を阻害して、尿酸再吸収を抑制するのはどれか。**1つ**選べ。

1　コルヒチン
2　フェブキソスタット
3　プロベネシド
4　アロプリノール
5　ラスブリカーゼ

問 40　細菌のリボソーム 30S サブユニットに結合して、タンパク質合成を阻害する抗菌薬はどれか。**1つ**選べ。

1　クリンダマイシン
2　ストレプトマイシン
3　リネゾリド
4　エリスロマイシン
5　クロラムフェニコール

必須問題　【薬剤】

問 41　下図のセファレキシンの消化管吸収に主として関与するトランスポーターはどれか。**1つ**選べ。

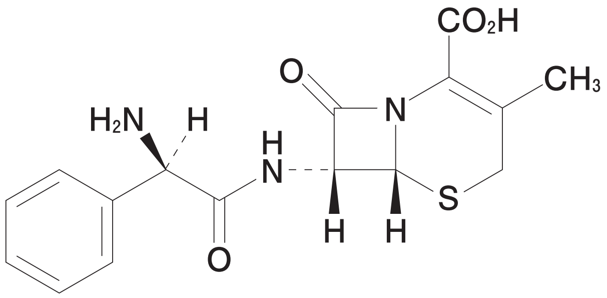

1　有機アニオントランスポーター OAT1

2　有機カチオントランスポーター OCT2

3　P-糖タンパク質

4　ペプチドトランスポーター PEPT1

5　グルコーストランスポーター SGLT2

問 42　アルミニウム、マグネシウム等の金属を含有する経口剤と同時に経口投与すると、吸収が低下する薬剤はどれか。**1つ**選べ。

1　オメプラゾール錠

2　レボフロキサシン錠

3　フェノバルビタール散

4　リボフラビン酪酸エステル錠

5　ワルファリンカリウム錠

問 43　血漿タンパク質のうち、プロプラノロールとの親和性が最も高いのはどれか。**1つ**選べ。

1　アルブミン

2　α-グロブリン

3　γ-グロブリン

4　フィブリノーゲン

5　α_1-酸性糖タンパク質

問 44　コデインからモルヒネへの代謝に関与する酵素はどれか。**1つ**選べ。

1　CYP1A2

2　CYP2C9

3　CYP2D6

4　CYP3A4

5　UGT1A1

問 45　体内動態が1-コンパートメントモデルに従う薬物800 mgをヒトに単回静脈内投与したところ、投与直後の血中濃度は40 μg/mL、投与6時間後の血中濃度は5 μg/mLであった。この薬物の消失速度定数（h^{-1}）に最も近い値はどれか。**1つ**選べ。ただし、ln2 = 0.69とする。

1　0.12

2　0.23

3　0.35

4　0.69

5　2.0

問 46　健常人におけるイヌリンの血漿中濃度と尿中排泄速度との関係を正しく表したグラフはどれか。**1** つ選べ。

1

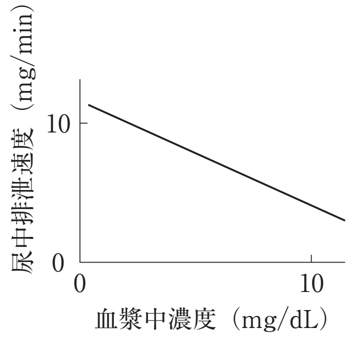

2

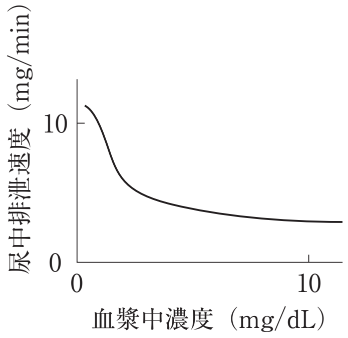

3

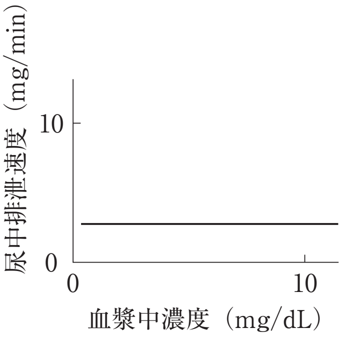

4

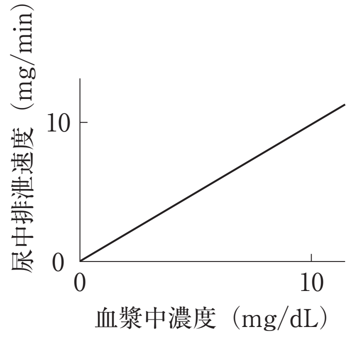

5

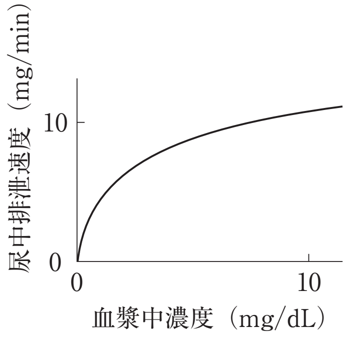

問 47　炭酸水素ナトリウムの併用によって、キニジンの血中濃度が上昇する原因として最も適切なのはどれか。**1** つ選べ。

1　消化管吸収の阻害
2　肝代謝酵素の阻害
3　胆汁排泄の促進
4　腎尿細管分泌の促進
5　腎尿細管再吸収の促進

問 48　図は、結晶固体及び非晶質固体の比容積と温度との関係を示したものである。温度**ア**が示すのはどれか。**1**つ選べ。

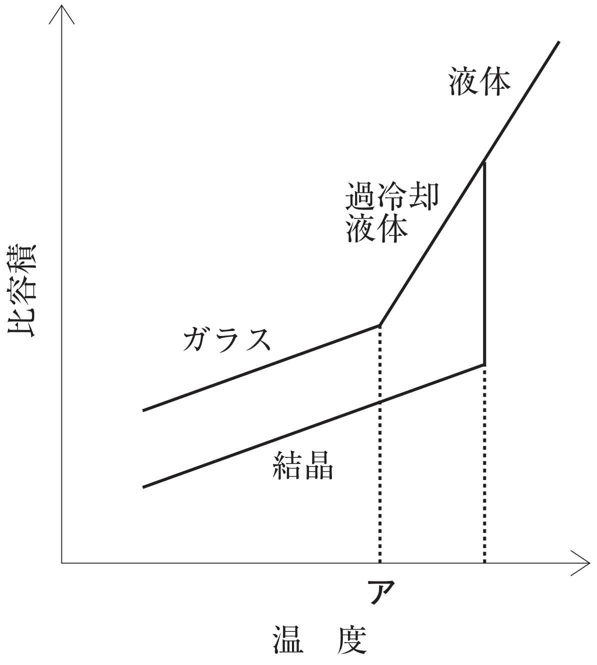

1　凝固点

2　沸点

3　ガラス転移点

4　融点

5　結晶化温度

問 49　分散系における分散相と分散媒の組合せのうち、正しいのはどれか。**1**つ選べ。

	性状	分散相	分散媒
1	サスペンション	固体	気体
2	エマルション	液体	液体
3	エアゾール	気体	液体又は固体
4	フォーム（泡沫）	液体	気体
5	キセロゲル	固体	液体

問 50　水性懸濁液中の粒子の分散安定性を高めるために添加される半合成高分子はどれか。**1つ**選べ。

1　ポビドン
2　メタクリル酸コポリマー
3　アルブミン
4　カルメロースナトリウム
5　アラビアゴム

問 51　日本薬局方において、「経口投与する、液状又は流動性のある粘稠なゲル状の製剤」と定義されているのはどれか。**1つ**選べ。

1　発泡顆粒剤
2　経口ゼリー剤
3　シロップ剤
4　口腔内崩壊フィルム剤
5　経口液剤

問 52　全身作用を目的としたナファレリン酢酸塩水和物製剤の適用部位として、最も適切なのはどれか。**1つ**選べ。

1　眼
2　肺
3　鼻
4　皮膚
5　耳

問 53　小児の誤飲防止を目的として用いられる包装はどれか。**1つ**選べ。

1　PTP（Press Through Package）包装
2　SP（Strip Package）包装
3　ピロー包装
4　タンパレジスタント包装
5　チャイルドレジスタンス包装

問 54　薬物送達システム（DDS）の概念に基づいて製剤を開発する際の利点として、誤っているのはどれか。**1つ**選べ。

1　病巣部位への薬物の集積
2　薬効の持続化
3　血液脳関門の透過性改善
4　腎排泄の増大
5　副作用の軽減

問 55　吸収促進剤の添加により水溶性薬物の吸収を改善した薬剤はどれか。**1つ**選べ。

1　バラシクロビル錠
2　セフチゾキシムナトリウム坐剤
3　イトラコナゾール内用液
4　シクロスポリン内用液
5　カンデサルタン シレキセチル錠

必須問題　【病態・薬物治療】

問 56　全身性エリテマトーデス（SLE）に特異性の高い抗体はどれか。**1 つ**選べ。

1　抗チログロブリン抗体（抗サイログロブリン抗体）
2　抗二本鎖 DNA 抗体
3　抗 CCP 抗体（抗環状シトルリン化ペプチド抗体）
4　抗 Jo-1 抗体
5　抗 GAD 抗体（抗グルタミン酸デカルボキシラーゼ抗体）

問 57　間質性肺炎の指標として、特異度が高いのはどれか。**1 つ**選べ。

1　アミラーゼ（AMY）
2　脳性ナトリウム利尿ペプチド（BNP）
3　クレアチンキナーゼ（CK）
4　グリコヘモグロビン（HbA1c）
5　シアル化糖鎖抗原（KL-6）

問 58　重症筋無力症の初発症状として、最も頻度が高いのはどれか。**1 つ**選べ。

1　手指の振戦
2　嚥下障害
3　四肢の麻痺
4　眼瞼下垂
5　発熱

問 59　健常者におけるレム睡眠に関する記述のうち、正しいのはどれか。**1つ**選べ。

1　急速な眼球運動が特徴である。
2　筋肉の緊張が亢進する。
3　入眠直後に多い。
4　脳波で高振幅徐波を認める。
5　加齢とともに増加する。

問 60　自動車の運転等危険を伴う機械の操作に従事させないよう注意する薬剤はどれか。**1つ**選べ。

1　タムスロシン塩酸塩錠
2　クロルマジノン酢酸エステル錠
3　セルニチンポーレンエキス錠
4　フルタミド錠
5　エストラムスチンリン酸エステルナトリウム水和物カプセル

問 61　閃輝暗点(せんきあんてん)を伴うことがある頭痛はどれか。**1つ**選べ。

1　片頭痛
2　反復性緊張型頭痛
3　慢性緊張型頭痛
4　反復性群発頭痛
5　慢性群発頭痛

問 62　依存性の最も少ない薬物はどれか。1つ選べ。

1　ペンタゾシン
2　メチルフェニデート
3　グアンファシン
4　メタンフェタミン
5　ペモリン

問 63　子宮内膜症の治療に用いる薬剤はどれか。1つ選べ。

1　エチニルエストラジオール錠
2　エンザルタミド錠
3　オキシトシン注
4　リュープロレリン酢酸塩注
5　レトロゾール錠

問 64　急性胆管炎に関する記述のうち、正しいのはどれか。1つ選べ。

1　左下腹部に痛みを生じる。
2　発熱を伴うことはまれである。
3　血中白血球数が減少する。
4　血清 ALP（アルカリフォスファターゼ）活性が上昇する。
5　血中間接ビリルビン値が上昇する。

問 65　原発性アルドステロン症の臨床所見として、正しいのはどれか。**1つ**選べ。

1　低血圧症
2　低ナトリウム血症
3　低カリウム血症
4　インスリン分泌の増加
5　レニン活性の上昇

問 66　活性型ビタミン D_3 外用薬が適用される皮膚疾患はどれか。**1つ**選べ。

1　接触性皮膚炎
2　爪白癬
3　アトピー性皮膚炎
4　じん麻疹
5　尋常性乾癬

問 67　がん終末期における呼吸困難に対する治療薬はどれか。**1つ**選べ。

1　アセトアミノフェン
2　アドレナリン
3　スキサメトニウム
4　デキストロメトルファン
5　モルヒネ

問 68　組換え体医薬品<u>でない</u>のはどれか。**1つ**選べ。

1　アルテプラーゼ
2　カルペリチド
3　ソラフェニブ
4　ニボルマブ
5　レノグラスチム

問 69　厚生労働省が発行する資料はどれか。**1つ**選べ。

1　医療用医薬品添付文書
2　医薬品インタビューフォーム
3　医薬品リスク管理計画
4　医薬品・医療機器等安全性情報
5　くすりのしおり

問 70　被験者 100 人について、ある臨床検査値 **X** を調べた時のヒストグラムが以下のようになった。同じデータに基づいて作成した箱ひげ図として、妥当なのはどれか。**1 つ**選べ。

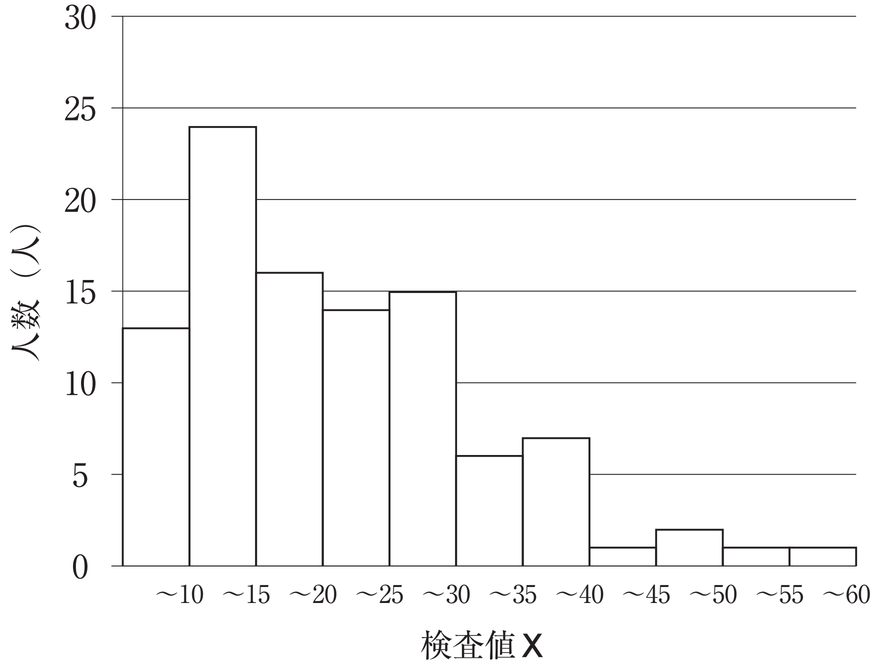

1

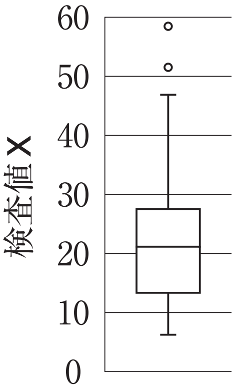

検査値X
60
50
40
30
20
10
0

2

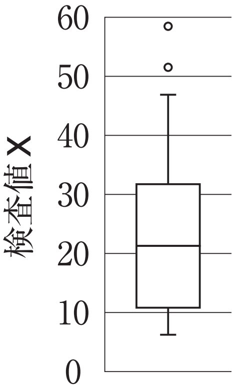

検査値X
60
50
40
30
20
10
0

3

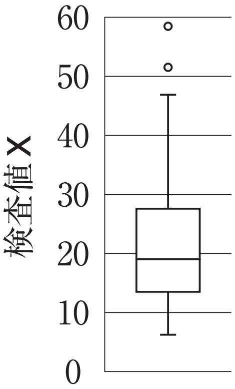

検査値X
60
50
40
30
20
10
0

4

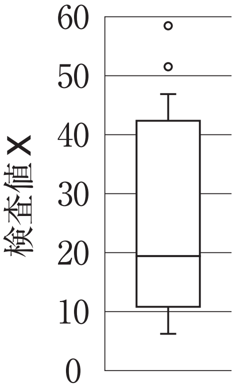

検査値X
60
50
40
30
20
10
0

5

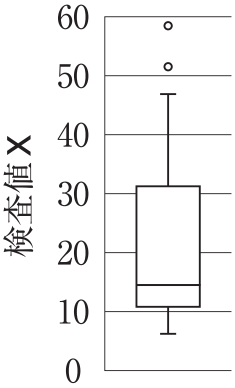

検査値X
60
50
40
30
20
10
0

必須問題　【法規・制度・倫理】

問 71　調剤済みとなった処方箋に薬剤師が記載しなければならないのはどれか。**1つ**選べ。

1　患者の病名
2　患者の住所
3　調剤年月日
4　調剤した薬剤師の薬剤師名簿登録番号
5　服薬指導の内容

問 72　新有効成分薬物などの初回治験計画を届け出る場合、保健衛生上の危害の発生を防止するために、厚生労働大臣が必要な調査を行う期間が設けられている。そのため、届出した日から（**ア**）日を経過した後でなければ、治験を依頼し、又は自ら実施してはならない。（**ア**）にあてはまる数値として正しいのはどれか。**1つ**選べ。

1　3
2　7
3　15
4　30
5　60

問 73　GVP 省令に基づき、新医薬品の適正使用のための情報提供と副作用情報の把握のために、市販後のある一定期間、製造販売業者が行う調査はどれか。**1つ**選べ。

1　一般使用成績調査
2　製造販売後臨床試験
3　市販直後調査
4　特定使用成績調査
5　使用成績比較調査

問 74　薬害事案として、血友病患者への非加熱血液製剤の使用が原因となり生じたのはどれか。**1つ**選べ。

1　HIV 感染症
2　クロイツフェルト・ヤコブ病
3　アザラシ肢症
4　スモン病
5　クロロキン網膜症

問 75　毒物劇物営業者が行う毒物の容器及び被包への表示方法として、正しいのはどれか。**1つ**選べ。

1　「医薬用外」の文字及び白地に黒色をもって「毒物」の文字
2　「医薬用外」の文字及び黒地に白色をもって「毒物」の文字
3　「医薬用外」の文字及び赤地に白色をもって「毒物」の文字
4　「医薬用外」の文字及び白地に赤色をもって「毒」の文字
5　「医薬用外」の文字及び白地に黒色をもって「毒」の文字

問 76　次のうち、麻薬及び向精神薬取締法に基づく「証紙による封かん」の封が施されていない麻薬を譲り渡すことができる業者はどれか。**1つ**選べ。

1　麻薬輸入業者
2　麻薬製造業者
3　麻薬元卸売業者
4　麻薬卸業者
5　麻薬小売業者

問 77　図は、保険医療における医薬品供給の流れの一例を示している。薬価どおりの価格による取引はどれか。**1つ**選べ。

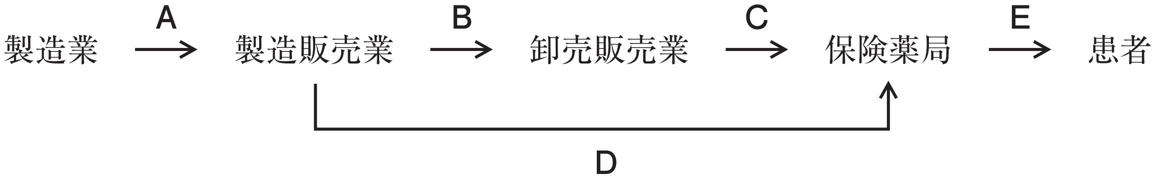

1　A
2　B
3　C
4　D
5　E

問 78　2018 年に制定された「薬剤師行動規範」の内容として、適切でないのはどれか。**1つ**選べ。

1　患者の自己決定権の尊重
2　差別の排除
3　医療資源の公正な配分
4　収益性を優先した医薬品供給
5　国民の主体的な健康管理への支援

問 79　ニュルンベルク綱領を踏まえ「人間を対象とする医学研究の倫理的原則」を内容とする、世界医師会が採択した宣言はどれか。**1つ**選べ。

1　リスボン宣言
2　ヘルシンキ宣言
3　世界人権宣言
4　ジュネーブ宣言
5　ポツダム宣言

問 80　学校薬剤師の設置が法律で義務付けられていないのはどれか。**1つ**選べ。

1　幼稚園
2　小・中学校
3　高等学校
4　大学
5　特別支援学校

必須問題 【実務】

問 81 「インフォームド・コンセント」に関する記述のうち、正しいのはどれか。**1 つ**選べ。

1 治験の被験者に対してのみ実施する。
2 同意後には撤回できない。
3 同意後には患者情報は個人名で一般公開する。
4 本人に意思決定能力があれば、患者の主体性を重んじる。
5 患者の心情に配慮するため、不安を与える内容は伝えない。

問 82 以下の説明文に該当するのはどれか。**1 つ**選べ。

厚生労働省において、2025 年を目途に、高齢者の尊厳の保持と自立生活の支援の目的のもとで、可能な限り住み慣れた地域で、自分らしい暮らしを人生の最期まで続けることができるような仕組みを構築することを推進している。

1 地域包括ケアシステム
2 地域医療情報システム
3 地域連携クリニカルパス
4 健康サポート薬局
5 かかりつけ薬局

問 83 薬学的管理の経過を SOAP 形式で記録する場合、「O」の項目に記載する内容として、適切なのはどれか。**1 つ**選べ。

1 医師への処方提案内容
2 患者の自覚症状
3 投与方法の妥当性の評価
4 薬に対する患者の訴え
5 血中薬物濃度の測定値

問 84　循環血液量の減少時における細胞外液の補給に用いる輸液として、<u>適切でない</u>のはどれか。**1つ**選べ。

1　生理食塩水
2　５％ブドウ糖液
3　乳酸リンゲル液
4　酢酸リンゲル液
5　５％ブドウ糖加乳酸リンゲル液

問 85　抗がん剤の無菌調製に関する記述のうち、適切なのはどれか。**1つ**選べ。

1　調製者は、手袋を二重に装着する。
2　調製作業は、クリーンベンチ内で行う。
3　調製に使用するシリンジの先端部は、ルアースリップ式を用いる。
4　バイアルから薬液を吸引する場合は、バイアル内を陽圧にする。
5　作業台が汚染した場合は、ペーパータオルで中心から外側に向かって拭き取る。

問 86　風しんの流行の原因となる主要な感染経路はどれか。**1つ**選べ。

1　食品媒介感染
2　昆虫媒介感染
3　飛沫感染
4　空気感染
5　垂直感染

問 87　薬剤師が糖尿病患者を訪問薬剤管理指導のために訪れた際、猛暑の中でぐったりしていたため脱水を疑いアセスメントをした。その項目として、適切でないのはどれか。**1つ選べ。**

1　口渇の有無

2　脇の下の乾燥

3　HbA1c 値

4　爪圧迫時の色調変化

5　脈拍

問 88　病院を退院した患者が在宅療養となった。かかりつけ薬剤師がその患者に居宅療養管理指導を行うために、居宅サービス計画書の作成を依頼する職種はどれか。**1つ選べ。**

1　医師

2　介護支援専門員

3　作業療法士

4　訪問看護師

5　訪問介護員

問 89　疾病の一次予防に該当するのはどれか。**1つ**選べ。

1　がん検診
2　がん患者への緩和ケア
3　うつ病患者に対する社会復帰支援
4　歩行機能低下患者に対する機能訓練
5　地域住民を対象とした健康教室

問 90　直近 10 年間の世界アンチドーピング規程において、禁止物質として指定されていない薬物はどれか。**1つ**選べ。

1　アセタゾラミド
2　エリスロポエチン
3　メチルテストステロン
4　カフェイン
5　メチルフェニデート
