# Supplementary material for: ChatGPT (GPT-4) passed the Japanese National License Examination for Pharmacists in 2022, answering all items including those with diagrams: a descriptive study
Source: J Educ Eval Health Prof. 2024 Feb 28;21:4. doi: 10.3352/jeehp.2024.21.4 (PMC10948916; doi:10.3352/jeehp.2024.21.4)
Supplement: Supplementary file 3 — Supplement 2. The general items of the 107th Japanese National License Examination for Pharmacists (JNLEP) (in Japanese). [file jeehp-21-04-suppl2.pdf]

107　**１日目②**　**一般問題（薬学理論問題）**

【物理・化学・生物、衛生、法規・制度・倫理】

◎指示があるまで開いてはいけません。

注　意　事　項

1　試験問題の数は、**問９１から問１５０までの６０問**。
１２時３０分から１５時までの１５０分以内で解答すること。

2　解答方法は次のとおりである。

(1)　一般問題（薬学理論問題）の各問題の正答数は、**問題文中に指示されている**。
問題の選択肢の中から答えを選び、次の例にならって答案用紙に記入すること。
なお、問題文中に指示された正答数と**異なる数を解答すると、誤りになる**から注意すること。

（例）**問 500**　次の物質中、常温かつ常圧下で液体のものはどれか。**２つ**選べ。

1　塩化ナトリウム　　**2**　プロパン　　**3**　ベンゼン
4　エタノール　　**5**　炭酸カルシウム

正しい答えは「**3**」と「**4**」であるから、答案用紙の

問 500 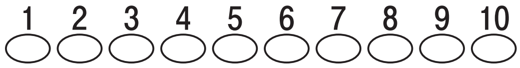 のうち 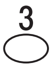 と 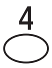 を塗りつぶして

問 500 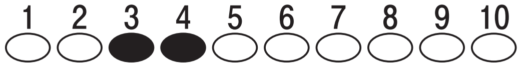 とすればよい。

(2)　解答は、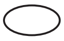 の中全体をＨＢの鉛筆で濃く塗りつぶすこと。塗りつぶしが薄い場合は、解答したことにならないから注意すること。

悪い解答例 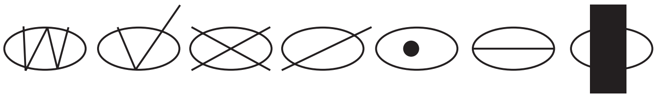 **（採点されない）**

(3)　解答を修正する場合は、必ず「消しゴム」で跡が残らないように完全に消すこと。鉛筆の跡が残ったり、「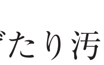」のような消し方などをした場合は、修正又は解答したことにならないから注意すること。

(4)　答案用紙は、折り曲げたり汚したりしないよう、特に注意すること。

3　設問中の科学用語そのものやその外国語表示（化合物名、人名、学名など）には誤りはないものとして解答すること。ただし、設問が科学用語そのもの又は外国語の意味の正誤の判断を求めている場合を除く。

4　問題の内容については質問しないこと。

一般問題（薬学理論問題）【物理・化学・生物】

問 91　分子軌道法に基づく基底状態の分子の電子配置に関する記述のうち、正しいのはどれか。２つ選べ。

1　電子は特定の原子に属さず、分子全体に広がっている。
2　電子は一つの軌道に何個でも入ることができる。
3　一つの軌道に同じ向きのスピンをもつ電子が複数入ることができる。
4　電子はエネルギーの高い軌道から優先的に入ることがある。
5　結合次数は、(結合性軌道の電子数－反結合性軌道の電子数)/2 で与えられる。

問 92　放射線及び放射壊変に関する記述のうち、正しいのはどれか。２つ選べ。

1　放射壊変には 0 次反応速度式に従う過程と、1 次反応速度式に従う過程の 2 通りがある。
2　放射能の SI 組立単位はベクレル（Bq）であり、その定義は 1 秒あたりに壊変する原子核数である。
3　β^- 壊変では、生成する電子とニュートリノにエネルギーが分配されるため、電子のもつエネルギーは連続的な分布を示す。
4　X 線と γ 線は電磁波であり、波長で区別されている。
5　γ 転移により放射される γ 線のエネルギーは、壊変する原子核種によらず一定である。

問 93　状態関数と経路関数に関する記述のうち、正しいのはどれか。<u>２つ</u>選べ。

1　熱と仕事は経路関数である。

2　温度は示量性の状態関数である。

3　エンタルピーは示強性の状態関数である。

4　熱力学第一法則より、内部エネルギーは経路関数であることがわかる。

5　状態関数の変化量は、可逆過程でも不可逆過程でも等しい。

問 94　生体膜の膜電位は、膜の両側におけるイオン濃度の不均衡によって生じる。そのイオン濃度の不均衡は、生体膜が水や小さいイオンは通すが、大きなイオンは通さない半透膜の性質をもつことで生じる。

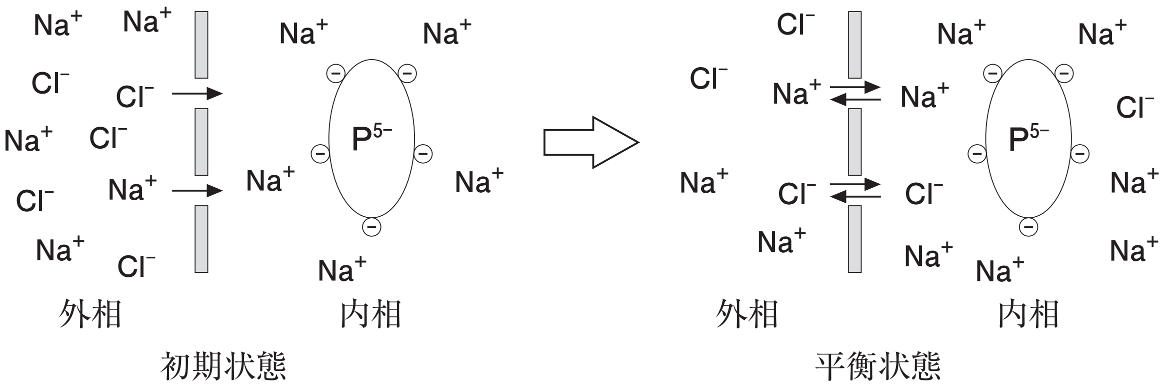

図のように、半透膜の内相にタンパク質 P^{5-}（−5 の電荷をもち 5 Na^+ が対イオンとなっている）の 0.01 mol/L 水溶液を置き、外相には濃度が 0.1 mol/L の NaCl 水溶液を置いておく。平衡状態に達したとき、半透膜の外相と内相の Na^+ と Cl^- の濃度には次式が成立している。

$$[Na^+]_{外相} \cdot [Cl^-]_{外相} = [Na^+]_{内相} \cdot [Cl^-]_{内相}$$

平衡に達したときの半透膜の内相と外相の Na^+ の濃度の差に最も近い値はどれか。**1** つ選べ。ただし、浸透圧差に基づく物質の移動は考慮しないものとする。

1　0.01 mol/L
2　0.03 mol/L
3　0.05 mol/L
4　0.07 mol/L
5　0.09 mol/L

問 95 **ア**～**ウ**のグラフは、反応次数の異なる化学反応の経時変化を表したものである。これらのグラフに関する記述のうち、正しいのはどれか。**2つ**選べ。ただし、［**A**］は反応物 **A** の濃度（懸濁液の場合は、その時点の、**A** の全量を体積で割った値)、t は時間を表す。

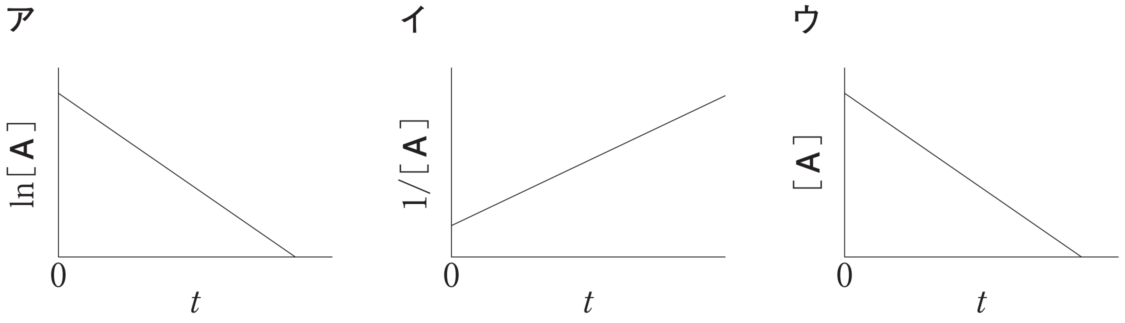

1 傾きから反応速度定数を求められるのは**ア**と**ウ**であるが、**イ**では求められない。

2 MRI 信号の減衰は**ア**のグラフと同じ変化を示す。

3 半減期がその時点での濃度によって変化しない反応は、**イ**のグラフを示す。

4 懸濁液中の加水分解反応で、反応速度よりも溶解速度が速い場合は、**ウ**のグラフを示す。

5 **ア**～**ウ**の反応速度定数の次元は同じである。

問 96 分配係数は、薬物の脂溶性の指標として用いられる。ある 1 価の弱酸 HA（pK_a 5.3）が pH 5.0 の緩衝液中に溶解している。この緩衝液 200 mL に水と混ざり合わない有機溶媒 100 mL を加えて HA を 1 回抽出したところ、抽出率は 75% であった。この弱酸 HA の分配係数 K_D（有機溶媒中の分子形の濃度 / 緩衝液中の分子形の濃度）に最も近い値はどれか。**1 つ**選べ。ただし、温度は一定で、混合により有機溶媒と緩衝液の体積に変化はなく、イオン形（解離形）は有機層に移行しないものとする。また、$10^{0.3} = 2$ とする。

1 4.0
2 5.0
3 6.0
4 7.0
5 9.0

問 97　日本薬局方に記載されているハロゲン化物の定性反応**A**～**D**に関する記述のうち、正しいのはどれか。**２つ**選べ。

定性反応

A　本品の溶液に［　ア　］試液を加えるとき、淡黄色の沈殿を生じる。沈殿を分取し、この一部に希硝酸を加えても溶けない。また、他の一部にアンモニア水（28）を加えて振り混ぜた後、分離した液に希硝酸を加えて酸性にすると$_{イ}$白濁する。

B　本品の溶液に［　ア　］試液を加えるとき、$_{ウ}$沈殿を生じる。この一部に希硝酸を、また、他の一部にアンモニア水（28）を追加してもいずれも沈殿は溶けない。

C　本品の溶液に［　ア　］試液を加えるとき、白色の沈殿を生じる。沈殿を分取し、この一部に希硝酸を加えても溶けない。また、他の一部に$_{エ}$過量のアンモニア試液を加えるとき、溶ける。

D　本品の溶液に塩素試液を加えるとき、黄褐色を呈する。これを二分し、この一部にクロロホルムを追加して振り混ぜるとき、クロロホルム層は黄褐色～赤褐色を呈する。また、他の一部にフェノールを追加するとき、$_{オ}$白色の沈殿を生じる。

1　［　ア　］に入る化合物は、塩化銀である。
2　下線部**イ**の白濁は臭化銀の生成による。
3　下線部**ウ**の沈殿は黒紫色を呈する。
4　下線部**エ**では銀イオンが水酸化物イオンと錯イオンを形成する。
5　下線部**オ**の白色の沈殿は 2,4,6-トリブロモフェノールである。

問 98　日本薬局方塩化カルシウム水和物（$CaCl_2$・$2H_2O$：147.01）の定量法に関する記述のうち、正しいのはどれか。**2つ**選べ。

本品約 0.4 g を精密に量り、水に溶かし、正確に 200 mL とする。この液 20 mL を正確に量り、水 40 mL 及び 8 mol/L［ア］2 mL を加え、更に NN 指示薬 0.1 g を加えた後、直ちに$_{イ}$0.02 mol/L エチレンジアミン四酢酸二水素二ナトリウム液で滴定する。ただし、滴定の終点は液の赤紫色が青色に変わるときとする。

0.02 mol/L エチレンジアミン四酢酸二水素二ナトリウム液 1 mL
＝［ウ］mg $CaCl_2$・$2H_2O$

1　［ア］に入れるべき溶液は、「アンモニア・塩化アンモニウム緩衝液」である。
2　下線部**イ**の溶液は遮光のガラス瓶に保存する。
3　［ウ］に入れるべき数値は、2.220 である。
4　Ca^{2+} とエチレンジアミン四酢酸との反応で生じたキレートの錯生成定数は、Ca^{2+} と NN 指示薬との反応で生じたキレートの錯生成定数より大きい。
5　本定量法では、試料溶液中に Mg^{2+} が共存していても、塩化カルシウム水和物を定量することができる。

問 99　固定相としてオクタデシルシリル（ODS）化シリカゲル、移動相としてアセトニトリルと水の混合液を用いて、ベンゼン、トルエン及びエチルベンゼンの分離を液体クロマトグラフィーにより行った。この分離に関する記述のうち、正しいのはどれか。**1つ**選べ。

1　エチルベンゼン、トルエン、ベンゼンの順で溶出する。

2　理論段高さの値が小さいカラムに変更することにより、各成分間の分離度が向上する。

3　移動相の流速と各成分間の分離係数は比例する。

4　移動相中のアセトニトリルの割合を大きくすることにより、各成分間の分離度が向上する。

5　固定相にシリカゲル、移動相に *n*-ヘキサン－アセトン混液を用いても、溶出順は変わらない。

問 100　キャピラリー電気泳動は、微量の試料の分析に極めて有用であり、臨床検査における血清タンパク質の分析にも用いられている。溶融シリカ毛細管を用いたキャピラリー電気泳動に関する記述のうち、正しいのはどれか。**2つ**選べ。

1　pH 7 の緩衝液を用いると、電気浸透流は陰極から陽極の方向に向かう。

2　キャピラリーゾーン電気泳動では pH 7 の緩衝液を用いると、陽イオン性物質と中性物質は同時に泳動される。

3　キャピラリーゲル電気泳動でタンパク質を分離すると、分子サイズの大きい順に検出される。

4　キャピラリー等電点電気泳動では、緩衝液に両性電解質（ポリアミノカルボン酸など）を溶解して分離を行う。

5　ミセル動電クロマトグラフィーでは、中性物質の相互分離が可能である。

問 101　D-リボースの構造を正しく表している Fischer 投影式はどれか。**1つ**選べ。

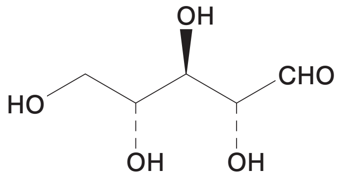

D-リボース

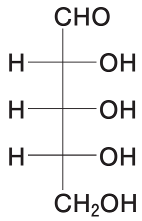

1

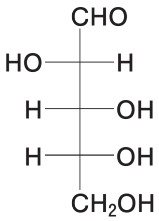

2

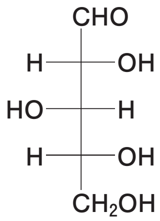

3

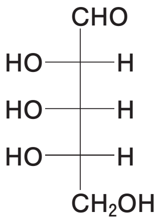

4

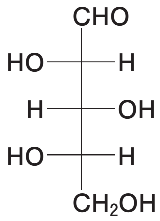

5

問 102　1,2-ジメチルシクロヘキサンの構造 **A** に関する記述のうち、正しいのはどれか。**2つ**選べ。

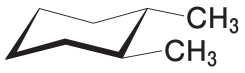

A

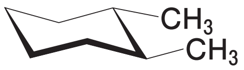

ア

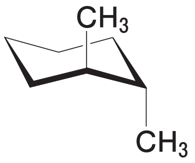

イ

1　**A** はシス体である。

2　**A** のエナンチオマーは**ア**である。

3　**A** のいす形配座が環反転した配座異性体は**イ**である。

4　**A** のメチル基は、両方ともエクアトリアル位に結合している。

5　**A** はメチル基同士に働く 1,3-ジアキシアル相互作用により不安定化されている。

問 103　主生成物としてメソ体を与えるのはどれか。**1つ**選べ。

1
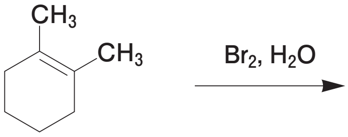

2
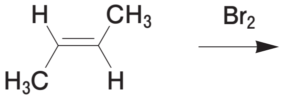

3
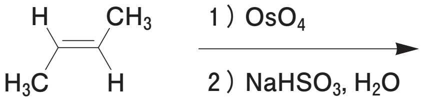

4
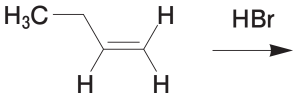

5
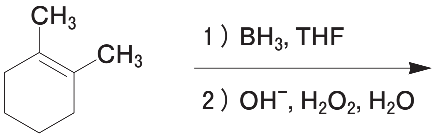

問 104　次の反応のうち、主生成物の構造を正しく示しているのはどれか。**1つ**選べ。ただし、各反応はそれぞれ適切な溶媒を用いて行い、反応終了後、適切な後処理を施したものとする。

1
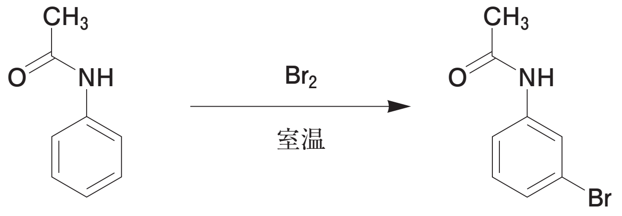

2
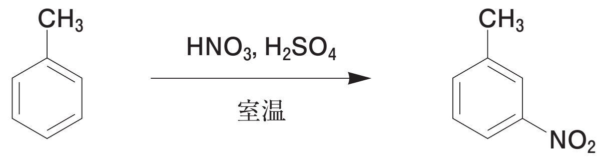

3
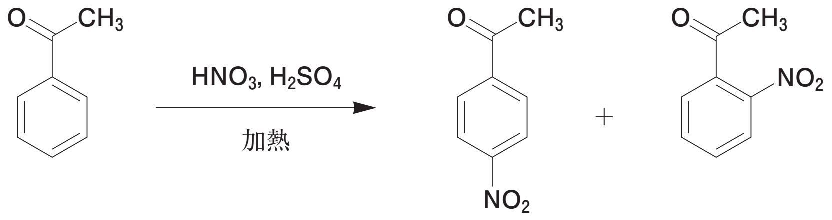

4
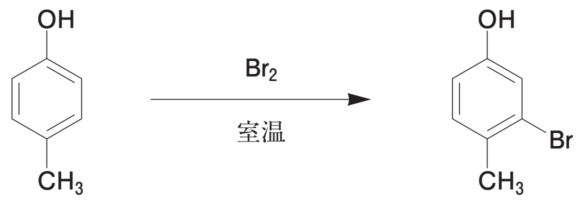

5
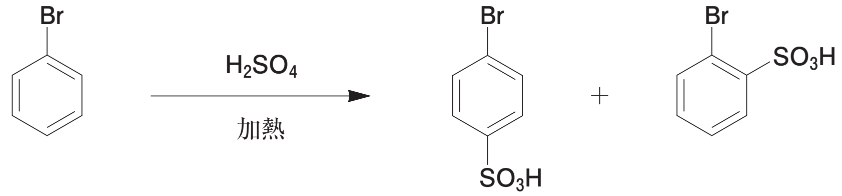

問 105　日本薬局方に収載されている次の確認試験に関する記述のうち、<u>誤っている</u>のはどれか。**1つ**選べ。

「本品 1 mL にヨウ素試液 2 mL 及び**A**試液 2 mL を加えて振り混ぜるとき、淡黄色の沈殿を生じる。」

1　ヨウ素は求電子剤として働く。
2　イソプロパノールはこの確認試験で陽性となる。
3　化合物の構造に含まれるホルミル基の検出に用いられる。
4　**A**は水酸化ナトリウムである。
5　淡黄色の沈殿はヨードホルムである。

問 106　ボルテゾミブは、プロテアソームの β5 サブユニットの N 末端トレオニン残基と結合し複合体を形成することにより、プロテアソームの働きを阻害する。以下の記述のうち、正しいのはどれか。**2つ**選べ。

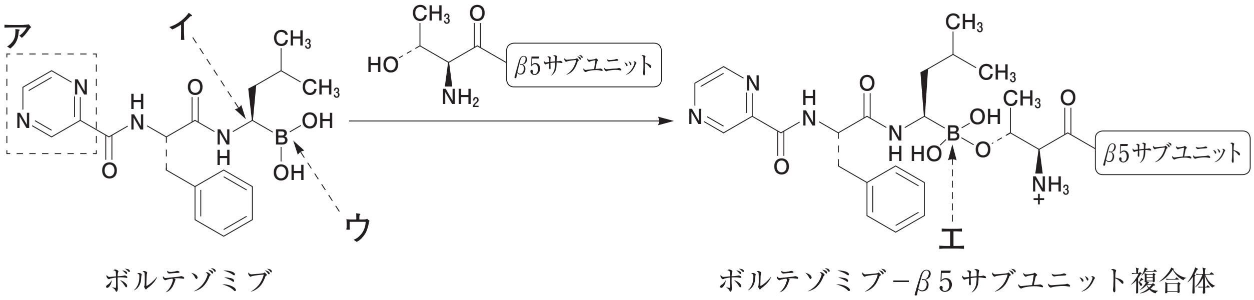

ボルテゾミブ　　　　ボルテゾミブ－β5 サブユニット複合体

1　**ア**で示した複素環はピリミジン環である。
2　**イ**で示した不斉中心の立体配置は S 配置である。
3　**ウ**で示したホウ素はルイス酸として働く。
4　**エ**で示したホウ素の形式電荷は +1 である。
5　**エ**で示したホウ素は sp^3 混成である。

問 107　次の抗悪性腫瘍薬のうち、DNA の塩基部分をアルキル化するのはどれか。**1つ**選べ。

1

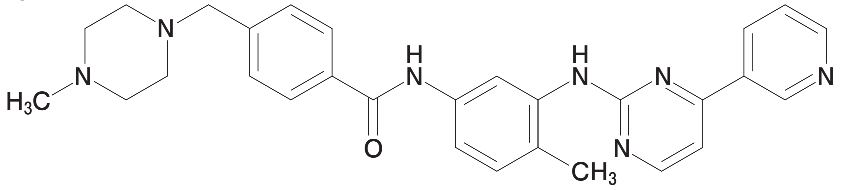

2

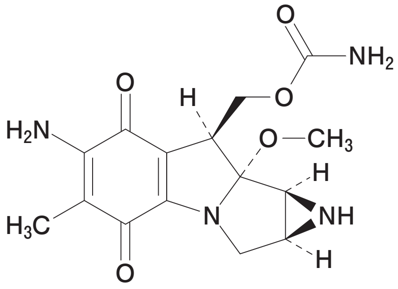

3

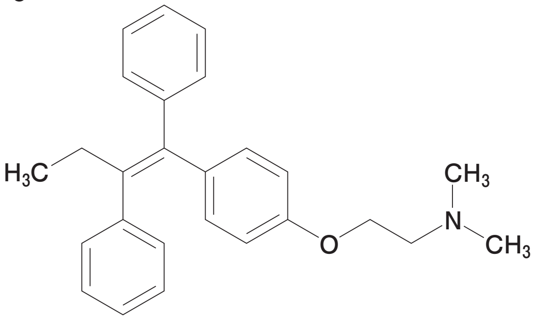

4

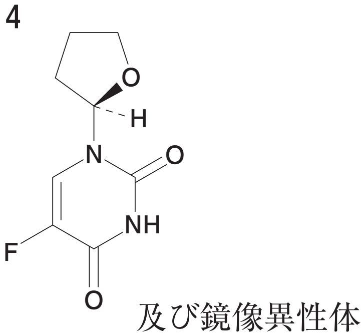

及び鏡像異性体

5

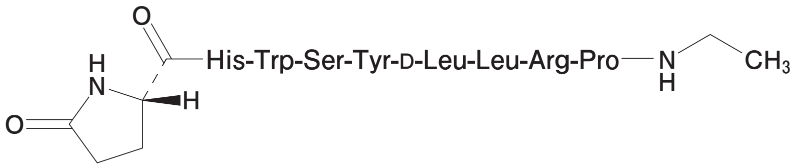

問 108　日本薬局方収載センナに関する記述のうち、誤っているのはどれか。**1つ**選べ。

1　マメ科植物 *Cassia angustifolia* Vahl または *Cassia acutifolia* Delile の小葉を基原とする生薬である。

2　確認試験としてマグネシウム－塩酸反応が用いられる。

3　純度試験として残留農薬（総 BHC 及び総 DDT）の量が規定されている。

4　瀉下作用を示す活性本体は、腸内細菌により生成したアントロン類である。

5　妊婦や妊娠している可能性のある女性に使用する場合には流早産の危険性があるため注意を要する。

問 109　天然物由来成分の化学構造を基に開発された、血液凝固抑制作用を有するクマリン誘導体はどれか。**1つ**選べ。

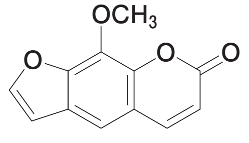

1

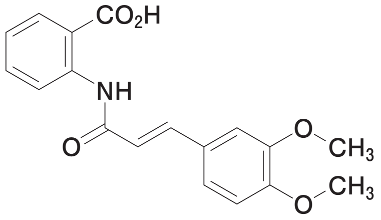

2

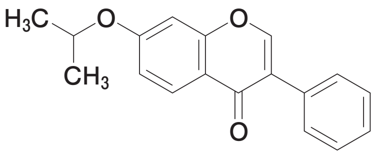

3

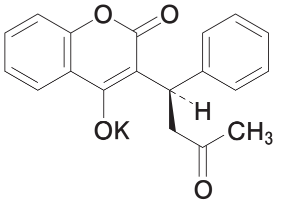

及び鏡像異性体

4

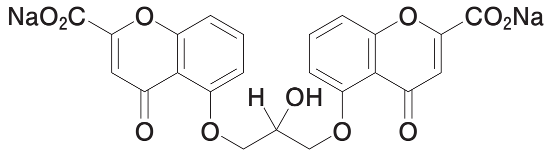

5

問 110　図は、洞房結節細胞の自発性活動電位に対する自律神経の影響を示したものである。以下の記述のうち、正しいのはどれか。<u>２つ</u>選べ。

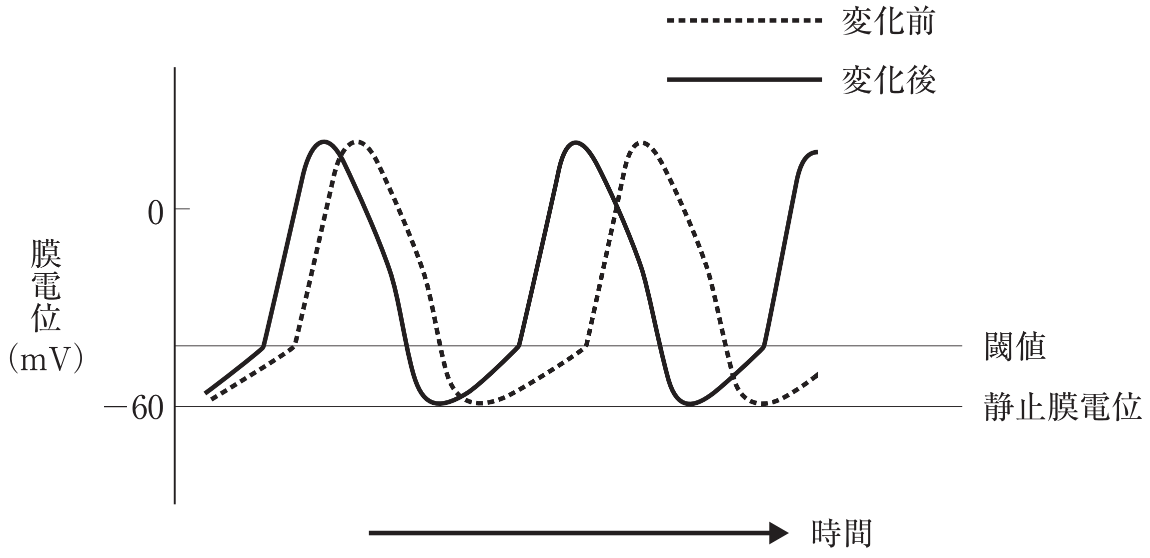

1　点線から実線への変化は、主にノルアドレナリンのアドレナリン β_1 受容体刺激によるものである。

2　点線から実線への変化は、主にアセチルコリンのアセチルコリン M_2 受容体刺激によるものである。

3　点線から実線への変化は、心拍数の減少を表している。

4　閾値からの急速な脱分極（第０相）は、主に細胞内への Ca^{2+} 流入によるものである。

5　閾値からの急速な脱分極（第０相）は、主に細胞外への Na^{+} 流出によるものである。

問 111 図は、アンジオテンシンⅡの生成経路とアンジオテンシンⅡによる血圧調節の概要を示したものである。この図の内容に関する記述のうち、正しいのはどれか。1つ選べ。

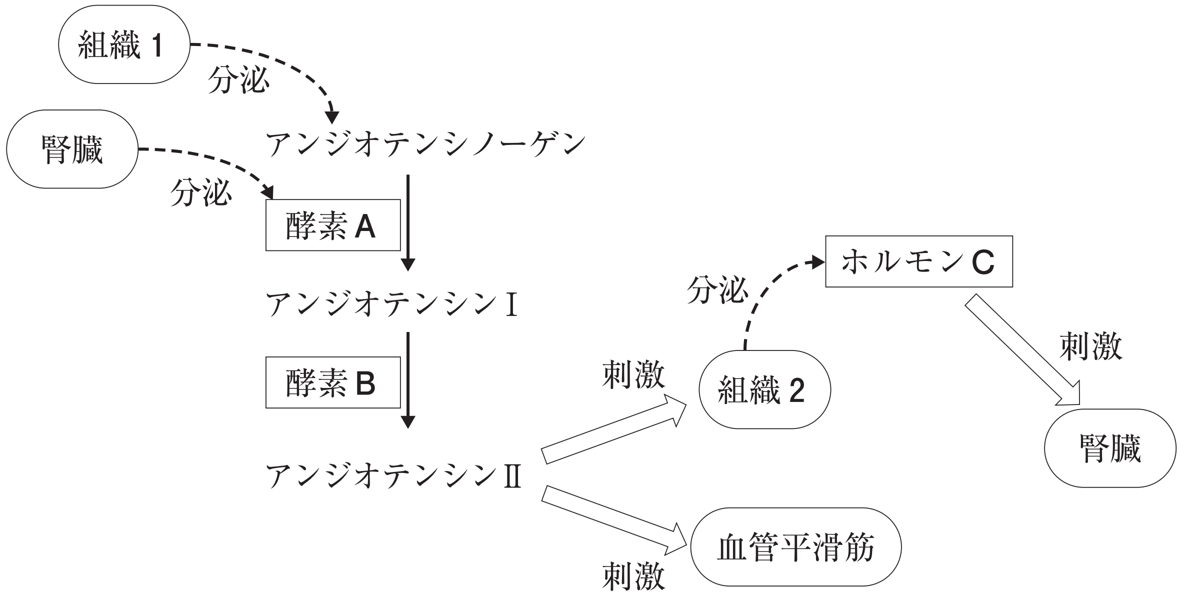

1 アンジオテンシノーゲンを分泌する組織 1 は、心臓である。

2 アンジオテンシノーゲンを限定分解する酵素 A は、腎臓の糸球体を流れる血液量が上昇した時に分泌量が増加する。

3 アンジオテンシンⅠを限定分解する酵素 B は、肺の毛細血管などに多く存在する。

4 アンジオテンシンⅡが作用する組織 2 は、副腎皮質網状層である。

5 ホルモン C は、腎臓の遠位尿細管での Na^+ の再吸収を抑える。

問 112　血小板に関わる分子についての記述のうち、正しいのはどれか。**2つ**選べ。

1　セロトニンは、血管の損傷部位で活性化された血小板から放出される。

2　セロトニンは、血小板のホスホジエステラーゼを活性化して、血小板凝集を抑制する。

3　アデノシン二リン酸（ADP）は、血小板内のイノシトール三リン酸（IP_3）量を増加させ、血小板凝集を促進する。

4　トロンボキサン A_2 は、血小板内の Ca^{2+} 濃度を上昇させ、血小板凝集を促進する。

5　プロスタグランジン I_2 は、血小板内のサイクリック AMP（cAMP）量を減少させ、血小板凝集を促進する。

問 113　糖新生に関する記述のうち、正しいのはどれか。**2つ**選べ。

1　乳酸、脂肪酸、ロイシン、グルタミン酸などからグルコースを生合成する代謝経路である。

2　主に骨格筋で起こる反応である。

3　律速酵素であるホスホエノールピルビン酸カルボキシキナーゼ（PEPCK）は、ピルビン酸からホスホエノールピルビン酸を生成する。

4　糖新生の中間体であるホスホエノールピルビン酸の生成には、GTP が必要である。

5　グルカゴン刺激により、*PEPCK* 遺伝子の発現が亢進する。

問 114　薬物Aに感受性のある培養細胞を用いて、その細胞内の代謝調節タンパク質Bについて調べることにした。

図1

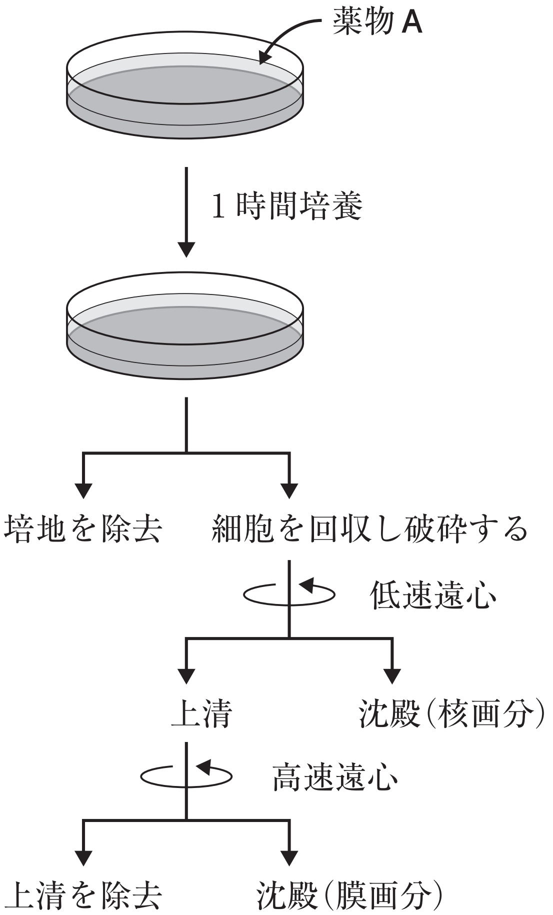

操作の流れを図1に示す。培地に薬物Aを添加して細胞を1時間培養した後、培地を除去してから細胞を回収した。細胞を破砕し、低速度の遠心操作で核画分を分離回収した。さらに高速度の遠心操作で、核を除いた細胞の膜画分を分離回収した。また、対照として、培地に薬物Aを添加しなかった細胞についても同様の操作を行った。

ドデシル硫酸ナトリウムを用いたポリアクリルアミド電気泳動（SDS-PAGE）にて、核画分及び膜画分中のタンパク質を分離し、タンパク質Bに対するポリクローナル抗体を用いてウエスタンブロットを行った。SDS-PAGEは、還元剤（2-メルカプトエタノール）を添加した条件と添加しない条件の2通りの方法で行ったところ、図2に示す結果を得た。

図 2

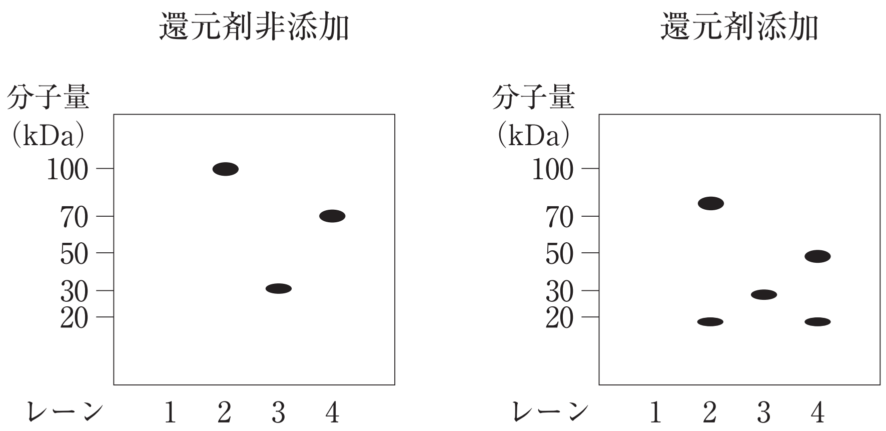

レーン 1　薬物 A 非添加　核画分
2　薬物 A 非添加　膜画分
3　薬物 A 添加　核画分
4　薬物 A 添加　膜画分

以上の実験とその結果から推測される記述のうち、正しいのはどれか。__2つ__選べ。

1　タンパク質 B は、核膜に局在するタンパク質である。

2　タンパク質 B は、分子間ジスルフィド結合を持つ。

3　タンパク質 B から生じた約 30 kDa のタンパク質は、核内に移行する。

4　タンパク質 B は、薬物 A で刺激された細胞内で 3 つに切断される。

5　タンパク質 B の分子量は、約 50 kDa である。

問 115　テロメアに関する記述のうち、正しいのはどれか。2つ選べ。

1　テロメアは、染色体の末端に存在し、特定の DNA 塩基配列の繰り返し構造を含む。

2　テロメアを開始点として DNA が複製される。

3　生殖細胞系列の分裂時には、テロメアは短縮する。

4　多くのがん細胞で、テロメアを伸長させるテロメラーゼが発現している。

5　テロメア伸長の鋳型として、tRNA が利用される。

問 116　図は、単量体で作用する酵素のヒト遺伝子構造を示したものである。5つのエキソンを順に数字で表し、矢印A、Bは、頻度が高い2種類の遺伝子多型A、Bのそれぞれの位置を示す。多型Aはプロモーター領域に、多型Bは翻訳領域に存在する。この遺伝子は常染色体上に存在し、多型Aのヘテロ接合体では、野生型ホモ接合体と比べて、この酵素の活性がほぼ半分になる。多型Bのヘテロ接合体では、酵素活性が野生型ホモ接合体の約 3/4 になる。

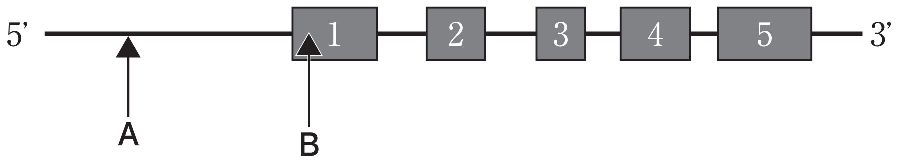

この多型に関する記述のうち、正しいのはどれか。2つ選べ。

1　多型Aは、この酵素の mRNA 量に影響する。

2　多型Bは、サイレント変異である。

3　多型Aヘテロ接合体と多型Bヘテロ接合体の夫婦からは、多型Aと多型Bを両方もつ子が生まれる可能性がある。

4　多型Bの遺伝子産物のアミノ酸配列は、野生型と同一である。

5　多型Bは、逆転写酵素を用い、第2エクソン内の配列に対する RT-PCR で判定できる。

問 117　ヒトの免疫系の組織と細胞に関する記述のうち、正しいのはどれか。**２つ**選べ。

1　骨髄では、造血幹細胞が分裂している。

2　胸腺では、B 細胞が正の選択と負の選択を受け、形質細胞へと分化する。

3　リンパ節では、高内皮細静脈から移行した T 細胞が、樹状細胞に対して抗原提示をする。

4　肝臓では、老化した赤血球が除去される一方で、血液中の抗原に対する免疫応答が行われる。

5　小腸のパイエル板では、上皮層の M 細胞を介して取り込まれた抗原に対する免疫応答が行われる。

問 118　図は、ヒト免疫グロブリン G（IgG）の構造を模式的に示したものである。領域 A ～領域 E で示した IgG の部分構造に関する記述のうち、正しいのはどれか。**２つ**選べ。

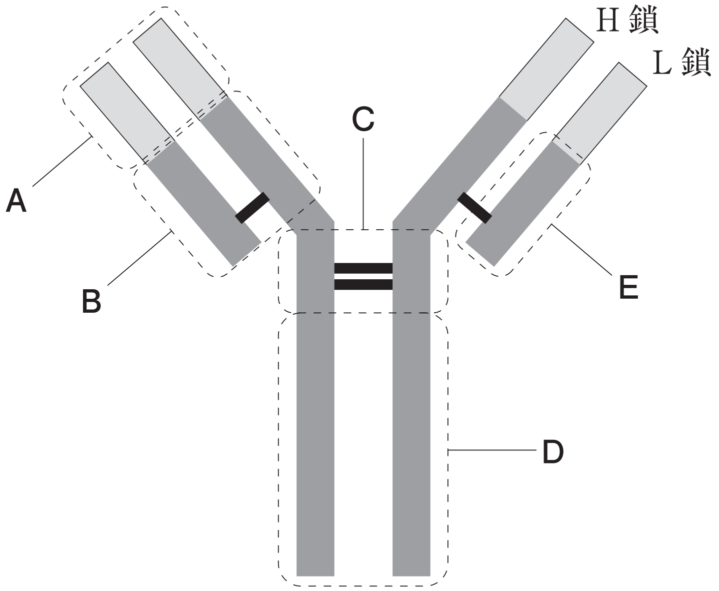

1　領域 A は、個体ごとに決められた一定のアミノ酸配列を示す。

2　領域 B で、*N*-結合型の糖鎖修飾がなされている。

3　領域 C では、２本の H 鎖がシステイン残基間で共有結合している。

4　領域 D は、マクロファージの細胞膜上の受容体に結合する。

5　領域 E により、IgG のサブクラスが決定される。

問 119　細菌の細胞表面構造に関する記述のうち、正しいのはどれか。２つ選べ。

1　グラム陽性細菌には、タイコ酸やリポタイコ酸が結合した厚いペプチドグリカン層が、細胞膜の内側に存在する。

2　グラム陰性細菌には、細胞膜（内膜）の外側に薄いペプチドグリカン層があり、さらにその外側には内毒素であるリポ多糖を含む外膜が存在する。

3　淋菌などナイセリア属菌には、ミコール酸と呼ばれる長鎖脂肪酸を多量に含んだ厚い脂質層が存在する。

4　マイコプラズマには、ペプチドグリカンや細胞膜が存在しない。

5　肺炎球菌には、多糖を主成分とする莢膜が存在する。

一般問題（薬学理論問題）【衛生】

問 120　下表は、2020 年 10 月 1 日現在の年齢区分別人口割合を示したものである。この表に基づく人口指標に関する記述のうち、正しいのはどれか。**２つ**選べ。

年齢区分	人口割合（%）
15 歳未満	12
15～64 歳	59
65～74 歳	14
75 歳以上	15

1　老年化指数は 250 を超えている。
2　老年人口指数は 50 を超えている。
3　年少人口指数は 20 を超えている。
4　従属人口指数は 80 を超えている。
5　老年人口割合は 25%を超えている。

問 121　疫学調査の結果に基づいて因果関係を判定する際の基準に関する記述のうち、正しいのはどれか。**２つ**選べ。

1　関連の一致性とは、対象とする要因と疾病に関して、調査の時期、場所、対象集団などを変えても同様の結果が得られることである。
2　関連の強固性とは、疫学以外の実験的な研究で得られた知見や理論と疫学調査の結果が矛盾しないことである。
3　関連の特異性とは、要因の曝露があると相対危険度やオッズ比が統計学的に有意に高くなることである。
4　関連の時間性とは、疾病の発生以前に要因の曝露があることである。
5　関連の整合性とは、要因の曝露があれば必ず疾病の発生があり、曝露がなければ疾病の発生がないことである。

問 122　検疫に関する記述のうち、誤っているのはどれか。1 つ選べ。

1　検疫法は、国内に常在しない感染症の病原体が船舶又は航空機を介して国内に侵入することの防止を目的としている。

2　検疫感染症の患者は、入国停止、隔離、停留あるいは消毒等の措置がとられる。

3　新興感染症は、すべて検疫感染症に含まれる。

4　検疫感染症には、感染症法*に定める一類感染症が含まれる。

5　検疫感染症には、感染症法*に定める新型インフルエンザ等感染症が含まれる。

*感染症法：感染症の予防及び感染症の患者に対する医療に関する法律

問 123　性感染症に関する記述のうち、誤っているのはどれか。1 つ選べ。

1　コンドームの使用や不特定多数との性交渉を避けることによって、感染リスクを低減することができる。

2　梅毒は、感染症法*で五類感染症に分類され、全数把握が必要である。

3　我が国における後天性免疫不全症候群の患者数は、異性間よりも同性間の性的接触によるものが多い。

4　尖圭コンジローマは、ヒト単純ヘルペスウイルスを原因とする。

5　性器クラミジア感染症は、母子感染により発症することがある。

*感染症法：感染症の予防及び感染症の患者に対する医療に関する法律

問 124 図は、我が国のリスク要因別の関連死亡者数を示したものである。リスク要因 **a**～**c** の組合せとして、正しいのはどれか。**1つ**選べ。

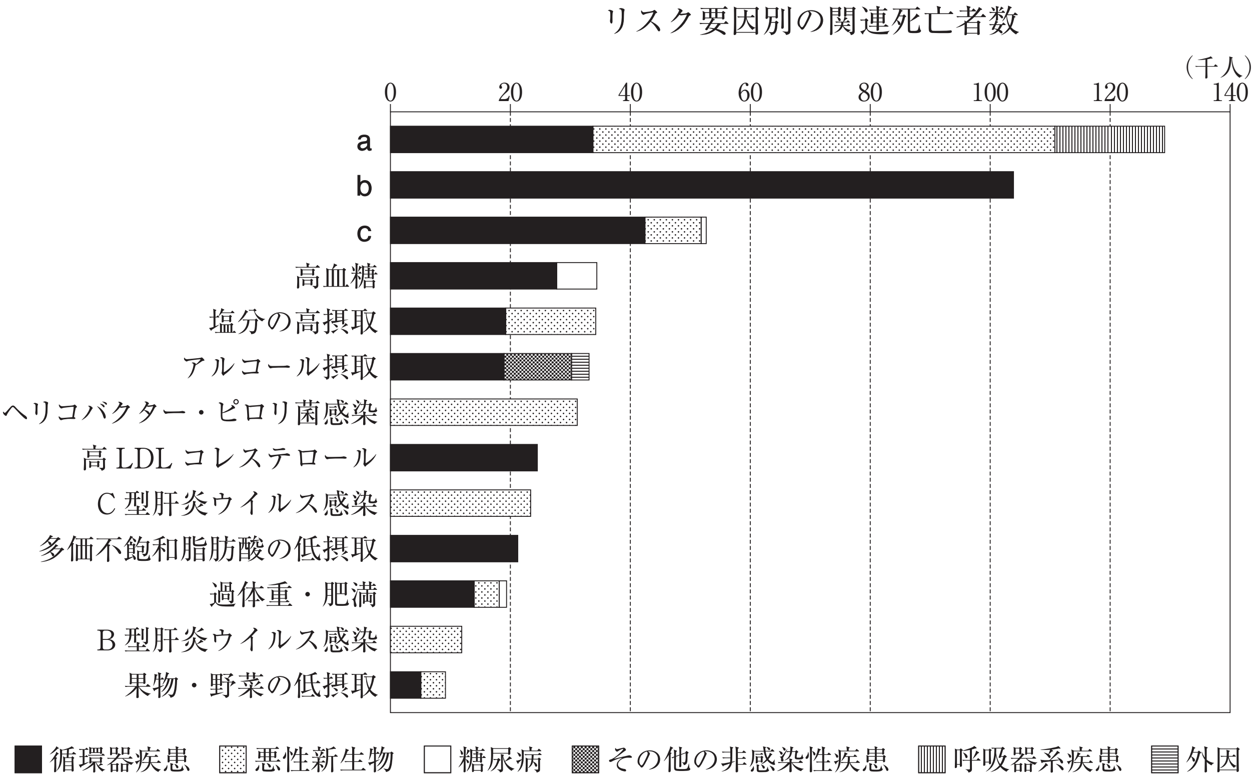

令和 2 年版　厚生労働白書より引用

	a	b	c
1	運動不足	高血圧	喫煙
2	喫煙	運動不足	高血圧
3	喫煙	高血圧	運動不足
4	高血圧	喫煙	運動不足
5	高血圧	運動不足	喫煙

問 125　図は、1950 年代から 2010 年代における心疾患及び脳血管疾患の死亡率の年次推移を示したものである。疾患**ア**〜**エ**は、心不全、虚血性心疾患、脳梗塞、脳内出血のいずれかである。次の記述のうち、<u>誤っている</u>のはどれか。**1つ**選べ。

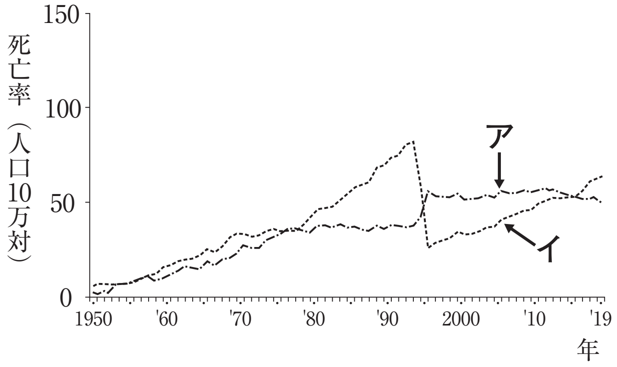

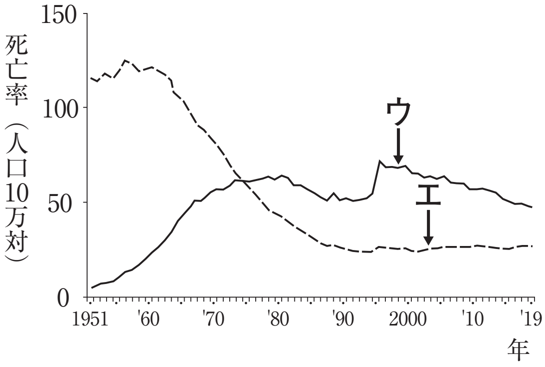

（注）1994 年及び 1995 年の大きな変化は、死亡診断書の注意書きの周知あるいは国際ルール（ICD-10）適用の影響による。
国民衛生の動向 2020/2021 より引用

1　疾患**ア**による死亡率には、狭心症や心筋梗塞による死亡が含まれる。

2　1995 年以降の疾患**イ**の死亡率の上昇には、老年人口の割合の増加が関係している。

3　疾患**ウ**は、脳内出血である。

4　1960 年以降、疾患**エ**の死亡率が低下した原因として、食塩摂取量の低下やタンパク質摂取量の増加がある。

5　寒冷刺激は、疾患**エ**のリスクファクターとなる。

問 126　職業性疾病と有害要因に関する記述のうち、誤っているのはどれか。**1つ**選べ。

1　精神障害やメンタルヘルス不調は、強い不安やストレスを感じる心理社会的要因により発症する。

2　レイノー病は、電離放射線の曝露による物理的要因により発症する。

3　けい肺や石綿肺は、粉じんの曝露による化学的要因により発症する。

4　細菌感染症やウイルス感染症は、病原体との接触による生物的要因により発症する。

5　頸肩腕症候群は、VDT（visual display terminal）作業による作業態様要因により発症する。

問 127　食物繊維に関する記述のうち、正しいのはどれか。**2つ**選べ。

1　水溶性のものと不溶性のものがある。

2　ヒトの消化酵素で消化されず、腸内細菌によっても分解されない。

3　セルロースとリグニンは、どちらも多糖である。

4　天然の食物繊維は植物性食品由来であり、動物性食品由来のものはない。

5「日本人の食事摂取基準（2020 年版）」において、食物繊維には目標量が設定されている。

問 128　未使用のコーン油とオリーブ油について、油脂の変質に対する温度の影響を調べる実験を行った。実験では、60 ℃の一定温度で 7 週間保存し、1 週間ごとに過酸化物価（meq/kg）と酸価（mg/g）の測定を行った。結果は以下のグラフに示すとおりである。なお、実験に用いたコーン油とオリーブ油の実験開始前（開封直後）におけるヨウ素価（g/100 g）は、コーン油が 124、オリーブ油が 75 であった。コーン油はリノール酸を、オリーブ油はオレイン酸を最も多く含む。コーン油とオリーブ油の変質試験に関する記述のうち、正しいのはどれか。__２つ__選べ。

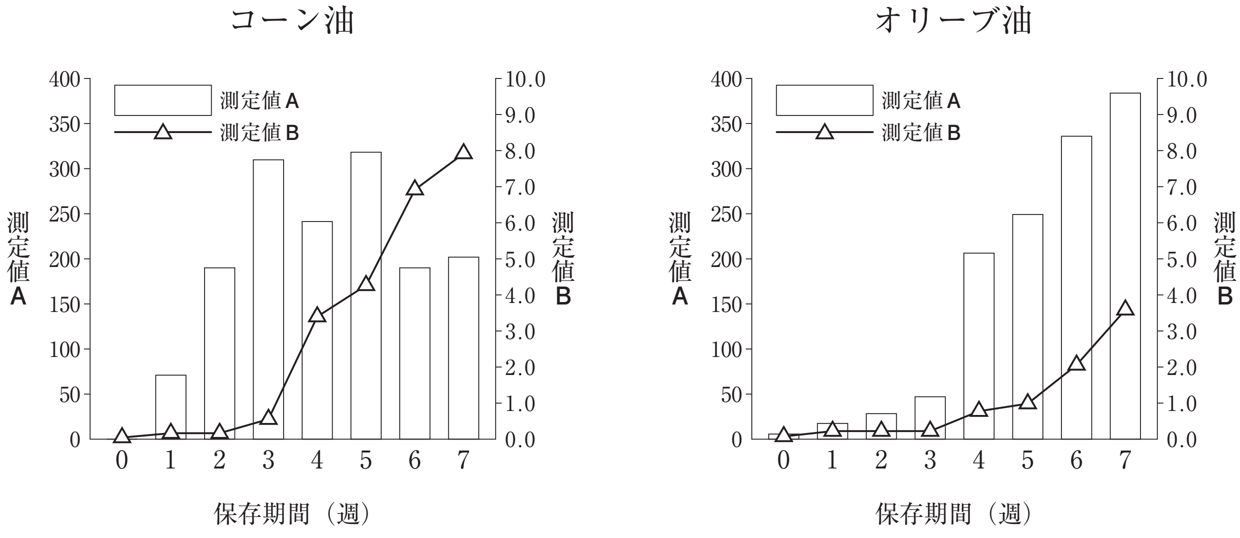

1　測定値Aは過酸化物価、測定値Bは酸価である。

2　コーン油は、オリーブ油よりも開封直後のヨウ素価が大きいことから、飽和脂肪酸を多く含むことがわかる。

3　コーン油は、オリーブ油よりも早い時期に測定値Aの値が上昇していることから、オリーブ油よりも酸化しやすいことがわかる。

4　コーン油とオリーブ油は、いずれも測定値Bが 4 週目から上昇していることから、酸化のされやすさは同じであることがわかる。

5　未使用のコーン油とオリーブ油を低温・暗所で保存した場合では、測定値Aと測定値Bの値の上昇の程度は、この実験結果よりも増加すると予想される。

問 129　食品成分の変化に関する記述のうち、正しいのはどれか。２つ選べ。

1　メイラード反応とは、還元糖とアミノ酸が酵素的に反応し、シッフ塩基及び α-カルボニル化合物を形成する反応である。

2　チラミンは、アミノ酸脱炭酸酵素によりチロシンから生成され血圧上昇作用を示す。

3　魚の腐敗臭の原因となるトリメチルアミンは、トリメチルアミン N-オキシドが酸化されることにより生じる。

4　トリプトファンは、脱アミノ反応及び脱炭酸反応によって腐敗臭を有する硫化水素を生じる。

5　糖質が微生物により分解されて、アルコールや有機酸などの有用な化合物が生成することを発酵という。

問 130　食品の加熱により、アミノ酸が関与する反応で生じる発がん物質はどれか。
２つ選べ。

1

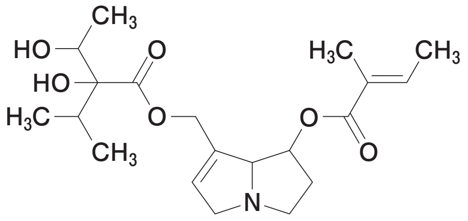

2

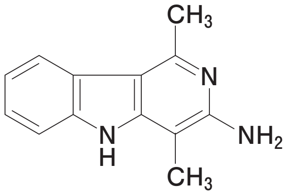

3

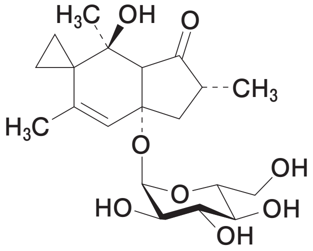

4

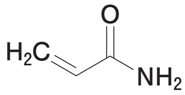

5

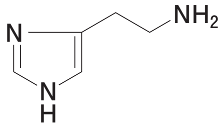

問 131　活性酸素に対する生体内の防御因子に関する記述のうち、正しいのはどれか。**２つ**選べ。

1　スーパーオキシドジスムターゼ（SOD）は、スーパーオキシドアニオンを水に変換する。

2　銅（Cu）と亜鉛（Zn）を含む Cu/Zn-SOD は、ミトコンドリアに局在する。

3　カタラーゼは、過酸化水素を酸素と水に変換する酵素で、活性中心にヘム鉄をもつ。

4　グルタチオンペルオキシダーゼは、グルタチオン存在下で過酸化水素を水に還元する反応を触媒する。

5　グルタチオンレダクターゼは、スーパーオキシドアニオンを過酸化水素と酸素に変換する。

問 132　農薬に関する記述のうち、正しいのはどれか。**２つ**選べ。

1　フェニトロチオンは、シトクロム P450 による酸化的脱硫反応により代謝的活性化を受けてアセチルコリンエステラーゼを阻害する。

2　メソミルは、アセチルコリンエステラーゼの活性中心を可逆的にカルバモイル化する。

3　パラコートは、神経の電位依存性 Na^+ チャネルに作用する。

4　アセタミプリドは、１電子還元されてラジカルを生成し、スーパーオキシドアニオンを生じる。

5　フェノトリンは、ニコチン性アセチルコリン受容体に結合し、神経を興奮させる。

一般問題（薬学理論問題）【物理・化学・生物／衛生／法規・制度・倫理】

問 133-135　自宅の寝室で倒れている男性が救急搬送された。簡易検査の結果をもとに、直ちに対応する解毒薬が投与された。一方、寝室にはコップの中に大量の錠剤が沈んだ飲料水が残されていた。

問 133（物理・化学・生物）

錠剤の成分が何かを調べるため、コップの中に残された薬剤に適切な処理をしたのち、^{1}H NMR スペクトル（$CDCl_3$ 溶媒中）を測定した。得られたスペクトルをデータベースと照合したところ、図に示したチャートとシグナル及び積分値が一致した。錠剤の成分として推定される医薬品 **A** の構造はどれか。**1つ**選べ。ただし、図は TMS を基準（0 ppm）とし、シグナルを積分曲線と共に示したもので、×は重溶媒中に微量に含まれる $CHCl_3$ のシグナルである。

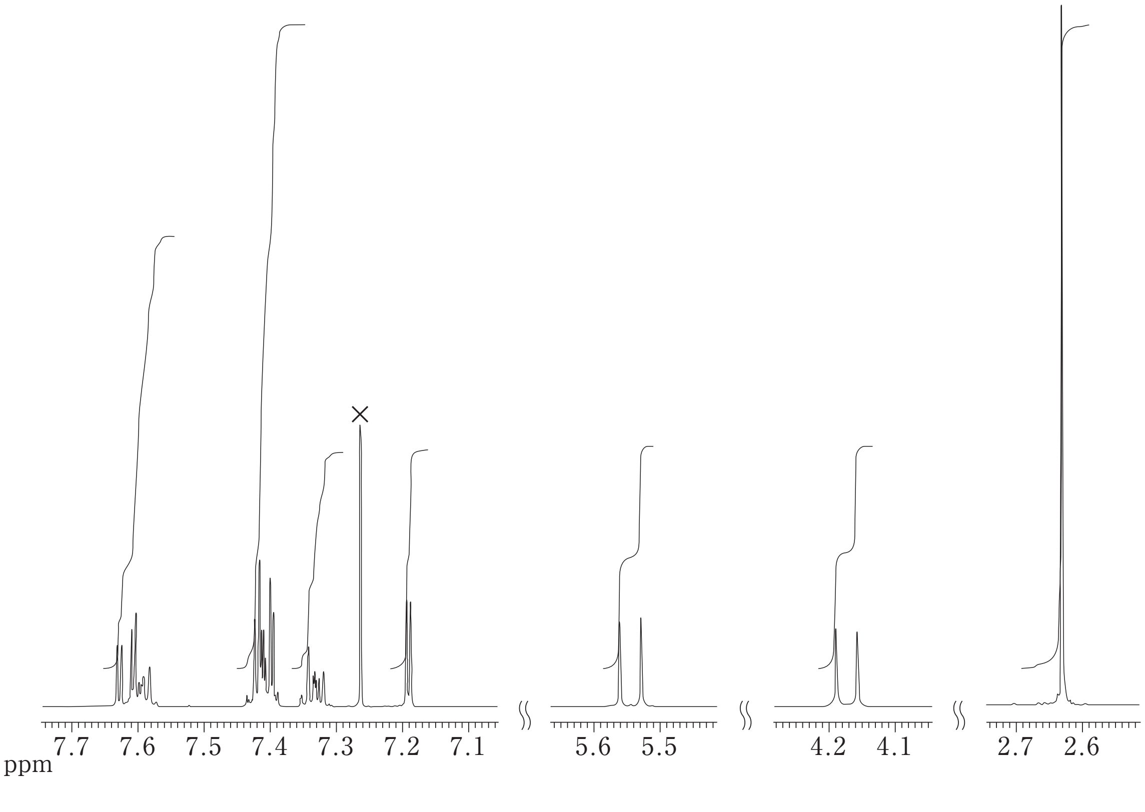

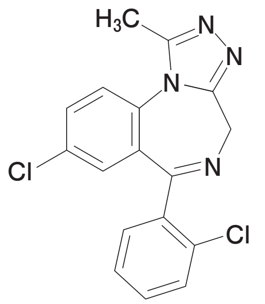

1

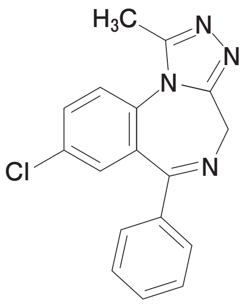

2

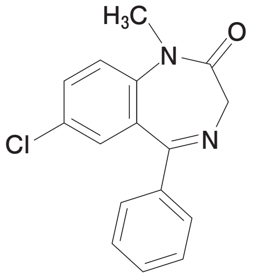

3

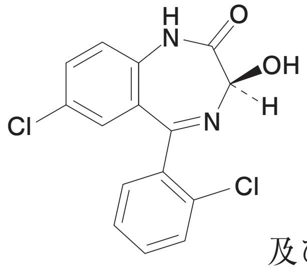

及び鏡像異性体

4

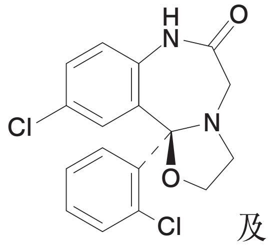

及び鏡像異性体

5

問 134（法規・制度・倫理）

法令上、この医薬品Aが該当するのはどれか。1つ選べ。

1 麻薬
2 向精神薬
3 覚醒剤
4 指定薬物
5 要指導医薬品

問 135（衛生）

この患者に投与された解毒薬として、適切なのはどれか。1つ選べ。

1 ネオスチグミン
2 ナロキソン
3 フルマゼニル
4 ヨウ化プラリドキシム
5 ジメルカプロール

問 136　ある食品汚染物質の耐容一日摂取量（TDI）は、下図に示した動物試験の結果から得られた無毒性量（NOAEL）に、不確実係数100を適用して定められている。この食品汚染物質のTDI（μg/kg体重/日）として、適切な値はどれか。**1つ**選べ。

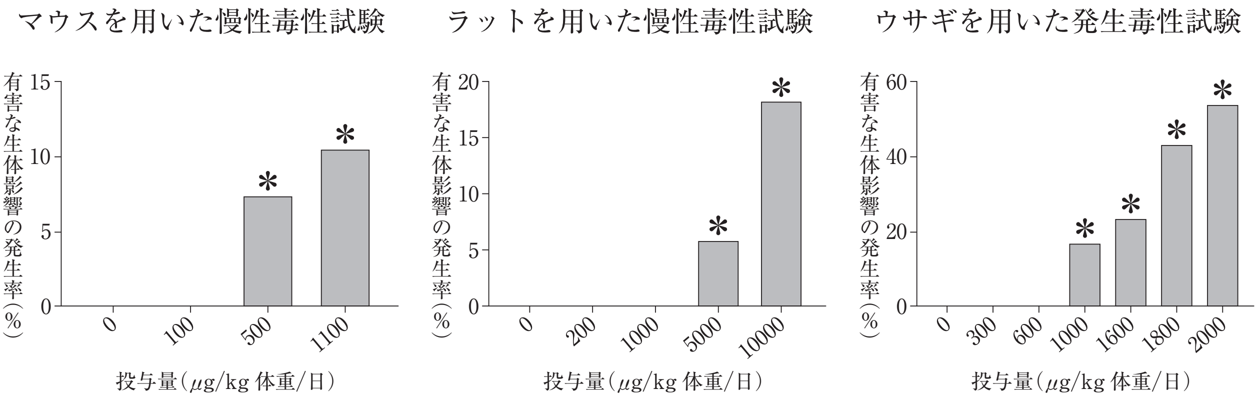

＊は、それぞれの対照群（投与量0μg/kg体重/日）と比較して、統計的に有意な影響であることを示している。

1　1
2　3
3　5
4　6
5　10

問 137　*In vitro* 遺伝毒性試験に関する記述のうち、正しいのはどれか。**２つ**選べ。

1　不定期 DNA 合成（UDS）試験は、哺乳類細胞を用いて化学物質による突然変異を評価する方法である。

2　マウスリンフォーマ TK 試験は、哺乳類細胞を用いて化学物質による生殖細胞遺伝毒性を評価する方法である。

3　Ames 試験は、細菌を用いて化学物質による復帰突然変異を評価する方法である。

4　コメットアッセイは、哺乳類細胞を用いて化学物質による DNA 鎖の切断を評価する方法である。

5　小核試験は、細菌を用いて化学物質の染色体異常誘発性を評価する方法である。

問 138　天然及び人工放射性核種に関する記述のうち、正しいのはどれか。**２つ**選べ。

1　食品から摂取される天然放射性核種の中で、最も量が多いのは ^{40}K である。

2　^{40}K は、α 線を放出して崩壊する。

3　体内に取り込まれた人工放射性核種 ^{137}Cs は、生体内で筋肉（全身）に集積する。

4　物理学的半減期が 28.8 年である ^{90}Sr の生物学的半減期を 50 年とすると、実効半減期は 31.2 年となる。

5　^{226}Ra などの天然放射性核種から放出される α 線は、β^{-} 線及び γ 線と比較して、体内被曝による生体損傷が小さい。

問 139　上水道における浄水処理に関する記述のうち、正しいのはどれか。**２つ**選べ。

1　凝集沈殿とろ過処理の間に行う塩素処理を前塩素処理という。

2　水中のアンモニウムイオンは、塩素消費量増加の原因となる。

3　不連続点塩素処理の目的は、トリハロメタンの生成を抑制することである。

4　オゾンによる高度浄水処理では、臭気物質などが酸化分解により除去される。

5　水中のアルカリ分と硫酸アルミニウムが反応して、水酸化アルミニウムゲルが生成し、沈降性のフロックが形成される。

問 140　大気中の窒素酸化物に関する記述のうち、正しいのはどれか。2つ選べ。

1　大気中の窒素酸化物は水分と反応して、酸性雨の原因となる。

2　サーマル NOx は、化石燃料中の窒素化合物の燃焼に由来する。

3　大気中の窒素酸化物は、非メタン炭化水素と反応して、光化学オキシダントの原因となる。

4　大気中へ排出される窒素酸化物は、大気汚染防止法により施設単位の排出基準に基づく規制（*K* 値規制）が行われている。

5　2010 年度以降における二酸化窒素の大気環境基準の達成率は、一般環境大気測定局（一般局）、自動車排出ガス測定局（自排局）のいずれにおいても約 80%で推移している。

問 141　室内環境と健康に関する記述のうち、正しいのはどれか。2つ選べ。

1　ヒョウダニの死骸や排泄物は、気管支ぜん息や鼻炎などのアレルギー性疾患の原因となる。

2　暖房器具の不完全燃焼で生成する一酸化炭素は、一酸化窒素よりもヘモグロビンに対する親和性が高い。

3　レジオネラ属菌が混入したエアロゾルを吸入すると、日和見感染症として肺炎を引き起こすことがある。

4　総揮発性有機化合物（TVOC）の暫定目標値は、室内空気汚染物質の毒性を基に定められている。

5　化学物質の室内濃度指針値は、それぞれの化学物質がシックハウス症候群を引き起こす閾値に不確実係数を適用して定められている。

一般問題（薬学理論問題）【法規・制度・倫理】

問 142 薬剤師法に規定されている薬剤師の業務に関する記述のうち、正しいのはどれか。<u>２つ</u>選べ。

1 調剤に従事する薬剤師は、調剤の求めがあった場合には、正当な理由がなければ、調剤を断ってはならない。

2 処方箋中の疑わしい点について、処方医と連絡がとれない場合には、照会せずに調剤することができる。

3 薬局以外の場所では、いかなる場合も、販売又は授与の目的で調剤することはできない。

4 医師などの処方箋によらなければ、販売又は授与の目的で調剤することはできない。

5 調剤に従事する薬剤師は、薬剤師免許を携帯しなければ調剤できない。

問 143 医薬品の開発における臨床試験に関する記述のうち、正しいのはどれか。**1つ**選べ。

1 第Ⅰ相試験の主な目的は、用法・用量を決定することである。

2 前期第Ⅱ相試験の主な目的は、薬物動態試験の実施である。

3 後期第Ⅱ相試験の主な目的は、臨床薬理試験の実施である。

4 第Ⅲ相試験の主な目的は、検証的試験の実施である。

5 第Ⅳ相試験の主な目的は、効能・効果を追加することである。

問 144　医薬品リスク管理計画に関する説明のうち、正しいのはどれか。2つ選べ。

1　GCP 省令に基づき、医薬品の製造販売後のリスクとベネフィットを評価する。
2　安全性検討事項として、重要なリスクを特定し、それに対して安全性監視計画とリスク最小化計画を策定・実施する。
3　安全性検討事項には、特定されたリスクに加え、潜在的なものや不足情報も含まれる。
4　安全性監視計画には、添付文書の作成や改訂が含まれる。
5　リスク最小化計画には、副作用・感染症報告制度に基づく副作用評価が含まれる。

問 145　次の分類のうち、コンタクトレンズが該当するのはどれか。1つ選べ。

1　高度管理医療機器
2　管理医療機器
3　一般医療機器
4　再生医療等製品
5　医薬部外品

問 146　医薬品である覚醒剤原料について、薬局における法令に基づく取扱いとして、正しいのはどれか。2つ選べ。

1　かぎをかけて薬品庫に保管する。
2　麻薬と一緒に保管できる。
3　薬局で調剤するためには、覚醒剤施用機関としての指定を受ける必要がある。
4　処方箋に基づき調剤し、患者に譲渡することができる。
5　使用期限が切れた調剤前のものを廃棄した場合、30 日以内に都道府県知事に届け出る。

問 147　個人情報の保護に関する法律（個人情報保護法）に関する記述のうち、正しいのはどれか。２つ選べ。

1　個人情報の取り扱いが 5,000 件未満の薬局は、個人情報取扱事業者に該当しない。

2　健康保険法に基づく保険者番号及び被保険者等記号・番号は、個人情報に該当しない。

3「匿名加工情報」とは、本人の人種、信条、社会的身分、病歴等の特に配慮を要する個人情報をいう。

4　個人情報取扱事業者は、偽りその他不正の手段により個人情報を取得してはならない。

5　個人情報取扱事業者は、原則、あらかじめ本人の同意を得ないで、個人データを第三者に提供してはならない。

問 148　医療法に関する記述のうち、正しいのはどれか。２つ選べ。

1　診療所は、専任薬剤師を置かなければならない。

2　病院は、20 人以上の患者を入院させるための施設を有する。

3　地域医療支援病院の承認要件には、救急医療を提供する能力が含まれる。

4　特定機能病院の承認要件には、特定臨床研究に関する計画を立案し、実施する能力が含まれる。

5　臨床研究中核病院の承認要件には、高度の医療を提供する能力が含まれる。

問 149　介護保険制度に関する記述のうち、正しいのはどれか。**2つ**選べ。

1　高齢者の介護を社会全体で支え合う仕組みとして導入された。

2　財源は税金と 40 歳以上の国民が負担する保険料である。

3　薬剤師は、実務経験無しで、介護支援専門員の資格を取得できる。

4　ケアプランは、かかりつけの医師が作成する。

5　介護サービスを受けるために支給される金額は、年齢によって限度額が決まっている。

問 150　新規感染症予防のために新しいワクチンが開発された。臨床試験ではワクチン接種群では 2 万人のうち 10 人が発症、プラセボ接種群では 2 万人のうち 200 人が発症し、ワクチンの有効率は 95% であった。この新しいワクチンの接種費用は 1 人当たり 1 万円で、その他の費用は考慮しない場合、このワクチンの増分費用効果比（1 人の発症を防ぐための費用）として、最も近い値はどれか。**1つ**選べ。

1　1 万円/人

2　10 万円/人

3　20 万円/人

4　100 万円/人

5　200 万円/人

107　１日目③　一般問題（薬学理論問題）

【薬理、薬剤、病態・薬物治療】

◎指示があるまで開いてはいけません。

注　意　事　項

1　試験問題の数は、**問１５１**から**問１９５**までの４５問。
　１５時５０分から**１７時４５分**までの**１１５分以内**で解答すること。

2　解答方法は次のとおりである。

(1)　一般問題（薬学理論問題）の各問題の正答数は、**問題文中に指示されている**。
　問題の選択肢の中から答えを選び、次の例にならって答案用紙に記入すること。
　なお、問題文中に指示された正答数と**異なる数を解答すると、誤りになる**から注意すること。

（例）**問 500**　次の物質中、常温かつ常圧下で液体のものはどれか。**２つ**選べ。

1　塩化ナトリウム　　2　プロパン　　3　ベンゼン
4　エタノール　　5　炭酸カルシウム

正しい答えは「3」と「4」であるから、答案用紙の

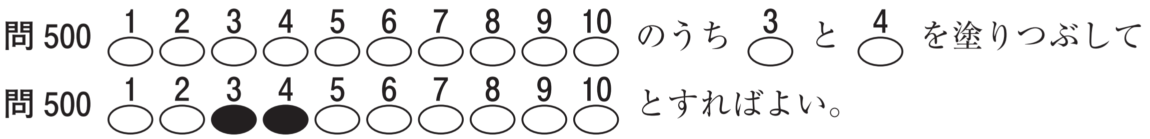

(2)　解答は、○の中全体をＨＢの鉛筆で濃く塗りつぶすこと。塗りつぶしが薄い場合は、解答したことにならないから注意すること。

悪い解答例

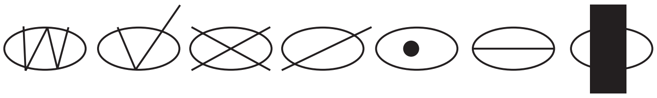

（採点されない）

(3)　解答を修正する場合は、必ず「消しゴム」で跡が残らないように完全に消すこと。鉛筆の跡が残ったり、「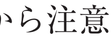」のような消し方などをした場合は、修正又は解答したことにならないから注意すること。

(4)　答案用紙は、折り曲げたり汚したりしないよう、特に注意すること。

3　設問中の科学用語そのものやその外国語表示（化合物名、人名、学名など）には誤りはないものとして解答すること。ただし、設問が科学用語そのもの又は外国語の意味の正誤の判断を求めている場合を除く。

4　問題の内容については質問しないこと。

一般問題（薬学理論問題）【薬理】

問 151　細胞内情報伝達系に関する記述のうち、正しいのはどれか。2つ選べ。

1　ヒスタミン H_1 受容体が刺激されると、Gs タンパク質を介してアデニル酸シクラーゼが活性化される。

2　インスリン受容体が刺激されると、インスリン受容体 β サブユニットの自己リン酸化が起こる。

3　インターフェロン α（IFN-α）受容体が刺激されると、JAK（ヤヌスキナーゼ）のチロシンキナーゼが活性化される。

4　オピオイド μ 受容体が刺激されると、Gq タンパク質を介してホスホリパーゼ C が活性化される。

5　心房性ナトリウム利尿ペプチド（ANP）受容体が刺激されると、可溶性グアニル酸シクラーゼが活性化される。

問 152　薬物依存及びその治療薬に関する記述のうち、正しいのはどれか。2つ選べ。

1　身体依存は、薬物の反復使用により、その効果が減弱し目的の効果を得るために増量しなければならなくなった状態である。

2　コカインの長期連用は、精神依存を起こすが、身体依存を起こしにくい。

3　慢性疼痛下のがん患者に適正に使用されたモルヒネは、精神依存を起こしにくい。

4　依存性薬物は、脳内報酬系におけるドパミン作動性神経を抑制する。

5　ジスルフィラムは、グルタミン酸 NMDA 受容体を遮断して飲酒欲求を抑制する。

問 153　自律神経系に作用する薬物に関する記述のうち、正しいのはどれか。**2つ**選べ。

1　メチルエフェドリンは、交感神経終末からのノルアドレナリンの遊離を抑制することで、血管平滑筋の収縮を抑制する。

2　ナフトピジルは、アドレナリン β_2 受容体を刺激することで、子宮平滑筋を弛緩させる。

3　イプラトロピウムは、アセチルコリン M_1 受容体を刺激することで、気管支平滑筋を弛緩させる。

4　メペンゾラートは、アセチルコリン M_3 受容体を遮断することで、下部消化管平滑筋の痙れん性収縮を抑制する。

5　アコチアミドは、アセチルコリンエステラーゼを阻害することで、低下した消化管運動を改善する。

問 154　中枢性及び末梢性筋弛緩薬に関する記述のうち、正しいのはどれか。**2つ**選べ。

1　エペリゾンは、γ-アミノ酪酸 $GABA_B$ 受容体を遮断して、脊髄における多シナプス反射を抑制する。

2　チザニジンは、アドレナリン α_2 受容体を刺激して、脊髄反射を抑制する。

3　ダントロレンは、神経筋接合部のアセチルコリン N_M 受容体を刺激して、持続的な脱分極を引き起こす。

4　ロクロニウムは、神経筋接合部のアセチルコリン N_M 受容体を競合的に遮断する。

5　A 型ボツリヌス毒素は、筋小胞体のリアノジン受容体に作用して、Ca^{2+} 遊離を抑制する。

問 155　全身麻酔薬及び催眠薬に関する記述のうち、正しいのはどれか。<u>２つ</u>選べ。

1　ゾピクロンは、メラトニン MT_1 受容体を刺激して、概日リズムを整える。

2　ミダゾラムは、γ-アミノ酪酸 $GABA_A$ 受容体の活性化を増強して、鎮静作用を示す。

3　デクスメデトミジンは、オレキシン受容体を遮断して、睡眠・覚醒サイクルを正常化する。

4　ケタミンは、グルタミン酸 NMDA 受容体を遮断して、鎮痛作用を示す。

5　チオペンタールは、オピオイド μ 受容体を刺激して、短時間の麻酔作用を示す。

一般問題（薬学理論問題）【薬理／病態・薬物治療】

問 156-157　22 歳女性。近医を受診し、以下の経過を訴えたところ、精神科を紹介された。

「仕事が多忙で残業が続いていたある日、通勤時に電車内で突然動悸が始まり、呼吸困難となり、今にも心臓が止まりそうになり、やっとの思いで次の駅で降りて救急車で病院へ運ばれたが、病院に着く頃には症状はだいぶ落ちついていた。念のため、診察を受けたが身体的には異常はなく、心電図や血液検査でも異常は認められなかった。１週間後、外出した時に、乗っていた電車の中で同じような動悸が始まり、一緒にいた友人に手を握ってもらって何とか我慢して家までたどり着いた。それ以来、発作が怖くて電車に乗れなくなった。電車通勤はやめて親に送り迎えをしてもらい、どうにか仕事には行くことができている。」

問 156（病態・薬物治療）

この疾患の病態と治療に関する記述のうち、正しいのはどれか。2つ選べ。

1　薬物治療は原則として一生涯続ける。

2　発作と判断するには、それが起こる状況の特定が必要である。

3　予期不安を合併する場合が多い。

4　恐怖の対象となっている場所や状況に対する曝露療法が有効である。

5　呼吸困難に対して、酸素の投与が必要である。

問 157（薬理）

この患者の治療に用いられる可能性のある薬物に関する記述のうち、正しいのはどれか。2つ選べ。

1　ロラゼパムは、γ-アミノ酪酸 $GABA_A$ 受容体複合体のベンゾジアゼピン結合部位に結合して、抗不安作用を示す。

2　セルトラリンは、アドレナリン β_1 受容体を遮断して、発作時の自律神経症状を改善する。

3　エチゾラムは、中枢のヒスタミン H_1 受容体を選択的に遮断して、静穏作用を示す。

4　アルプラゾラムは、セロトニン $5\text{-}HT_{1A}$ 受容体を刺激して、不安、焦燥、睡眠障害を改善する。

5　パロキセチンは、セロトニンの再取り込みを選択的に阻害して、抑うつ状態を改善する。

一般問題（薬学理論問題）【薬理】

問 158　アレルギー性疾患の治療に用いられる薬物に関する記述のうち、正しいのはどれか。２つ選べ。

1　オザグレルは、Th2 サイトカインの産生を抑制して、鎮痒効果を示す。

2　トラニラストは、ヒスタミン H_1 受容体を遮断して、アレルギー性鼻炎を改善する。

3　プランルカストは、ロイコトリエン受容体を遮断して、気管支ぜん息の発作を予防する。

4　セラトロダストは、トロンボキサン A_2（プロスタノイド TP）受容体を遮断して、気道過敏症の亢進を抑制する。

5　スプラタストは、トロンボキサン合成酵素を阻害して、じん麻疹を改善する。

一般問題（薬学理論問題）【薬理／病態・薬物治療】

問 159-160　60 歳男性。基礎疾患を指摘されたことはない。1 週間前から 1 日に数回めまいを感じるようになった。今朝、強いめまいとふらつきを覚え、救急外来を受診した。来院時の血圧は 118/84 mmHg、脈拍数 32 回/分であった。心電図では P 波と QRS 波が全く無関係に出現し、PP 間隔と RR 間隔がそれぞれ一定で、PR 間隔は不規則であった。また、P 波より QRS 波の出現頻度が少なかった。

問 159（病態・薬物治療）

この患者の初期治療に適切な薬物はどれか。**２つ**選べ。

1　アミオダロン
2　ニトログリセリン
3　ランジオロール
4　アトロピン
5　イソプレナリン

問 160（薬理）

抗不整脈薬に関する記述のうち、正しいのはどれか。**２つ**選べ。

1　ニフェカラントは、K^+ チャネルを遮断して、心筋の活動電位の持続時間を延長する。
2　ピルシカイニドは、アドレナリン β_1 受容体を遮断して、不応期を延長する。
3　ベラパミルは、Ca^{2+} チャネルを遮断して、房室伝導速度を低下させる。
4　プロカインアミドは、Na^+ チャネル及び K^+ チャネルを遮断して、心電図の QT 間隔を短縮する。
5　ジルチアゼムは、Na^+ チャネルを遮断して、心筋の活動電位の立ち上がり（第 0 相）を抑制する。

一般問題（薬学理論問題）【薬理】

問 161　泌尿器に作用する薬物に関する記述のうち、正しいのはどれか。2つ選べ。

1　ソリフェナシンは、アドレナリン α_{1A} 受容体を遮断して、前立腺平滑筋を弛緩させる。

2　ジスチグミンは、コリンエステラーゼを阻害して、アセチルコリンによる膀胱排尿筋の収縮を増強する。

3　シロドシンは、アセチルコリン M_3 受容体を遮断して、膀胱括約筋を収縮させる。

4　タダラフィルは、ホスホジエステラーゼVを活性化して、前立腺平滑筋を弛緩させる。

5　ミラベグロンは、アドレナリン β_3 受容体を刺激して、膀胱排尿筋を弛緩させる。

問 162　胃・十二指腸潰瘍治療薬に関する記述のうち、正しいのはどれか。2つ選べ。

1　ファモチジンは、胃の壁細胞に存在するヒスタミン H_2 受容体を遮断することで、胃運動促進作用を示す。

2　ボノプラザンは、K^+ と競合して H^+,K^+-ATPase を可逆的に阻害することで、胃酸分泌抑制作用を示す。

3　レバミピドは、ドパミン D_2 受容体を遮断することで、胃運動促進作用を示す。

4　ミソプロストールは、プロスタノイド EP 受容体を刺激することで、胃酸分泌抑制作用と胃粘液分泌促進作用を示す。

5　ピレンゼピンは、ペプシンに結合することで、その活性を抑制する。

問 163　２型糖尿病の治療に使用される薬物に関する記述のうち、正しいのはどれか。<u>２つ</u>選べ。

1　ミチグリニドは、スルホニル尿素受容体に結合して ATP 感受性 K^+ チャネルを遮断することで、膵 β 細胞の細胞膜を脱分極させる。

2　ピオグリタゾンは、ペルオキシソーム増殖剤応答性受容体 γ（PPARγ）を活性化することで、脂肪細胞の分化を促進する。

3　イプラグリフロジンは、ナトリウム-グルコース共輸送体２（SGLT2）を阻害することで、小腸でのグルコースの吸収を選択的に抑制する。

4　リナグリプチンは、グルカゴン様ペプチド-1（GLP-1）受容体を活性化することで、グルコース濃度依存的にインスリン分泌を促進する。

5　メトホルミンは、AMP 活性化プロテインキナーゼ（AMPK）を阻害することで、骨格筋でのグルコーストランスポーター4（GLUT4）の細胞膜への移行を促進する。

問 164　性ホルモン関連薬に関する記述のうち、正しいのはどれか。<u>２つ</u>選べ。

1　フルタミドは、前立腺細胞のアンドロゲン受容体を遮断して、前立腺がんの増大を抑制する。

2　メテノロンは、エストロゲン受容体を刺激して、再生不良性貧血を改善する。

3　エキセメスタンは、子宮内膜のエストロゲン受容体を遮断して、子宮内膜がんの増大を抑制する。

4　デガレリクスは、視床下部のエストロゲン受容体を遮断して、排卵を誘発する。

5　レトロゾールは、アロマターゼを阻害して、エストロゲン合成を阻害する。

一般問題（薬学理論問題）【薬理／病態・薬物治療】

問 165-166　60 歳男性。仕事が忙しく睡眠不足が続いていた。ある日、右側胸部にかゆみを伴った皮疹が現れ、強い痛みも生じたため受診し、帯状疱疹と診断された。

問 165（病態・薬物治療）

この患者に関する記述のうち、適切なのはどれか。<u>２つ</u>選べ。

1　疲労やストレスが発症の要因となった可能性が高い。
2　皮疹は血管に沿って全身に拡がっていく。
3　病原体は麻しんと同じである。
4　皮疹出現の約 2 週間前に感染したと考えられる。
5　副腎皮質ステロイド薬を用いる場合、抗ウイルス薬を併用する。

問 166（薬理）

抗ウイルス薬に関する記述のうち、正しいのはどれか。<u>２つ</u>選べ。

1　アメナメビルは、帯状疱疹ウイルスのヘリカーゼ・プライマーゼ複合体の DNA 依存性 ATPase 活性を阻害して、mRNA の合成を阻害する。
2　ガンシクロビルは、サイトメガロウイルスのチミジンキナーゼにより一リン酸化された後、宿主細胞キナーゼで三リン酸化体まで変換されて、ウイルスの RNA ポリメラーゼを阻害する。
3　オセルタミビルは、インフルエンザウイルスが宿主細胞から遊離する際に働くノイラミニダーゼを阻害して、インフルエンザウイルスの増殖を抑制する。
4　ホスカルネットは、サイトメガロウイルスの RNA ポリメラーゼのピロリン酸結合部位に結合して、RNA の合成を阻害する。
5　アシクロビルは、三リン酸化体に変換されて、帯状疱疹ウイルスに感染した宿主細胞内でデオキシグアノシン三リン酸（dGTP）と競合して、ウイルスの DNA ポリメラーゼを阻害する。

一般問題（薬学理論問題）【薬理】

問 167　急性白血病治療薬に関する記述のうち、正しいのはどれか。**2つ**選べ。

1　シクロホスファミドは、活性酸素を発生させて、DNA を切断する。

2　ビンクリスチンは、チュブリンの重合を促進して、微小管を安定化させる。

3　シタラビンは、細胞内で三リン酸化されて、DNA ポリメラーゼを阻害する。

4　ダウノルビシンは、RNA ポリメラーゼを特異的に阻害する。

5　イマチニブは、Bcr-Abl チロシンキナーゼを阻害する。

問 168　図のように薬物 **A** は酵素**ア**に作用する。薬物 **A** 及び酵素**ア**に関する記述のうち、正しいのはどれか。**2つ**選べ。

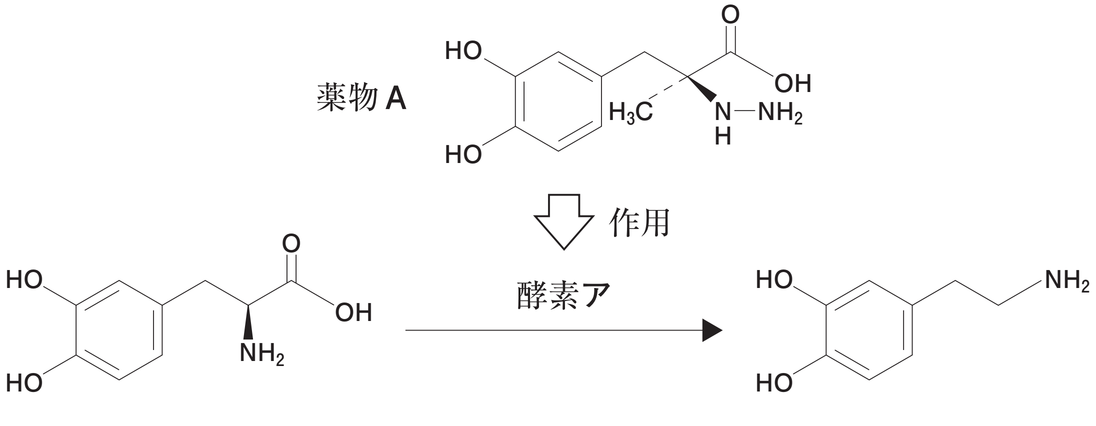

1　薬物 **A** は、酵素**ア**によって、ドパミンに異化される。

2　薬物 **A** は、酵素**ア**を阻害して、末梢でのレボドパからドパミンへの異化を抑制する。

3　薬物 **A** は、酵素**ア**を阻害して、脳内ドパミンの酸化を抑制する。

4　エンタカポンは、酵素**ア**を阻害して、末梢でのレボドパから 3-*O*-メチルドパへの異化を抑制する。

5　ドロキシドパは、酵素**ア**によってノルアドレナリンに異化される。

一般問題（薬学理論問題）【薬剤】

問 169　線形薬物動態を示す薬物A 10 mg を静脈内投与あるいは経口投与した後の血中濃度時間曲線下面積（AUC）は、それぞれ 500 ng・h/mL、150 ng・h/mL であった。経口投与後の薬物Aの消化管上皮細胞への移行率と肝抽出率を算出したところ、それぞれ 90% と 45% であった。また、胆汁中及び尿中に未変化体薬物は検出されなかった。薬物Aが消化管上皮細胞での代謝を免れる率として、最も近いのはどれか。1つ選べ。

1　10%
2　20%
3　30%
4　60%
5　75%

問 170　ある薬物のアルブミンへの結合に関する両逆数プロットを実線で表し、また、この薬物のアルブミンへの結合が別の薬物の共存により競合的に阻害された場合を点線で表すとき、正しい図はどれか。**1つ**選べ。

ただし、図中の r はアルブミン1分子あたりに結合している薬物の分子数を、［D_f］は非結合形薬物濃度を示す。

1

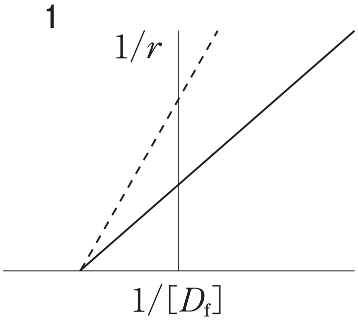

2

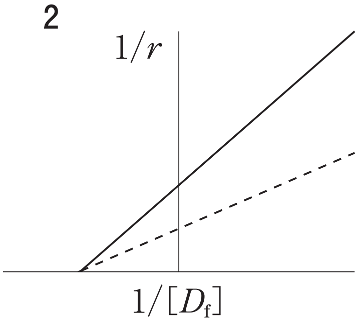

3

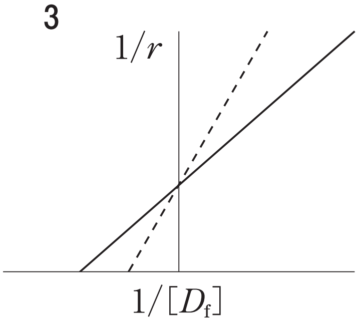

4

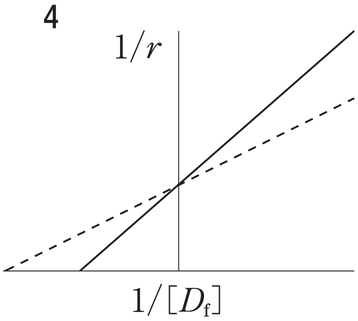

5

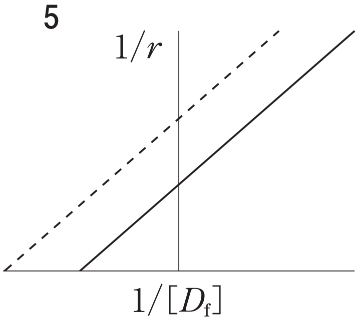

問 171　プラバスタチンの体内動態に関する記述のうち、正しいのはどれか。**2つ**選べ。

1　プラバスタチンは、シトクロム P450 による代謝を受けやすい。

2　プラバスタチンは、胆管側膜に存在する Multidrug Resistance-associated Protein 2（MRP2）により胆汁中に分泌される。

3　プラバスタチンは、キニジンとの併用により中枢移行量が増える。

4　プラバスタチンの血中濃度は、シクロスポリンとの併用により上昇する。

5　プラバスタチンは、有機カチオントランスポーター OCT1 を介して肝細胞内に取り込まれる。

問 172　細切した肝臓をホモジナイザーで破砕し遠心分離を繰り返すと細胞内小器官を大まかに分けることができる。主に薬物代謝に関わるシトクロム P450 が存在する画分として、最も適切なのはどれか。**1つ**選べ。

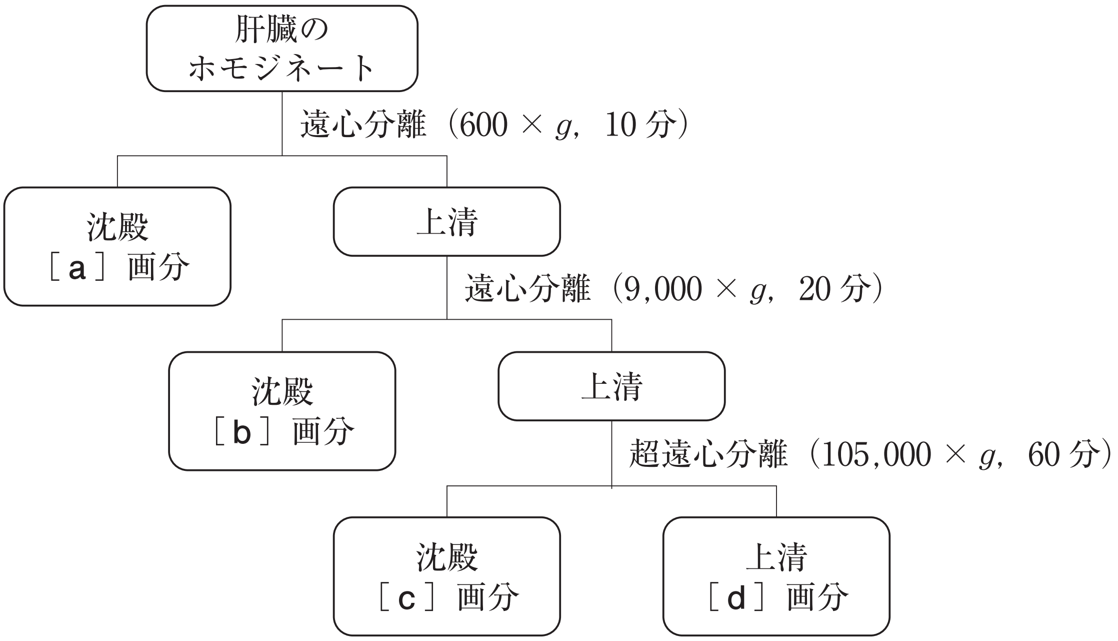

1　a
2　b
3　c
4　d
5　a から d のすべて

問 173　薬物の腸肝循環に関する記述のうち、正しいのはどれか。**２つ**選べ。

1　胆管閉塞で血中半減期が短縮する。
2　抗菌薬の内服による影響を受けることがある。
3　静脈内投与された薬物では起こらない。
4　腸内細菌の β-グルクロニダーゼが阻害されると血中半減期が延長する。
5　経口投与後の血中濃度において、ピークが二峰性を示すことがある。

問 174　薬物の血中濃度（C）の経時変化が下図のようになったため、体循環コンパートメントと末梢コンパートメントからなる線形 2-コンパートメントモデルで解析し、次の式の形で表した。

$$C = A \cdot e^{-\alpha \cdot t} + B \cdot e^{-\beta \cdot t}$$

ただし、A、B、α、β は定数、t は時間であり、投与量を D とする。このときの薬物動態パラメータに関する記述のうち、正しいのはどれか。**2つ**選べ。

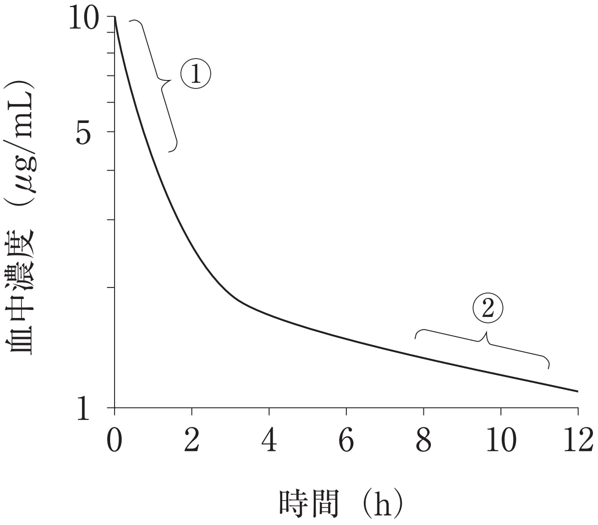

1　終末相（β 相）における消失速度定数（β）は、図の②の部分の傾きから求められる。

2　分布相（α 相）における消失速度定数（α）は、図の①の部分の傾きから求められる。

3　血中濃度時間曲線下面積は、$(A + B)/(\alpha + \beta)$ で表すことができる。

4　投与直後の薬物血中濃度は $A + B$ で表すことができる。

5　体循環コンパートメントの分布容積は D/A で表すことができる。

問 175　フェニトイン 100 mg 錠を 1 回 1 錠、1 日 3 回服用するところ、誤って 1 回 1 錠、1 日 2 回しか服用していなかった。その時の患者のフェニトインの血中濃度は 4 μg/mL であった。この患者が処方どおりに 1 日 3 回服用した場合のフェニトインの血中濃度（μg/mL）として、最も近い値はどれか。**1 つ**選べ。

ただし、フェニトインの血中濃度は定常状態における平均値であるものとし、フェニトインの体内からの消失は Michaelis–Menten 式で表され、Michaelis 定数を 4 μg/mL、バイオアベイラビリティを 100％とする。

1　6
2　12
3　16
4　20
5　24

問 176 図は、well-stirred model に基づいた肝臓からの薬物消失モデルを示したものである。このモデルに関する記述のうち、正しいのはどれか。**2つ**選べ。

ただし、CL_{int} は肝固有クリアランス、f_u は血中タンパク非結合形分率、C_{in} は肝臓に流入する部位における血中薬物濃度、C_{out} は肝臓から流出する部位における血中薬物濃度、Q_h は肝血流量とする。

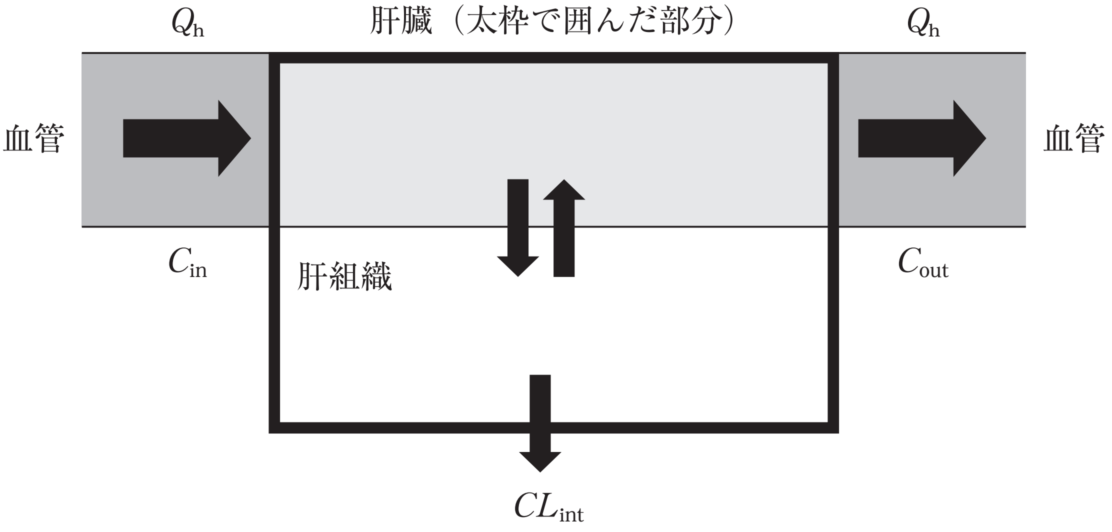

1 肝臓中の非結合形薬物濃度は不均一である。

2 肝組織中の非結合形薬物濃度は C_{in} に等しい。

3 肝臓からの見かけの薬物消失速度は、$CL_{int} \times f_u \times C_{out}$ と表される。

4 肝臓における薬物量の変化速度は、$Q_h \times C_{in} - Q_h \times C_{out} - CL_{int} \times f_u \times C_{out}$ と表される。

5 定常状態における CL_{int} は Q_h にほぼ等しい。

問 177　粉体の性質に関する記述のうち、正しいのはどれか。２つ選べ。

1　顕微鏡法により得られた粒子の投影像を一定方向の２本の平行線で挟んだとき、平行線間の長さに相当する粒子径をマーチン径という。

2　同一粉体において、質量基準による粒度分布の平均粒子径より、個数基準による粒度分布の平均粒子径の方が小さい。

3　水溶性の結晶性粉体の臨界相対湿度は、水不溶性の結晶性粉体と混合することで低下する。

4　真密度 1.4 g/cm^3、空隙率 0.5 の粉末 70 g の空隙体積が 2/5 になるまで圧縮した際のみかけの密度は 1.0 g/cm^3 である。

5　試料粉体の比表面積と平均粒子径が比例することから、比表面積を測定することで試料粉体の平均粒子径を求めることができる。

問 178 固体薬物の溶解速度を回転円盤法を用いて温度一定の条件で測定したところ、図のような結果となった。試験液中の薬物濃度（C）が薬物の溶解度（Cs）の半分に達するまでの時間（min）に最も近いのはどれか。**1つ**選べ。

ただし、実験開始時の試験液中の薬物濃度は 0 、円盤の有効表面積（1 cm^2）は試験中に変化せず、溶解はシンク条件において拡散律速で進行するものとする。なお、ln2 ＝ 0.69 とする。

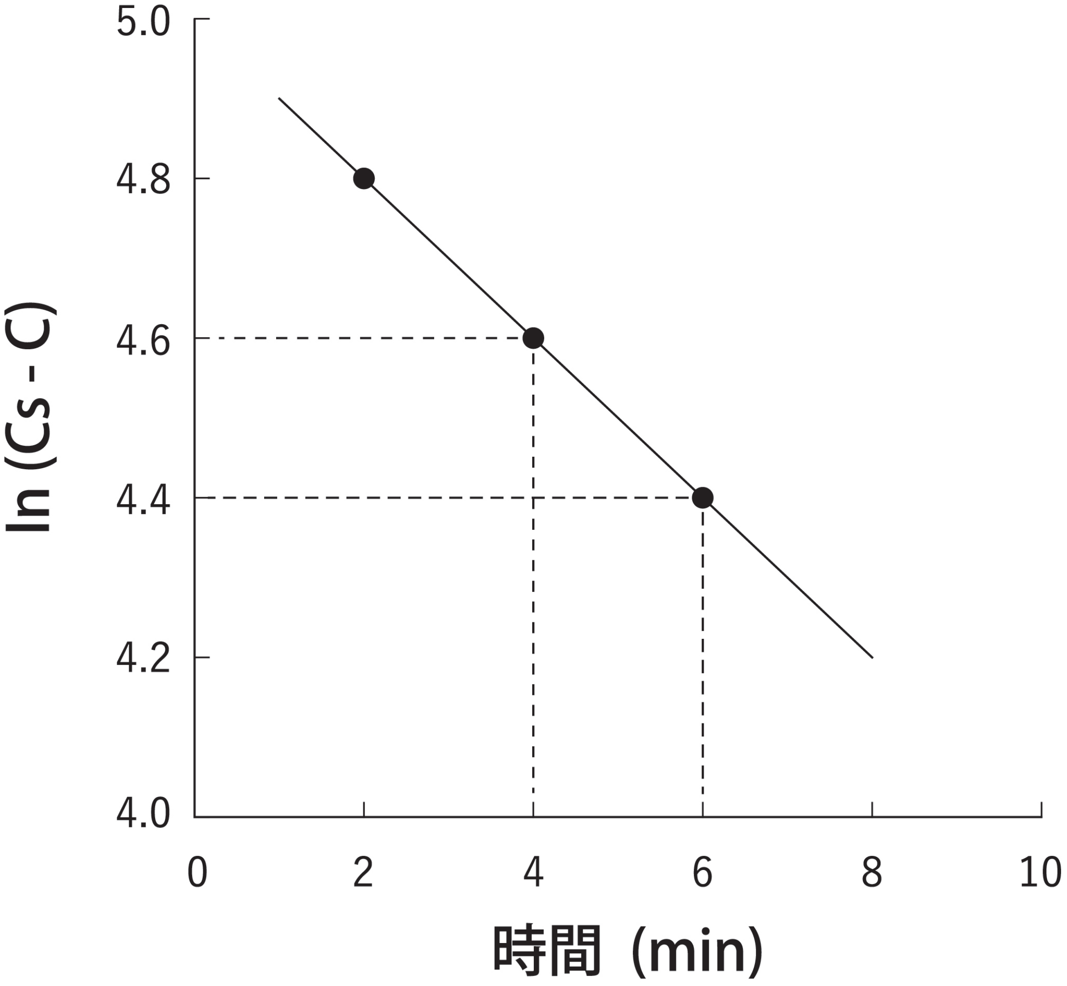

1 3.5
2 7.0
3 10.5
4 14.0
5 17.5

問 179 図の装置を用いて、懸濁剤に分散している球状の粉末粒子の粒子径を測定した。本測定に関する記述のうち、正しいのはどれか。**2つ**選べ。

ただし、分散媒、分散粒子の密度はそれぞれ 1.0 g/cm^3、2.0 g/cm^3 とする。

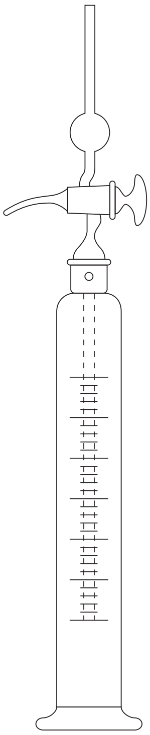

1 アンドレアゼンピペットを用いた沈降法による粒子径測定である。

2 ラングミュア式を利用して粒子径を算出する。

3 コロイド粒子の粒子径を測定することができる。

4 懸濁剤に分散している粒子径を 1/3 にすると、分散粒子の沈降速度は 1/3 になる。

5 増粘剤添加により分散媒の粘度を 1.6 倍、分散媒の密度を 1.2 g/cm^3 にすると、分散粒子の沈降時間は 2 倍になる。

問 180　図は、pH 7.4、37 ℃の緩衝液中におけるある弱酸性薬物の加水分解に対するシクロデキストリン添加の影響を示したものである。本実験条件において、この薬物とシクロデキストリンはモル比 1：1 で複合体を形成する。

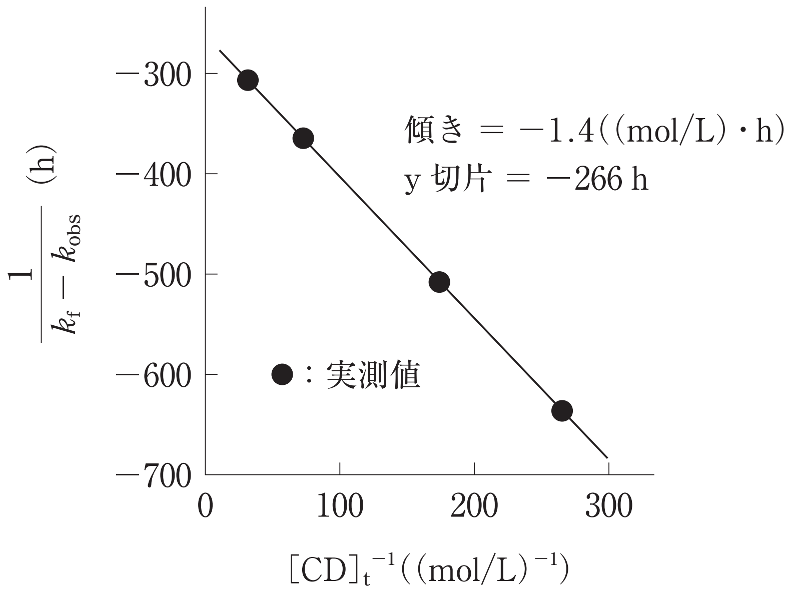

ここで、k_f は薬物自体の分解速度定数（$2.26 \times 10^{-3}\ h^{-1}$）、$k_{obs}$($h^{-1}$) は見かけの薬物分解速度定数（$h^{-1}$）、$K_{1:1}$ は複合体の安定度定数（(mol/L)$^{-1}$）、k_c は複合体中の薬物分解速度定数（h^{-1}）、$[CD]_t$ はシクロデキストリンの総濃度（mol/L）としたとき、次のような関係式が成立する。以下の記述のうち、正しいのはどれか。<u>2つ</u>選べ。

$$\frac{1}{k_f - k_{obs}} = \frac{1}{K_{1:1}(k_f - k_c)} \cdot \frac{1}{[CD]_t} + \frac{1}{k_f - k_c}$$

1　この薬物との複合体の安定度定数は 190(mol/L)$^{-1}$ である。

2　複合体中の薬物の加水分解速度定数は $6.02 \times 10^{-1}\ h^{-1}$ である。

3　シクロデキストリンの添加濃度の上昇にしたがい、この薬物の見かけの加水分解速度定数は増大する。

4　シクロデキストリンは、この薬物の加水分解に対して安定化効果を示す。

5　複合体形成によるこの薬物の安定化効果は pH によって変化しない。

問 181　皮膚に使用する製剤に関する記述のうち、正しいのはどれか。<u>２つ</u>選べ。

1　油脂性基剤である単軟膏や白色軟膏は、乾燥型及び湿潤型いずれの皮膚疾患にも使用できる。

2　水溶性基剤であるマクロゴールは、分子量が大きくなると水に不溶になる。

3　水溶性ゲル基剤は、損傷皮膚を含めた様々な状態の皮膚に安全に使用できる。

4　吸水クリームと親水クリームは、いずれもサラシミツロウを含んでいる。

5　マトリックス型のテープ剤は、日本薬局方の粘着力試験法により評価される。

問 182　フルチカゾンプロピオン酸エステルを有効成分とする懸濁性点鼻液に含まれる添加物に関する記述のうち、正しいのはどれか。<u>２つ</u>選べ。

〔添加物〕カルボキシビニルポリマー、L-アルギニン、ベンザルコニウム塩化物、エデト酸ナトリウム水和物、ポリソルベート 80、濃グリセリン、塩化ナトリウム、水酸化ナトリウム、精製水

1　ポリソルベート 80 は、懸濁化剤として添加されている。

2　ベンザルコニウム塩化物は、有効成分を可溶化するために添加されている。

3　塩化ナトリウムは、微生物の増殖を抑制するために添加されている。

4　カルボキシビニルポリマーは、有効成分の鼻腔内滞留性を高めるために添加されている。

5　エデト酸ナトリウム水和物は、無痛化剤として添加されている。

問 183　薬物とターゲティング技術に関する記述として、正しいのはどれか。__２つ__選べ。

1　レボドパは、主にP-糖タンパク質により選択的に脳内に取り込まれる。

2　サラゾスルファピリジンは、腸内細菌により5-アミノサリチル酸に変換される。

3　フルシトシンは、腫瘍細胞内の酵素により5-フルオロウラシルに変換される。

4　アルプロスタジルは、乳酸・グリコール酸共重合体マイクロスフェアを担体として病変部位にターゲティングされる。

5　ガラクトシル人血清アルブミンジエチレントリアミン五酢酸テクネチウムは、肝細胞膜上のアシアロ糖タンパク質受容体に強く結合する。

一般問題（薬学理論問題）【病態・薬物治療】

問 184 スティーブンス・ジョンソン症候群に関する記述のうち、正しいのはどれか。**2つ**選べ。

1 中毒性表皮壊死症とも呼ばれる。
2 内服薬よりも、皮膚外用薬によって発症するケースが多い。
3 視力低下をきたすことがある。
4 発熱や倦怠感などの全身症状を伴うことはまれである。
5 治療の基本は、副腎皮質ステロイド薬の全身投与である。

問 185 ギラン・バレー症候群に関する記述のうち、正しいのはどれか。**2つ**選べ。

1 上気道感染症や消化器感染症の後に発症することが多い。
2 主に中枢神経の軸索や髄鞘が障害される。
3 原因病原体として最も多いのは真菌である。
4 下肢から上行する左右対称性の弛緩性運動麻痺がみられる。
5 副腎皮質ステロイド薬の単独療法により寛解が得られる。

問 186 貧血の病態に関する記述のうち、正しいのはどれか。**2つ**選べ。

1 鉄欠乏性貧血では、血清フェリチン値が上昇する。
2 巨赤芽球性貧血は、ビタミン B_{12} や葉酸の欠乏により起こる。
3 再生不良性貧血では、汎血球減少がみられる。
4 自己免疫性溶血性貧血は、大球性貧血に分類される。
5 腎性貧血では、エリスロポエチンの産生が亢進している。

問 187　ネフローゼ症候群で必ずみられるのはどれか。２つ選べ。

1　体重減少
2　血尿
3　タンパク尿
4　低アルブミン血症
5　低コレステロール血症

問 188　42 歳女性。最近、疲れやすいと感じることが多くなり、また徐々に食欲が低下し、何をするにも億劫でやる気が起こらなくなった。月経周期が乱れたため受診し、検査の結果、橋本病と診断された。この患者の状態として、考えられるのはどれか。２つ選べ。

1　体重が著しく減少している。
2　頻脈が認められる。
3　高コレステロール血症が認められる。
4　血清 TSH（甲状腺刺激ホルモン）値が高い。
5　副甲状腺ホルモンの分泌が亢進している。

問 189　緑内障に関する記述のうち、正しいのはどれか。２つ選べ。

1　緑内障は、眼圧を正常範囲内に維持すれば症状は現れない。
2　緑内障により低下した視力は、眼圧を低下させると速やかに回復する。
3　開放隅角緑内障では、虹彩と水晶体の間の房水の流れが妨げられている。
4　開放隅角緑内障では、視野欠損が徐々に進行する。
5　急性閉塞隅角緑内障では、急性の眼痛とともに頭痛、悪心・嘔吐を伴った視力低下が起こる。

問 190　メニエール病の病態と治療に関する記述のうち、正しいのはどれか。**２つ**選べ。

1　ウイルス感染症である。
2　反復性回転性めまいと耳鳴、難聴や耳閉感を併発することが多い。
3　イソソルビドは、外リンパ圧低下作用によりめまいを抑制する。
4　ペルフェナジンは、悪心・嘔吐を抑制する。
5　ベタヒスチンメシル酸塩は、外耳の血液循環を改善することによりめまいを抑制する。

問 191　B型インフルエンザに続発するライ（Reye）症候群に関する記述として、正しいのはどれか。**１つ**選べ。

1　異常型プリオン蛋白が脳内に蓄積して発症する。
2　肝障害を伴う急性脳症である。
3　血糖値の上昇がみられる。
4　重度の黄疸がみられる。
5　小児の場合、アスピリンが治療に用いられる。

問 192　漢方薬に関する記述のうち、正しいのはどれか。**２つ**選べ。

1　大建中湯は、不眠症に使用される。
2　五苓散は、浮腫に使用される。
3　小青竜湯は、アレルギー性鼻炎に使用される。
4　補中益気湯は、月経困難に使用される。
5　抑肝散は、気管支ぜん息に使用される。

問 193　幹細胞に関する記述のうち、正しいのはどれか。**２つ**選べ。

1　胚性幹細胞（ES 細胞）は、自己複製能を持つ。

2　胚性幹細胞は、多能性獲得に必要な遺伝子を導入して作製する。

3　人工多能性幹細胞（iPS 細胞）の作製には、受精卵が用いられる。

4　造血幹細胞は、あらゆる細胞に分化する能力を持つ。

5　造血幹細胞は、臍帯血にも存在する。

問 194　EBM の実践に関する記述のうち、正しいのはどれか。**２つ**選べ。

1　最初のプロセスは「問題解決のための情報収集」である。

2　情報を効率的に収集するために、一次資料の調査から開始する。

3　PICO 又は PECO とよばれる４つの要素（Patient、Intervention/Exposure、Comparison、Outcome）を用いて問題の定式化を行う。

4　論文等に示された研究成果の正確度や再現性を確認することを、内的妥当性の評価という。

5　割りつけられた治療を完遂できず脱落した者を除いた解析は ITT（intention-to-treat）解析という。

問 195 試験期間が12ヶ月の臨床試験に参加した５名の被験者（**A**～**E**）の経過が以下のようになった。

Aが２ヶ月後に死亡
Bが８ヶ月後に死亡
Cが12ヶ月後の試験終了時まで生存
Dが４ヶ月後に追跡不能となり打ち切り
Eが６ヶ月後に追跡不能となり打ち切り

カプランマイヤー法を用いて表した生存曲線として、正しいのはどれか。**1**つ選べ。

1

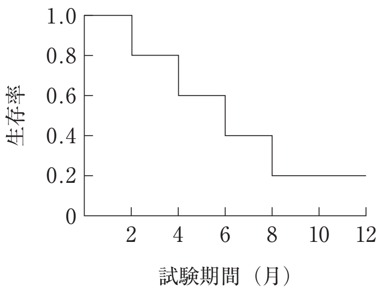

2

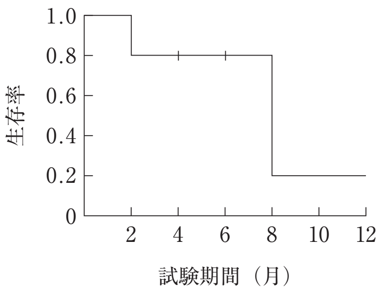

3

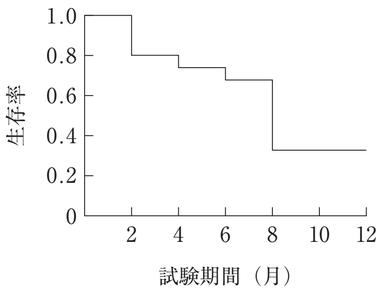

4

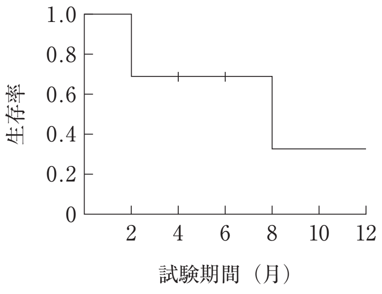

5

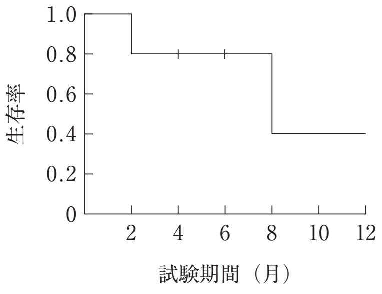

6

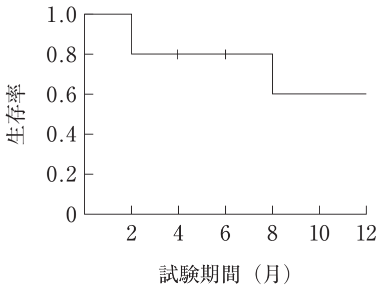

107

2日目①　　一般問題（薬学実践問題）

【物理・化学・生物、衛生／実務】

◎指示があるまで開いてはいけません。

注　意　事　項

1　試験問題の数は、**問１９６**から**問２４５**までの**５０問**。
９時３０分から**１１時３５分**までの**１２５分以内**で解答すること。

2　解答方法は次のとおりである。

(1)　一般問題（薬学実践問題）の各問題の正答数は、**問題文中に指示されている**。
問題の選択肢の中から答えを選び、次の例にならって答案用紙に記入すること。
なお、問題文中に指示された正答数と**異なる数を解答すると、誤りになる**から注意すること。

（例）**問 500**　次の物質中、常温かつ常圧下で液体のものはどれか。２つ選べ。

1　塩化ナトリウム　　2　プロパン　　3　ベンゼン
4　エタノール　　5　炭酸カルシウム

正しい答えは「3」と「4」であるから、答案用紙の

問 500　1　2　3　4　5　6　7　8　9　10　のうち 3 と 4 を塗りつぶして

問 500　1　2　3　4　5　6　7　8　9　10　とすればよい。

(2)　解答は、◯の中全体をＨＢの鉛筆で濃く塗りつぶすこと。塗りつぶしが薄い場合は、解答したことにならないから注意すること。

悪い解答例

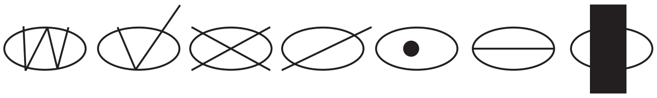

（採点されない）

(3)　解答を修正する場合は、必ず「消しゴム」で跡が残らないように完全に消すこと。鉛筆の跡が残ったり、「　」のような消し方などをした場合は、修正又は解答したことにならないから注意すること。

(4)　答案用紙は、折り曲げたり汚したりしないよう、特に注意すること。

3　設問中の科学用語そのものやその外国語表示（化合物名、人名、学名など）には誤りはないものとして解答すること。ただし、設問が科学用語そのもの又は外国語の意味の正誤の判断を求めている場合を除く。

4　問題の内容については質問しないこと。

一般問題（薬学実践問題）【物理・化学・生物、衛生／実務】

問 196-197　17 歳男性。身長 175 cm、体重 72 kg。悪性軟部肉腫に対し、以下の処方で初期治療を行うことになった。

（処方）

ドキソルビシン塩酸塩注射用　120 mg
生理食塩液 50 mL　1 本
30 分かけて点滴静注

問 196（実務）

この処方を調製する際には、難溶性の凝集体が生成することがある。確実に溶解させるための操作として、適切なのはどれか。**２つ**選べ。なお、処方にない溶解液を用いる場合は、医師に確認した上で行うものとする。

1　微量の生理食塩液をゆっくり加えて攪拌後、生理食塩液 50 mL に混合する。
2　溶解に必要な量の生理食塩液を素早く加えて攪拌後、生理食塩液 50 mL に混合する。
3　溶解に必要な量の注射用水を素早く加えて攪拌後、生理食塩液 50 mL に混合する。
4　溶解に必要な量の７％炭酸水素ナトリウム液を素早く加えて攪拌後、生理食塩液 50 mL に混合する。
5　溶解に必要な量の 10％塩化ナトリウム液を素早く加えて攪拌後、生理食塩液 50 mL に混合する。

問 197（物理・化学・生物）

前問の難溶性凝集体が生成する相互作用として、適切なのはどれか。**1つ**選べ。なお、ドキソルビシン塩酸塩の構造式は以下のとおりである。

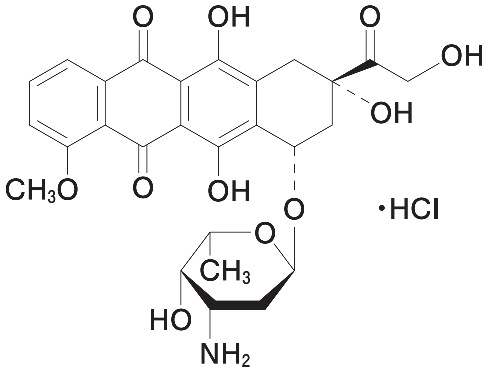

1　$\pi-\pi$ スタッキング

2　配位結合

3　疎水性相互作用

4　水素結合

5　電荷移動相互作用

問 198-199　20 歳女性。身長 158 cm、体重 38 kg。貧血症状に対して入院加療することになった。入院時の所見は次のとおりであり、注射剤による治療が開始された。

（身体所見）

体温 36.3 ℃、血圧 108/62 mmHg、脈拍数 95 拍/分（整）、顔面蒼白

（検査所見）

白血球数 3,500/μL、赤血球数 240 × 10^4/μL、Hb 6.0 g/dL、Ht 21%、
血小板数 22 × 10^4/μL、血清鉄（SI）3.4 μg/dL、総鉄結合能（TIBC）
360 μg/dL、フェリチン 8.9 ng/mL、AST 18 IU/L、ALT 16 IU/L、
総ビリルビン 0.4 mg/dL、直接ビリルビン 0.2 mg/dL

（処方）

含糖酸化鉄注射液 40 mg/2 mL（注）　1 回 2 管
1 日 1 回　午前 9 時　静脈注射

（注）コロイド性静脈注射用鉄剤、$[Fe(OH)_3]_m$ $[C_{12}H_{22}O_{11}]_n$、pH 9.0～10.0、
1 管 2 mL は鉄として 40 mg に相当

問 198（実務）

薬剤師は、鉄の過剰投与を防止するため、調剤に先立ち、総投与鉄量を計算し、投与期間を確認することにした。投与期間として最も近いのはどれか。**1つ**選べ。
ただし、総投与鉄量（貯蔵鉄を加えた鉄量）の計算式は次のとおりとする。

総投与鉄量（mg）$= \{2.72 \times (16 - X) + 17\} \times W$
ここで X はヘモグロビン値（g/dL）、W は体重（kg）である。

1　2日間
2　10日間
3　20日間
4　30日間
5　40日間

問 199（物理・化学・生物）

処方されたコロイド性静脈注射用鉄剤に関する記述のうち、正しいのはどれか。**2つ**選べ。

1　疎水コロイドを形成する水酸化鉄（Ⅲ）を糖で安定化させた鉄剤である。
2　静脈注射後、コロイドはすぐに不安定化し、鉄イオンが遊離する。
3　コロイドを不安定化させないように、希釈する場合は pH の変化に注意する必要がある。
4　希釈する場合は、イオン強度を上げるために塩化ナトリウム液を加えて5％以上の塩濃度を維持する。

問 200-201　68歳女性。痰を伴う咳、発熱、悪寒、息苦しさ、倦怠感を訴え、かかりつけ医を受診した。

（身体所見）

体温 38.5 ℃、経皮的動脈血酸素飽和度（SpO_2）94％、心音 異常なし、
呼吸音 左肺前胸部に水泡音、胸部 X 線 肺浸潤影あり

（検査所見）

白血球数 16,000/μL、CRP 4.8 mg/dL

副作用歴 ペニシリン系抗生物質により発疹

以上の情報から、市中肺炎と診断された。

問 200（実務）

この患者に推奨される抗菌剤はどれか。**2つ**選べ。

1　クラブラン酸カリウム・アモキシシリン水和物配合錠
2　レボフロキサシン水和物錠
3　ホスホマイシンカルシウム水和物錠
4　カナマイシン一硫酸塩カプセル
5　アジスロマイシン水和物錠

問 201（物理・化学・生物）

前問の抗菌剤投与により、症状が改善し、SpO_2 が 94%から 97%になった。この SpO_2 の測定には、パルスオキシメータが用いられている。パルスオキシメータによる SpO_2 の測定及びその値に関する記述のうち、誤っているのはどれか。**1つ**選べ。

1　パルスオキシメータによる SpO_2 の測定には赤色光と赤外光が用いられる。
2　酸素と結合したヘモグロビン（HbO_2）と、酸素と結合していないヘモグロビン（Hb）の濃度は、どちらも測定時に用いられる 2 つの波長における吸光度より求められる。
3　SpO_2（%）は、$\dfrac{[HbO_2]}{[Hb]+[HbO_2]}\times 100$ で定義される。
4　原理的に親指、人差し指、小指で測定した SpO_2 値に違いはない。
5　この患者の動脈血酸素分圧（PaO_2）は、抗菌剤投与によって 1.03 倍（= 97/94）になった。

問 202-203　60 歳女性。背中の粉瘤 (注) が感染を起こしたため皮膚科を受診し、以下の処方箋を持って薬局を訪れた。

（注）粉瘤（アテローム）：皮膚の下に袋状の嚢腫ができ、本来皮膚から剥げ落ちるはずの垢（角質）と皮膚の脂（皮脂）が、剥げ落ちずに袋の中にたまってしまってできた腫瘍の総称。

（処方）

セフジニルカプセル 100 mg	1 回 1 カプセル（1 日 3 カプセル）
	1 日 3 回　朝昼夕食後　5 日分

また、患者が持参したお薬手帳から、以下の薬剤を服用中であることがわかった。

エナラプリルマレイン酸塩錠 5 mg	1 回 1 錠（1 日 1 錠）
	1 日 1 回　朝食後　28 日分
L-アスパラギン酸 Ca 錠 200 mg	1 回 2 錠（1 日 6 錠）
レバミピド錠 100 mg	1 回 1 錠（1 日 3 錠）
	1 日 3 回　朝昼夕食後　28 日分
クエン酸第一鉄ナトリウム錠 50 mg	1 回 2 錠（1 日 2 錠）
プラバスタチン Na 錠 10 mg	1 回 1 錠（1 日 1 錠）
	1 日 1 回　夕食後　28 日分

問 202（実務）

今回処方された医薬品と併用するにあたり、注意が必要な服用中の薬剤はどれか。**1つ**選べ。

1　エナラプリルマレイン酸塩錠
2　L-アスパラギン酸 Ca 錠
3　レバミピド錠
4　クエン酸第一鉄ナトリウム錠
5　プラバスタチン Na 錠

問 203（物理・化学・生物）

セフジニルには不斉炭素があり、旋光性を示すので、旋光度測定で確認することができる。日本薬局方セフジニル（$C_{14}H_{13}N_5O_5S_2$：395.41）の旋光度の項には、以下のように記されている。

$[\alpha]_D^{20}$：$-58\sim-66^\circ$ (0.25 g、pH 7.0 の 0.1 mol/L リン酸塩緩衝液、25 mL、100 mm).

以下の記述のうち、正しいのはどれか。**2つ**選べ。

1　日本薬局方では、旋光度の測定には、通例、光源として重水素放電管が用いられる。
2　セフジニルは右旋性である。
3　試料中に前問の併用注意薬物が共存する場合でも、セフジニル自体の比旋光度は変わらない。
4　この条件下で測定した場合、日本薬局方セフジニルの旋光度の範囲は、$-0.58\sim-0.66^\circ$ である。
5　層長 200 mm の測定管を用いると、測定されるセフジニルの旋光度の値は 1/2 になる。

問 204-207　50 歳男性。高血圧の治療のため、近隣の内科クリニックに通院中である。喫煙歴 30 年（1 日 40 本）。かかりつけ薬剤師に患者から電話相談があり、「昨日、晴天の中ゴルフに出かけたところ、衣服から露出した部分が赤く日焼けのようになった」と相談があった。薬剤師が薬剤服用歴を確認したところ、光線過敏症の可能性が疑われたので、皮膚科受診を勧めた。

4 月 20 日　　処方内容（内科）

テモカプリル塩酸塩錠 4 mg	1 回 1 錠（1 日 1 錠）
ヒドロクロロチアジド錠 12.5 mg	1 回 1 錠（1 日 1 錠）
	1 日 1 回　朝食後　28 日分
ゾルピデム酒石酸塩錠 5 mg	1 回 1 錠（1 日 1 錠）
	1 日 1 回　就寝直前　28 日分
モサプリドクエン酸塩錠 5 mg	1 回 1 錠（1 日 3 錠）
	1 日 3 回　朝昼夕食後　28 日分
ケトプロフェンテープ 20 mg	1 回 2 枚（1 日 2 枚）
	1 日 1 回　朝　腕・腰に貼付　7 日分

問 204（実務）

皮膚症状の原因として、考えられる薬剤はどれか。<u>２つ</u>選べ。

1　テモカプリル塩酸塩錠
2　ヒドロクロロチアジド錠
3　ゾルピデム酒石酸塩錠
4　モサプリドクエン酸塩錠
5　ケトプロフェンテープ

問 205（実務）

前述の患者が皮膚科を受診し、光線過敏症の診断を受け、以下の処方箋を持って薬局を訪れた。

（処方）

トプシムスプレー 0.0143%(注)　28 g　1 缶

1 回適量　1 日 2 回　朝夕　腕に噴霧

（注）有効成分 1 g 中にフルオシノニド 0.143 mg を含有する噴霧剤

皮膚科から処方された噴霧剤に関する説明として、適切なのはどれか。２つ選べ。

1 炎症に伴う発赤、腫れ、かゆみなどの症状を改善します。

2 患部に水疱ができている場合は使用しないでください。

3 患部に傷がある場合でも使用できます。

4 目の周りの症状にも使用できます。

5 たばこなどの火気を避けて使用してください。

問 206（物理・化学・生物）

光線過敏症は、体表面に近い部分に分布した薬物が電磁波を吸収することにより誘発される。光線過敏症を誘発する電磁波に関する記述のうち、正しいのはどれか。２つ選べ。

1　キセノンランプが放射する光に含まれる。

2　原子核のスピン遷移に伴い吸収・放射される。

3　水分子の回転運動を直接引き起こす。

4　SPECT や PET に利用される。

5　$n-\pi^*$遷移や$\pi-\pi^*$遷移を引き起こす。

問 207（物理・化学・生物）

薬剤師は患者に対し、今後の対応として日焼け止め剤の利用を勧めることにした。日焼け止め剤に含まれている化合物のうち、光線過敏症の発症に予防的に機能することが期待されるものとして、適切でないのはどれか。**1つ**選べ。

1

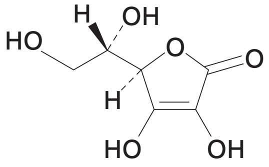

2

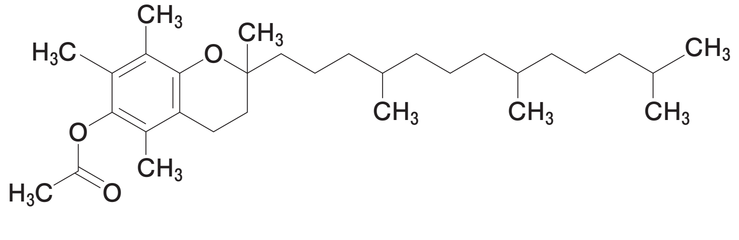

3

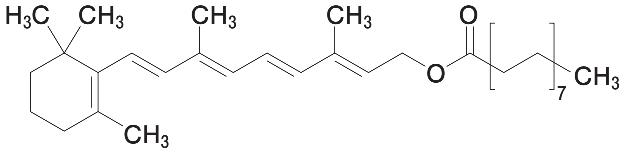

4

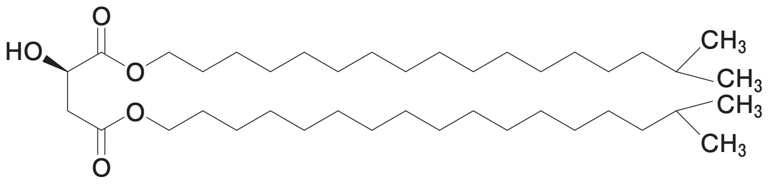

5

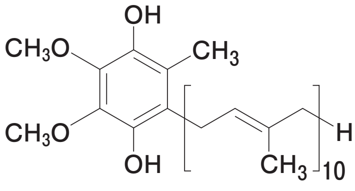

問 208-209　45歳男性。喫煙歴20年（1日20本）。20歳代前半から血清コレステロールの高値を指摘されていたが、未治療のまま放置していた。男性は、会社の健康診断でLDL-C値が220 mg/dLであると指摘され、年齢のことも考慮し近医を受診した。家族性高コレステロール血症と診断され、医師や薬剤師による生活習慣指導及び処方1による薬物治療が6ヶ月継続された。しかし、LDL-C値が管理目標まで下がらなかったため、本日の診察で薬剤の追加が検討された。生化学検査の結果、AST、ALT、総ビリルビンが高値を示し肝障害が疑われたため、処方2が追加された。なお、アドヒアランスは良好である。

（処方1）

ロスバスタチン錠5 mg	1回4錠（1日4錠）
	1日1回　就寝前　28日分

（処方2）

コレスチミド顆粒83%	1回1.81 g（1日3.62 g）
	1日2回　朝夕食前　28日分

（本日の検査値）

血圧122/74 mmHg、LDL-C 130 mg/dL、HDL-C 40 mg/dL、
TG（トリグリセリド）100 mg/dL、AST 120 IU/L、ALT 125 IU/L、
総ビリルビン2.0 mg/dL、HbA1c 5.5%（NGSP値）

問 208（実務）

生活習慣指導及び服薬指導の内容として、<u>適切でない</u>のはどれか。1つ選べ。

1 薬物治療だけでなく、禁煙することも重要です。

2 無酸素運動を中心に、毎日運動することが推奨されています。

3 家族性高コレステロール血症の LDL-C 管理目標は、高 LDL コレステロール血症の一次予防の目標より低く設定されています。

4 お腹の痛みや張りを感じたときは、すぐに処方医又は薬剤師に連絡してください。

5 今回追加された薬剤は、脂溶性ビタミンの吸収を低下させる可能性があります。

問 209（物理・化学・生物）

コレスチミドは腸管において、胆汁酸であるタウロコール酸の再吸収を阻害し、肝におけるコレステロールから胆汁酸への異化を促進する。タウロコール酸の再吸収が阻害される機序に関する記述として、最も適切なのはどれか。1つ選べ。

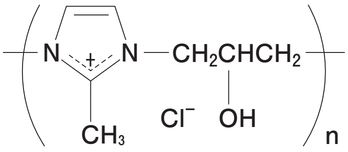

コレスチミド

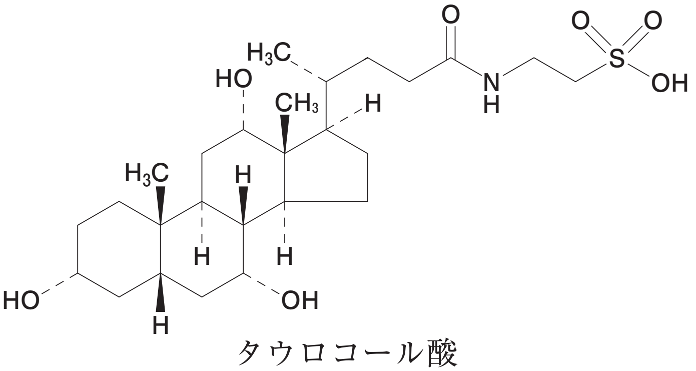

タウロコール酸

1 コレスチミドを触媒としてタウロコール酸が分解される。

2 コレスチミドのヒドロキシ基とタウロコール酸のヒドロキシ基との間に水素結合が形成される。

3 コレスチミドのカチオンとタウロコール酸のイオン化したスルホ基との間にイオン結合が形成される。

4 コレスチミドのヒドロキシ基とタウロコール酸のスルホ基との間に水素結合が形成される。

5 コレスチミドのヒドロキシ基とタウロコール酸のスルホ基がエステル結合を形成する。

問 210-211　35 歳女性。喫煙歴 15 年（1 日 20 本）。以前から、ニコチンガムやニコチンパッチによる禁煙を試みたが失敗を繰り返していた。今回、禁煙外来を受診し、ニコチン受容体の部分刺激薬であるバレニクリン酒石酸塩錠による禁煙を試みることになった。女性は、医療機関でニコチン置換療法とは異なる治療法であると説明を受け禁煙に意欲的だが、また失敗するのではないかと不安にもなっている。

問 210（実務）

薬剤師は患者の不安を和らげるため、今回の禁煙療法の特徴について説明した。説明内容として、適切なのはどれか。2つ選べ。

1　ニコチン置換療法と異なり、治療開始時は薬を服用しながら喫煙が可能です。
2　ニコチンを補充しないので、治療中に抑うつ気分や不安、イライラが強く出ることがありますが、一時的なものですので、そのまま服用を続けてください。
3　お薬にはニコチンが含まれていませんが、禁煙による離脱症状やタバコに対する切望感が軽減します。
4　途中で禁煙がつらくなったときは、ニコチンパッチ剤との併用療法に切り替えることができます。
5　ニコチンガムと同じように、主に口腔粘膜から有効成分が吸収されるので、炭酸飲料やコーヒーでの服用は避けてください。

問 211（物理・化学・生物）

禁煙療法に用いられた薬物の構造から、ニコチン性アセチルコリン受容体との相互作用に関わる化学的性質として、正しいのはどれか。2つ選べ。

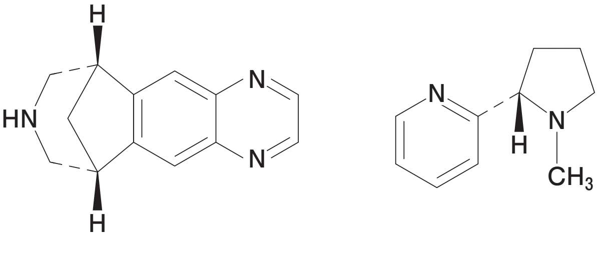

バレニクリン　　　　ニコチン

1　バレニクリンの共役酸の p*K*a は 4 付近である。
2　共に生体内でカチオン性を示す窒素原子をもつ。
3　バレニクリンには鏡像異性体が存在する。
4　ニコチンの不斉炭素は *R* 配置である。
5　ニコチンの sp^2 混成窒素は水素結合受容体として働く。

問 212-213　75 歳女性。骨粗しょう症の治療のため、近隣の整形外科クリニックに通院しており、以下の処方箋を持って薬局を訪れた。

（処方）

リセドロン酸 Na 錠 2.5 mg　1 回 1 錠（1 日 1 錠）
1 日 1 回　起床時　14 日分

この患者は、1 ヶ月前からこの薬剤を継続服用している。薬剤師は患者の医薬品に関する理解度を高めるために、繰り返し、服用に関する注意点を説明することにした。

問 212（実務）

薬剤師が伝えるべき内容として、適切なのはどれか。**２つ**選べ。

1 服用後は、横になって安静にすること。

2 牛乳・乳製品と同時に服用しないこと。

3 服用後すぐに吐き気を催した場合には、制酸剤を服用すること。

4 定期的に歯科検査を受けること。

5 未吸収の成分により便が黒色になるが、心配ないこと。

問 213（物理・化学・生物）

処方薬の化学的性質として、誤っているのはどれか。**1つ**選べ。

1 粘膜刺激性がある。

2 カルシウムイオンなどの金属イオンに対して高い親和性を示す。

3 小腸では高極性のイオン型をとる。

4 ヒドロキシアパタイトに吸着する。

5 塩基性溶液中では加水分解される。

問 214-215　25 歳女性。妊娠なし。最近、便秘気味のため一般用医薬品を求めて薬局を訪れた。薬剤師が症状を確認したところ、ふきでもの（にきび）や食欲不振もみられた。一方、吐き気や腹部の痛みはなかったことから、以下の一般用医薬品を勧めた。

大黄甘草湯エキス顆粒　12 包入り（6 日分）

成分・分量

本品 2 包（3.75 g）中、以下の割合の大黄甘草湯エキス（1/2 量）0.75 g を含有する。

日局ダイオウ・・・・・2.0 g　　　日局カンゾウ・・・・・1.0 g

問 214（物理・化学・生物）

この女性から当該医薬品の成分について聞かれたため、薬剤師は便秘の改善に関係する成分は［ A ］であることを女性に伝えた。［ A ］の化学構造として、適切なのはどれか。**1** つ選べ。

1

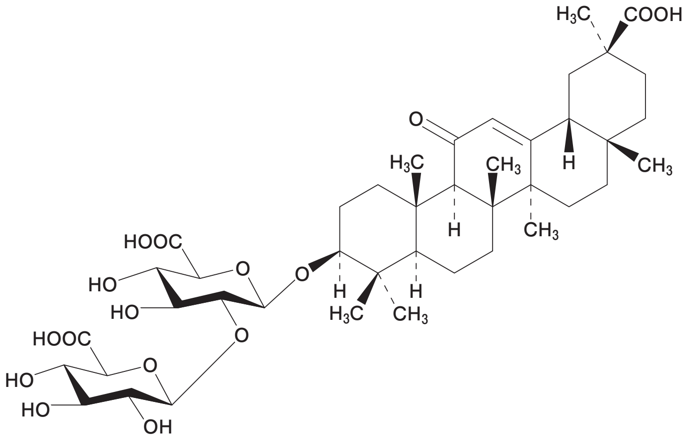

2

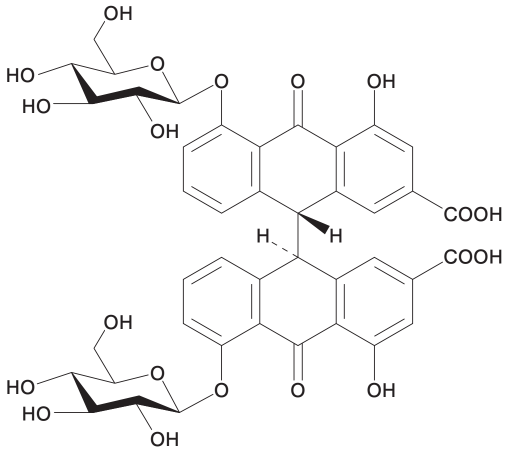

3

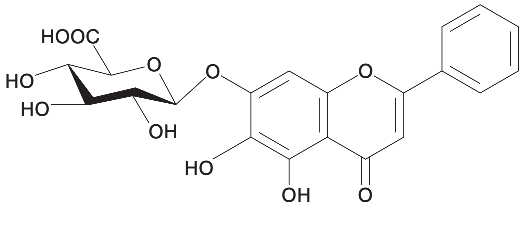

4

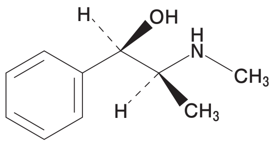

5

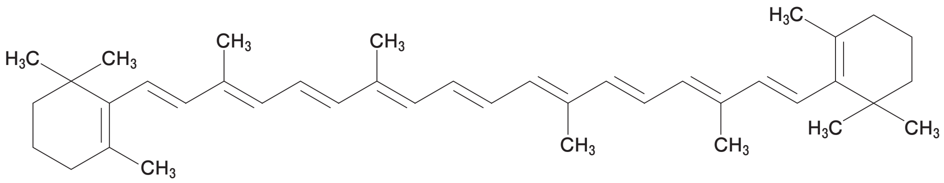

問 215（実務）

薬剤師は、この女性に「主に A によって B が起こることがありますが、心配ありません。」と伝えた。 B に入るものとして、適切なのはどれか。1つ選べ。

1 発疹
2 尿の橙色への着色
3 手足の脱力感
4 筋肉痛
5 激しい腹痛を伴う下痢

問 216-217　38 歳女性。抜け毛が気になり薬局を訪れた。医薬品ではなく、まずはサプリメントで様子をみたいと希望した。薬剤師は、体毛や皮膚を乾燥から守るため脂質成分が重要であることを説明し、ビオチンを含有するサプリメントを紹介した。

問 216（物理・化学・生物）

脂質の生合成に関連する反応のうち、ビオチンが関与するのはどれか。1つ選べ。

1 アセチル CoA からマロニル CoA を合成する反応
2 アシルカルニチンをアシル CoA に変換する反応
3 遊離コレステロールからコレステロールエステルを合成する反応
4 ホスファチジルコリンから脂肪酸を遊離する反応
5 コレステロールから 7-ヒドロキシコレステロールを合成する反応

問 217（実務）

この患者がサプリメントを使用中、過剰に摂取しないように薬剤師から患者に指導すべきものはどれか。1つ選べ。

1 海藻類
2 大豆製品
3 生卵（卵白）
4 牛乳
5 レバー

問 218-219　60 歳女性。右上葉原発性肺腺がんと診断され、右上葉切除術が施行された。その後、術後補助化学療法が施行され経過観察となった。術後 4 年経過時、胸部 CT 写真で右鎖骨上窩リンパ節に転移が認められ、再発と診断された。*ALK* 融合遺伝子陽性が確認されたため、クリゾチニブ 250 mg、1 日 2 回の投与による治療が開始された。投与 13 日目時点でリンパ節の腫瘍は縮小傾向を認めた。各時点における主な検査値は以下のとおりである。

肝機能検査値	投与前	8 日目	13 日目
AST（IU/L）	30	100	350
ALT（IU/L）	30	120	400
ALP（IU/L）	200	600	2,000
総ビリルビン（mg/dL）	1.0	1.1	1.3

問 218（実務）

医師との合同カンファレンスにおいて、医師から薬剤師へ投与 13 日目以降の薬物治療について意見を求められた。薬剤師の提案として、適切なのはどれか。**1つ**選べ。

1　本剤の投与を同一用量のまま継続し、他剤の追加は行わない。
2　本剤の投与を同一用量のまま継続し、グリチルリチン酸一アンモニウム・グリシン・L–システイン塩酸塩水和物を追加する。
3　本剤の投与を中止し、緩和ケアのみの治療へ変更する。
4　本剤の投与を休止し、アレクチニブ塩酸塩へ変更する。
5　本剤の投与を休止し、ソラフェニブトシル酸塩へ変更する。

問 219（物理・化学・生物）

本症例では、遺伝子変異により生じた *ALK* 融合遺伝子及び ALK 融合タンパク質が検出されている。がんとこの遺伝子変異に関する記述として、正しいのはどれか。**2つ**選べ。

1　この患者のがん細胞では、染色体上、*ALK* 遺伝子の一部分に逆位が生じている。
2　この患者の ALK 融合タンパク質では、チロシンキナーゼ活性が亢進している。
3　この患者では、*ALK* 融合遺伝子が親から遺伝したと考えられる。
4　この患者の *ALK* 融合遺伝子は、フィラデルフィア染色体の形成により生じる。
5　*ALK* 融合遺伝子の検出には ELISA 法が用いられる。

問 220-221　35 歳女性。最近、日中頻尿と尿意切迫感で不眠が続いたので近医を受診した。過活動膀胱症状質問票（OABSS）トータルスコア 10 点の中等症と診断され、処方 1 による薬物治療を受けていた。再診時、OABSS トータルスコアは 6 点と改善したが尿意切迫感が十分に改善しないため、処方 2 が追加された。

再診時の主な患者情報：血圧 130/60 mmHg、脈拍数 60 拍/分、消化器症状なし、肝機能・腎機能正常、電解質異常なし。現在、妊娠はしていない。

（処方 1 ）

ソリフェナシンコハク酸塩錠 5 mg	1 回 1 錠（1 日 1 錠）
	1 日 1 回　朝食後　14 日分

（処方 2 ）

ミラベグロン錠 50 mg	1 回 1 錠（1 日 1 錠）
	1 日 1 回　朝食後　14 日分

再診 7 日後、薬剤師が継続的な服薬状況と患者状態を確認し、服薬指導を行うため、患者宅に電話した。

問 220（実務）

副作用症状としてこの患者に起こる可能性が最も低いのはどれか。**1 つ**選べ。

1 血圧上昇
2 尿閉
3 ふらつき、めまい
4 便秘
5 唾液の分泌過多

問 221（物理・化学・生物）

この患者の下部尿路症状を改善させる生理的変化として、正しいのはどれか。**1 つ**選べ。

1 膀胱排尿筋が収縮する。
2 膀胱排尿筋が弛緩する。
3 内尿道括約筋が弛緩する。
4 外尿道括約筋が収縮する。
5 外尿道括約筋が弛緩する。

問 222-223　44 歳女性。鼻、口唇の肥大、下顎の突出を認め精査となった。身長 170 cm、体重 81 kg、靴のサイズ 26.5 cm。75 g ブドウ糖負荷試験での成長ホルモンは 25 ng/mL（正常域 0.4 ng/mL 未満）、IGF-1（血中インスリン様成長因子-1）は 1,050 ng/mL（正常域 88〜229 ng/mL）であった。MRI 検査で限局性腫瘤が認められたが、異所性病変は認めなかった。

問 222（物理・化学・生物）

図は、女性の内分泌器官を表した模式図である。この患者の腫瘤の位置はどれか。**1つ**選べ。

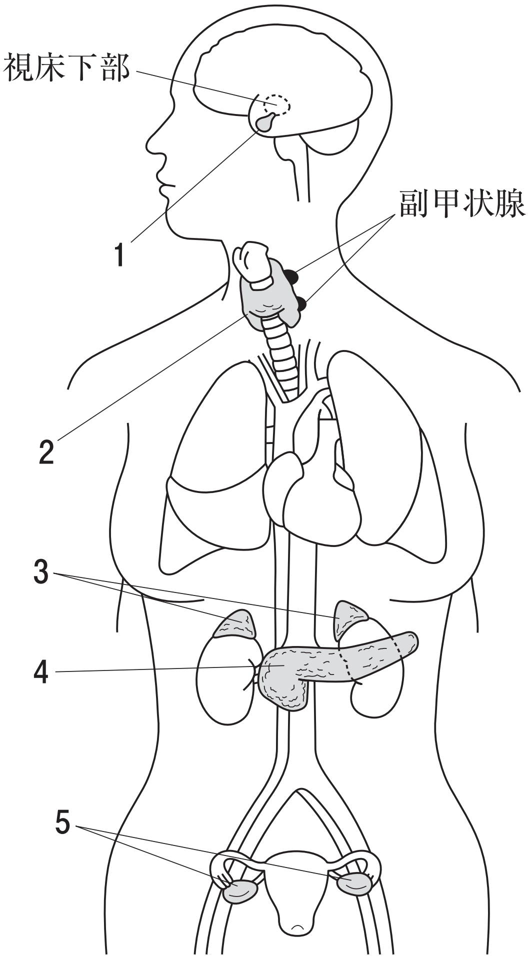

問 223（実務）

この患者は、その後、精密検査の結果、悪性腫瘍と診断された。腫瘍が大きく手術が困難であるため、薬物治療を行う方針となった。なお、放射線治療は薬物治療の効果をみてから検討する予定である。この患者の治療に用いる薬物の候補として、最も適切なのはどれか。**1つ**選べ。

1 グルカゴン
2 インスリン
3 オクトレオチド
4 ソマトロピン
5 バソプレシン

問 224-225　65 歳女性。体重 50 kg。術後肺炎と診断され、喀痰から緑膿菌が検出された。医師の指示により、シプロフロキサシン注射液が静脈内投与された。

問 224（物理・化学・生物）

検査部で、患者の痰から分離・同定した緑膿菌を培養し、薬剤感受性試験としてディスク法を実施した。

ディスク法の説明

寒天培地に一定量の菌を均一に広げた後、上にディスク（一定量の抗菌薬を染み込ませたろ紙）を置いて培養する方法（図 1 参照）。ディスクから培地に拡散した抗菌薬によって菌の発育阻止円ができ、その直径を測定する。

多剤耐性緑膿菌の場合、判定に用いる抗菌薬（β-ラクタム系、フルオロキノロン系及びアミノ配糖体系の 3 系統）の種類と判定に適した濃度は決められており、指定の条件で一定時間培養後、生じた阻止円の直径をもとに、感受性か耐性かを判断する。

図 1

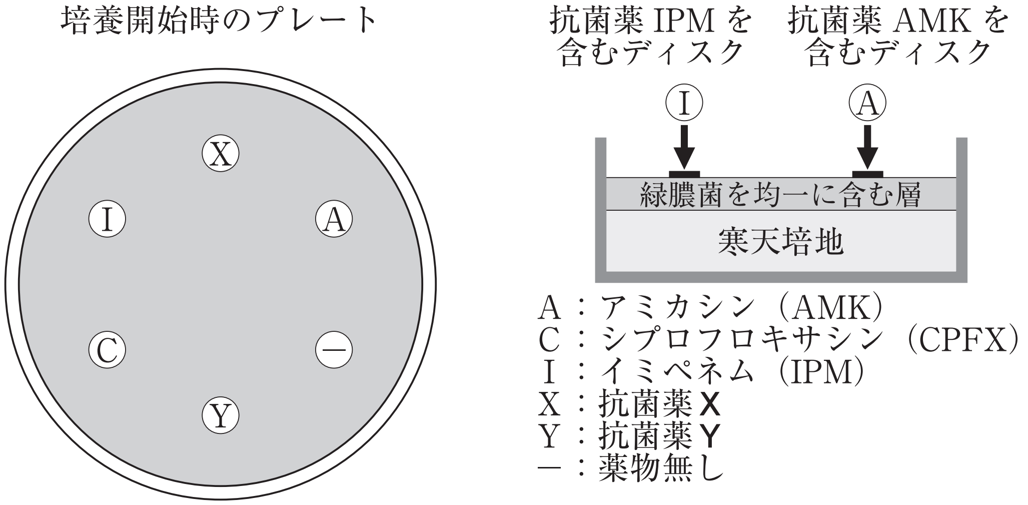

今回は、この患者由来の緑膿菌と通常の緑膿菌基準株を用いた。また、指定されたイミペネム（IPM）、シプロフロキサシン（CPFX）、アミカシン（AMK）の 3 剤に加え、抗菌薬 **X** と **Y** も調べた。その結果を図 2 に示す。

図 2

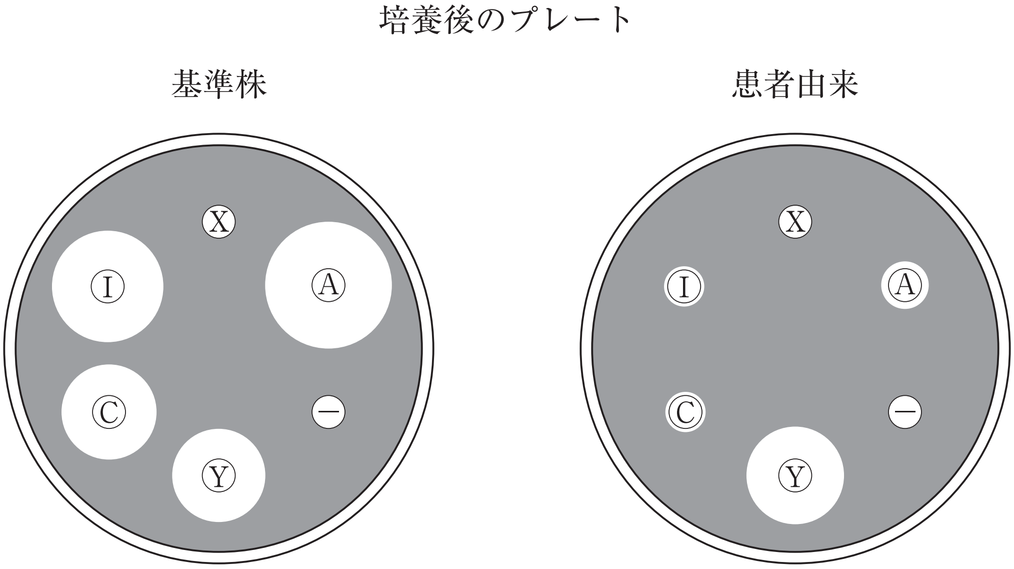

本試験とその結果に関する記述のうち、正しいのはどれか。<u>**2つ**</u>選べ。

1　本法は、微量液体希釈法よりも最小発育阻止濃度（MIC）を算出するのに適している。

2　阻止円の直径が大きいほど、その抗菌薬への感受性が高い。

3　この患者より単離した緑膿菌は、多剤耐性菌である。

4　抗菌薬**X**は、この患者の治療薬候補になる。

5　抗菌薬**Y**は、この患者の治療薬候補にはならない。

問 225（実務）

薬剤感受性試験結果から考えられる、この患者に最も適切な抗菌薬療法はどれか。**1つ**選べ。なお、薬剤はすべて注射剤である。

1　シプロフロキサシンを基本とした併用療法への変更

2　アルベカシン硫酸塩の単独療法への変更

3　ピペラシリンナトリウムの単独療法への変更

4　シプロフロキサシンの単独療法の継続

5　コリスチンメタンスルホン酸ナトリウムを基本とした併用療法への変更

問 226-227　早期の大腸がんは症状がないことが多く、便潜血検査が早期発見に有効であることが知られている。以下の表は、免疫学的便潜血検査による大腸がんのスクリーニング結果を示したものである。

	大腸がん		合計（人）
	あり	なし	
検査で陽性（人）	32	761	793
検査で陰性（人）	8	8669	8677
合計（人）	40	9430	9470

問 226（実務）

この検査法での感度と特異度の組合せとして、正しいのはどれか。**1つ**選べ。

	感度（%）	特異度（%）
**1**	80.0	91.9
**2**	80.0	99.9
**3**	91.9	80.0
**4**	96.0	91.9
**5**	99.9	80.0

問 227（衛生）

図は、大腸がんの多段階発がんの過程を示したものである。A 及び B に該当するがん抑制遺伝子の組合せとして、正しいのはどれか。**1 つ**選べ。

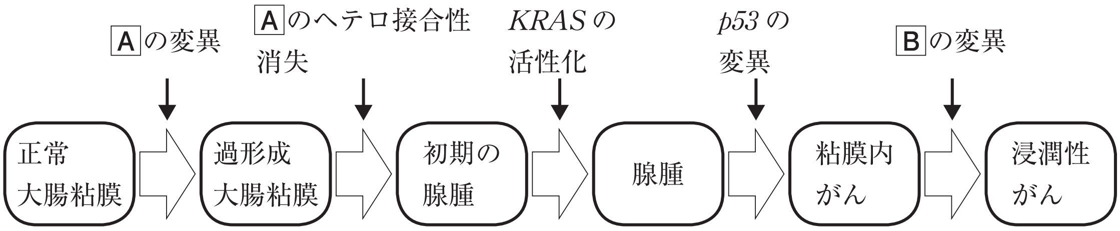

	A	B
1	*BRCA1*	*WT1*
2	*BRCA1*	*DCC*
3	*MYC*	*MLH1*
4	*APC*	*PTEN*
5	*APC*	*DCC*

問 228-229　70 歳男性。自宅にて、39 ℃の発熱及び全身倦怠感を認め、同日中に呼吸困難となったため、家族が救急搬送を依頼した。救急病院に到着後、インフルエンザウイルスの迅速抗原検出キットにて検査したところ、B 型陽性であり、インフルエンザウイルス感染症と診断された。なお、インフルエンザワクチンは未接種だった。また、本人からは高熱による頭痛の訴えがあった。救命救急センター担当医師と薬剤師は、治療方針について、カンファレンスを実施した。

問 228（実務）

この患者への対応について、薬剤師が提案する内容として、適切なのはどれか。
２つ選べ。

1　レボフロキサシン水和物錠の投与
2　インフルエンザワクチンの接種
3　アセトアミノフェン静注液の投与
4　ペラミビル水和物注射液の投与
5　アマンタジン塩酸塩錠の投与

問 229（衛生）

この患者の家族から、今後のインフルエンザワクチン接種について薬剤師に質問があった。次の記述のうち、正しいのはどれか。**2つ**選べ。

1　この患者が予防接種を受ける場合、インフルエンザは予防接種法における A 類疾病に分類される。

2　この患者に対するインフルエンザワクチンの接種にかかる費用は、公的補助の対象とはならない。

3　インフルエンザワクチンの接種においては、鶏卵、鶏肉、その他鶏由来のものに対してアレルギーがある場合には注意が必要である。

4　インフルエンザワクチンの接種後、この患者に健康被害が生じた場合は、予防接種法に基づいた予防接種健康被害救済制度により救済措置を受けることができる。

5　インフルエンザワクチンは弱毒化ワクチンなので、ワクチン接種によりインフルエンザを発症することがある。

問 230-231　43 歳男性。身長 170 cm、体重 85 kg、喫煙歴なし。運動不足であり、食事については特に気にせず、油ものを好んでいた。今回、妻と一緒に近隣で開催の健康フェアに行き、健康相談コーナーで薬剤師に今後必要な生活習慣について相談した。その際、勤務先で実施した特定健康診査結果を持参していた。なお、これまでに健診で生活習慣の改善を指摘されていたが受診歴はなく、現在も服用薬はない。

（検査結果）

腹囲 95 cm、血圧 142/87 mmHg、HDL-C 38 mg/dL、
中性脂肪 160 mg/dL、空腹時血糖 93 mg/dL、HbA1c 5.2%（NGSP 値）

問 230（実務）

特定健康診査で、この男性は特定保健指導の対象になった。その原因となった検査項目として、誤っているのはどれか。**1つ選べ。**

1 HDL-C
2 腹囲
3 血圧
4 空腹時血糖
5 中性脂肪

問 231（衛生）

この男性に対して行われた特定健康診査における階層化として、正しいのはどれか。**1つ選べ。**

	腹囲/BMI		追加リスク		保健指導レベル
1	腹囲 基準値以上	→	1つ該当	→	積極的支援
2	腹囲 基準値以下<br>BMI ≧ 25 kg/m^2	→	3つ該当	→	積極的支援
3	腹囲 基準値以上	→	2つ該当	→	積極的支援
4	腹囲 基準値以下<br>BMI ≧ 25 kg/m^2	→	2つ該当	→	動機付け支援
5	腹囲 基準値以上	→	2つ該当	→	動機付け支援

問 232-233　39 歳女性。今回初めて妊娠した。8 週目の妊婦健診で B 型肝炎の検査を実施したところ、HBs 抗原が陽性であった。

問 232（実務）

この妊婦への対応として、適切なのはどれか。**1つ**選べ。

1　HBe 抗原を検査する。
2　B 型肝炎ワクチンを接種する。
3　速やかに、抗 HBs 人免疫グロブリンを投与する。
4　エンテカビルを投与する。
5　出産まで、特に対応の必要はない。

問 233（衛生）

この妊婦から出生した児に対して行うＢ型肝炎ウイルス感染に関する予防処置を時系列で示した（A～C）。正しい組合せはどれか。1つ選べ。

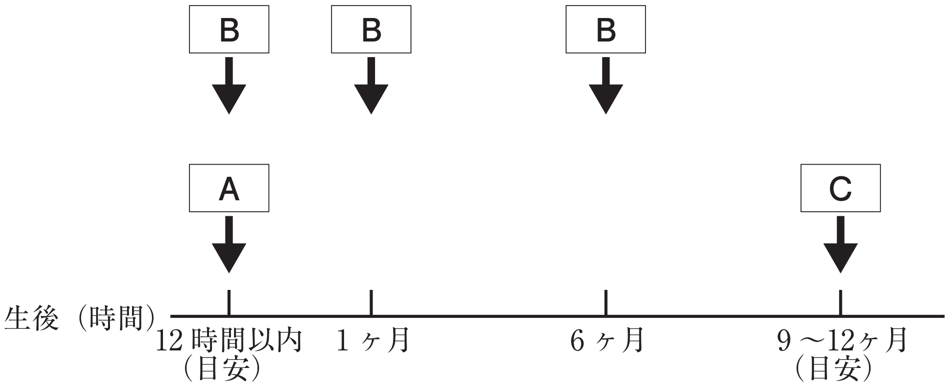

	A	B	C
1	HBs 抗原・抗体検査	B 型肝炎ワクチン接種	抗 HBs 人免疫グロブリン投与
2	HBs 抗原・抗体検査	抗 HBs 人免疫グロブリン投与	B 型肝炎ワクチン接種
3	抗 HBs 人免疫グロブリン投与	HBs 抗原・抗体検査	B 型肝炎ワクチン接種
4	抗 HBs 人免疫グロブリン投与	B 型肝炎ワクチン接種	HBs 抗原・抗体検査
5	B 型肝炎ワクチン接種	抗 HBs 人免疫グロブリン投与	HBs 抗原・抗体検査

問 234-235　74 歳女性。身長 160 cm、体重 50 kg。飲酒及び喫煙歴はない。てんかんの既往があり、以下の薬剤を 10 年以上服用し、外来受診時には脳波検査を行ってきた。１年以上、発作は起こっていない。今回、市が主催する骨密度検診で、骨密度の低下が指摘されたので、かかりつけの医療機関を受診し血液検査と骨密度測定を実施した。その結果をもとに、医師と薬剤師がカンファレンスを行った。

（処方）

フェニトイン錠 100 mg　1 回 1 錠（１日３錠）
1 日 3 回　朝昼夕食後　28 日分

（検査値）

血清クレアチニン値 1.6 mg/dL、AST 32 IU/L、ALT 29 IU/L
ALP 410 IU/L、補正 Ca 値 7.0 mg/dL
intact-PTH 92 pg/mL（標準値：10〜65 pg/mL）
フェニトイン血中濃度 10 μg/mL
腰椎骨密度測定値 若年成人平均値（YAM）の 65％

問 234（衛生）

この患者の病態に関連するビタミンやミネラルについての記述のうち、正しいのはどれか。2つ選べ。

1 intact-PTH が高値を示していることから、血中カルシウム濃度を正常に維持するために副甲状腺の機能が亢進していることがわかる。

2 血清カルシウム値の低下は、活性型ビタミン D によるカルシウム吸収促進能が低下したことによるものである。

3 副甲状腺の機能が亢進したことにより、腎臓でのカルシウム排泄が促進され、血清カルシウム値が低下している。

4 ビタミン K を含む食品を摂取することで、腸管からのカルシウム吸収を促進することができる。

5 ホウレンソウなどに含まれるフィチン酸と一緒にカルシウムを摂取することで、効率よくカルシウムを吸収することができる。

問 235（実務）

薬剤師が医師に処方提案する薬剤として、適切なのはどれか。1つ選べ。

1 エルカトニン注

2 アルファカルシドールカプセル

3 イバンドロン酸ナトリウム水和物注

4 アレンドロン酸ナトリウム水和物錠

5 デノスマブ皮下注

問 236-237　68 歳男性。身長 168 cm、体重 58 kg。6 年前に胃の全摘手術を受けている。1 週間前から息切れと全身倦怠感を感じていた。今回、著しい食欲不振のため食事が摂れなくなり、めまいも伴うので外来を受診した。血液検査の結果は、以下のとおりである。

（検査値）

BUN 11.1 mg/dL、血清クレアチニン値 0.6 mg/dL、
赤血球数 221 × 10^4/μL、Hb 9.7 g/dL、MCV 122.2 fl、MCH 43.9 pg、
MCHC 35.9%、血清鉄 101 μg/dL、フェリチン 56 μg/dL、
PT-INR 1.10、便潜血　陰性

問 236（実務）

この患者において、欠乏が疑われるビタミンはどれか。**1つ**選べ。

1　ビタミン B_1
2　ビタミン B_6
3　ビタミン B_{12}
4　ビタミンＣ
5　ビタミンＫ

問 237（衛生）

この患者に欠乏していると考えられるビタミンに関する記述のうち、正しいのはどれか。**2つ**選べ。

1　このビタミンの欠乏の原因の一つに、動物性食品の摂取不足があげられる。
2　このビタミンは、プロリンやリシンの水酸化酵素の補酵素としてコラーゲン合成に関与する。
3　このビタミンは、唾液由来のトランスコバラミンとの複合体として、小腸から吸収される。
4　このビタミンは、葉酸代謝におけるメチル基転位反応に関与する。
5　このビタミンは、生体内でチアミンピロリン酸として糖代謝に関与する。

問 238-239　10 月 14 日（月曜日）に小学校において、50 名の児童が発熱・嘔吐・下痢の症状で欠席し、翌日にも同様の症状でさらに 65 名が欠席し児童の多くが病院を受診しているとの連絡が保健所にあった。早速、これらの患者のうち、60 名の検体について検査を行ったところ、48 名の検体から同一の病因物質を検出した。患者らの共通食は学校給食のみであり、10 月 11 日（金曜日）に遠足のために給食を食べなかった学年に有症者がいないことから、給食が食中毒の原因と断定した。なお、衛生検査用に冷凍保存されていた同じ給食を調べた結果、原材料の鶏肉からも同じ病因物質を検出した。これを顕微鏡で観察したところ、写真の様に細長い、らせん状の形態を示していた。

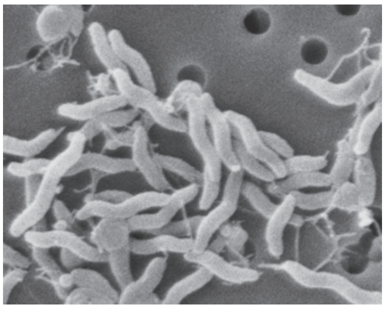

病因物質の顕微鏡像

問 238（衛生）

下図は、病因物質（A～E）による食中毒の患者数と事件数の年次別推移を示したものである。この給食による食中毒の病因物質はどれか。１つ選べ。

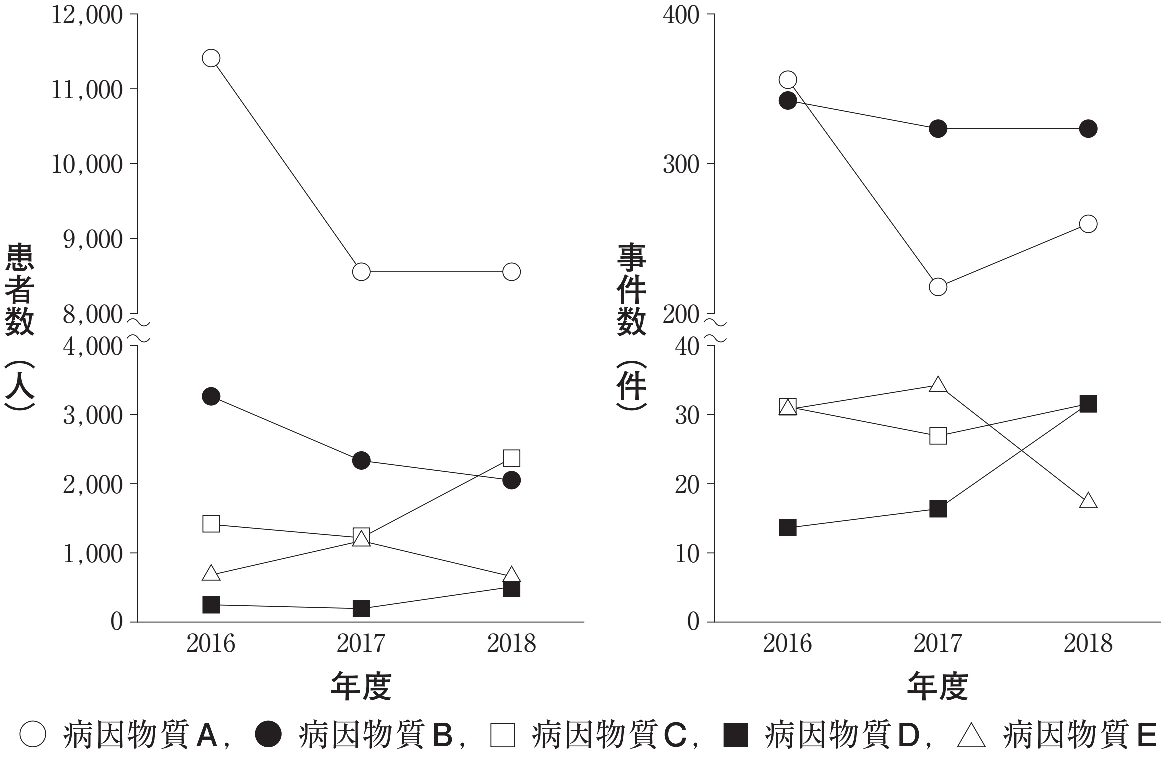

1　病因物質A

2　病因物質B

3　病因物質C

4　病因物質D

5　病因物質E

問 239（実務）

今回、病院を受診した患者の一部には、重篤な食中毒症状がみられた。その患者に投与すべき薬剤として、適切なのはどれか。１つ選べ。

1　ロペラミド塩酸塩カプセル

2　５％ブドウ糖加酢酸リンゲル液

3　アトロピン硫酸塩注射液

4　*d*-クロルフェニラミンマレイン酸塩注射液

5　ブチルスコポラミン臭化物注射液

問 240-241　12 月に行われた少年スポーツクラブの大会で、金属製のやかんを使って、粉末のスポーツドリンクを水道水で溶かし、そのスポーツドリンクを飲んだ子供たちが吐き気や嘔吐を発症した。8 人の患者が近医を受診し、血液検査及び便検査を行った。血液検査の結果、血清中の亜鉛濃度 90〜101 μg/dL（正常値；80〜130 μg/dL)、銅濃度 278〜314 μg/dL（正常値；70〜132 μg/dL)、セレン濃度 11.2〜13.2 μg/dL（正常値；10.6〜17.4 μg/dL）の範囲であり、他の重金属や中毒物質は検出されなかった。また、便検査の結果、食中毒の原因と考えられる細菌及びウイルスは検出されなかった。

　一方、後日、やかんに残っていたスポーツドリンクを衛生研究所で分析したところ、以下のようなイオンが検出された。

ナトリウム 520 mg/L、カリウム 214 mg/L、カルシウム 22 mg/L、
マグネシウム 6 mg/L、銅 200 mg/L

問 240（実務）

これらの患者に対する治療薬として、適切なのはどれか。**1つ**選べ。

1　イダルシズマブ注射液
2　ナロキソン塩酸塩注射液
3　ペニシラミンカプセル
4　フルマゼニル注射液
5　プラリドキシムヨウ化物注射液

問 241（衛生）

この中毒の原因となった物質の解毒にはたらく生体分子はどれか。**1つ**選べ。

1　カタラーゼ
2　グルタチオンペルオキシダーゼ
3　シトクロム P450
4　メタロチオネイン
5　ビリルビン

問 242-243　連日、猛暑のために熱中症警戒アラートが発表されている。そこで、高校の体育教員が経口補水液を買いに薬局に来て、薬剤師に熱中症や暑さ指数（WBGT）について質問をした。

問 242（実務）

熱中症や暑さ指数（WBGT）に関する説明として、誤っているのはどれか。**1つ**選べ。

1　熱中症は、体内の水分や電解質が欠乏することで起こる健康障害です。

2　熱中症は、屋内でも起こることがあるので、特に厚手の衣類を着用するスポーツでは注意が必要です。

3　同じ気温でも、湿度が高いときほど熱中症の危険性は高くなります。

4　暑さ指数（WBGT）は、熱中症を予防することを目的として提案された指標で、℃の単位で表されます。

5　暑さ指数（WBGT）は、感覚温度図表を用いて算出されます。

問 243（衛生）

熱中症及び暑さ指数（WBGT）について説明したところ、「近々開催する運動会の当日に暑さ指数を測定したいので、必要な器具を紹介して欲しい」との依頼があった。暑さ指数（WBGT）を求めるために必要な測定器具はどれか。**２つ**選べ。

1

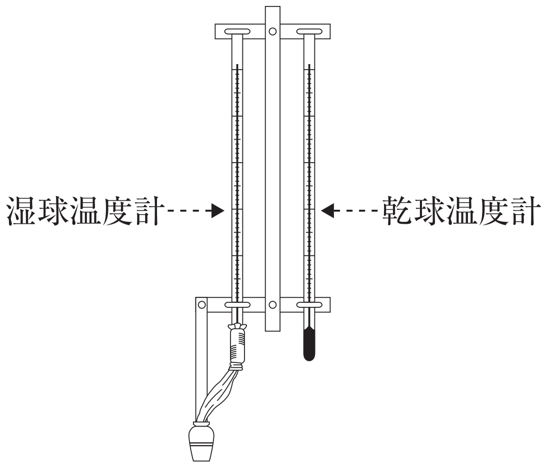

2

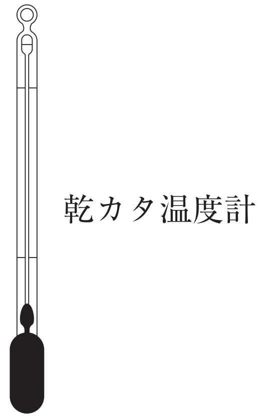

3

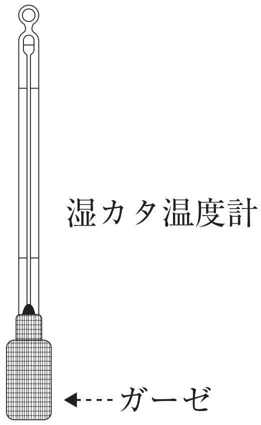

4

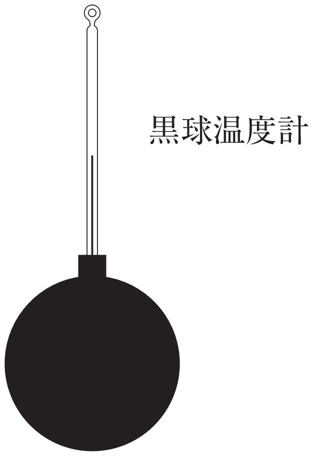

5

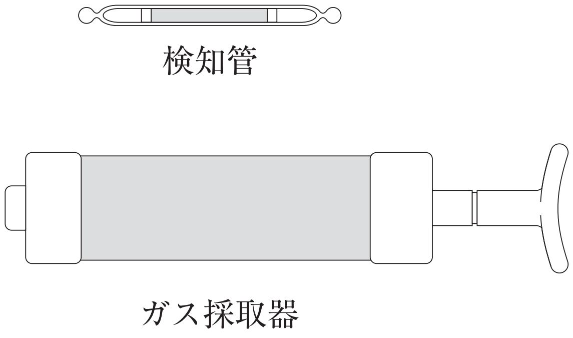

問 244-245　校舎が老朽化したため、一部の教室の改築が行われた。改築した教室を利用した生徒から、目、鼻、のどの刺激、めまいの訴えが続いたため、養護教諭から学校薬剤師に相談があった。学校薬剤師がこの教室内の空気中の化学物質を検査したところ、「学校環境衛生基準」で定められている 2 つの物質が高濃度で検出された。

問 244（実務）

生徒の症状の原因と考えられる物質の組合せとして、正しいのはどれか。**1 つ**選べ。

	原因物質 1	原因物質 2
1	アスベスト	フタル酸ジ-2-エチルヘキシル
2	フタル酸ジ-2-エチルヘキシル	ホルムアルデヒド
3	ホルムアルデヒド	キシレン
4	キシレン	一酸化炭素
5	一酸化炭素	アスベスト

問 245（衛生）

前問で選択した原因物質 1 及び原因物質 2 を測定するための試験法の組合せとして、正しいのはどれか。**1 つ**選べ。

	原因物質 1	原因物質 2
1	ガスクロマトグラフ ― 質量分析法	酵素免疫測定法
2	検知管法	ザルツマン法
3	検知管法	酵素免疫測定法
4	ジニトロフェニルヒドラジン誘導体化法を用いた高速液体クロマトグラフ法	ザルツマン法
5	ジニトロフェニルヒドラジン誘導体化法を用いた高速液体クロマトグラフ法	ガスクロマトグラフ ― 質量分析法

107

2日目②　　一般問題（薬学実践問題）

【薬理、薬剤／実務】

◎指示があるまで開いてはいけません。

注　意　事　項

1　試験問題の数は、**問２４６**から**問２８５**までの**４０問**。
１３時から**１４時４０分**までの**１００分以内**で解答すること。

2　解答方法は次のとおりである。

(1)　一般問題（薬学実践問題）の各問題の正答数は、**問題文中に指示されている**。
問題の選択肢の中から答えを選び、次の例にならって答案用紙に記入すること。
なお、問題文中に指示された正答数と**異なる数を解答すると、誤りになる**から注意すること。

（例）**問 500**　次の物質中、常温かつ常圧下で液体のものはどれか。**２つ**選べ。

1　塩化ナトリウム　　2　プロパン　　3　ベンゼン
4　エタノール　　5　炭酸カルシウム

正しい答えは「3」と「4」であるから、答案用紙の

問 500　1 2 3 4 5 6 7 8 9 10　のうち　3　と　4　を塗りつぶして

問 500　1 2 3 4 5 6 7 8 9 10　とすればよい。

(2)　解答は、◯の中全体をＨＢの鉛筆で濃く塗りつぶすこと。塗りつぶしが薄い場合は、解答したことにならないから注意すること。

悪い解答例　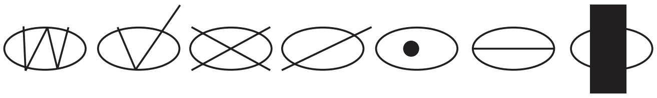　**（採点されない）**

(3)　解答を修正する場合は、必ず「消しゴム」で跡が残らないように完全に消すこと。鉛筆の跡が残ったり、「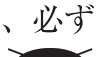」のような消し方などをした場合は、修正又は解答したことにならないから注意すること。

(4)　答案用紙は、折り曲げたり汚したりしないよう、特に注意すること。

3　設問中の科学用語そのものやその外国語表示（化合物名、人名、学名など）には誤りはないものとして解答すること。ただし、設問が科学用語そのもの又は外国語の意味の正誤の判断を求めている場合を除く。

4　問題の内容については質問しないこと。

一般問題（薬学実践問題）【薬理、薬剤／実務】

問 246-247　23 歳女性。半年前に幻覚と妄想が出現し統合失調症と診断され、リスペリドン 6 mg による治療を受けていた。精神症状は改善したが、手のふるえや筋肉が突っ張るような錐体外路症状が出現した。また、これまで規則正しかった月経が止まったとの訴えがあり、検査により高プロラクチン血症と診断された。患者から別の薬剤への変更を希望され、以下の処方へ変更することになった。

（処方）

アリピプラゾール錠 12 mg　1 回 1 錠（1 日 1 錠）
1 日 1 回　朝食後　14 日分

問 246（薬理）

この患者でリスペリドン服用中に認められた副作用発生の主な機序として、正しいのはどれか。２つ選べ。

1 線条体におけるドパミン D_2 受容体遮断
2 線条体におけるセロトニン 5-HT_{2A} 受容体遮断
3 中脳辺縁系におけるドパミン D_2 受容体遮断
4 中脳辺縁系におけるセロトニン 5-HT_{2A} 受容体遮断
5 脳下垂体前葉におけるドパミン D_2 受容体遮断

問 247（実務）

変更後の処方薬に関する記述として、適切なのはどれか。２つ選べ。

1 第 1 世代（定型）の統合失調症治療薬と比べ錐体外路症状が発現しにくい。
2 変更後 2 ヶ月間は、好中球数のモニタリングが毎週必要である。
3 分布容積が大きいため、過量投与に対する処置として血液透析が有効である。
4 投与中は血糖値の定期的なモニタリングが必要である。
5 薬物治療は今後 1 ヶ月で終了することが見込まれる。

問 248-249　68 歳男性。体重 62 kg。最近動悸が激しく息切れすることもあったが放置していた。突然、左側の手足のしびれや麻痺が発現し、言葉も出てこなくなったため、家族が救急車を要請し緊急入院となり、心房細動及び心原性脳梗塞と診断された。心不全の症状はなく、その後の治療により病状が落ち着いたため退院することになった。入院中の処方 1 に加え、退院時に処方 2 が新たに追加されることになった。また、現在の検査値は以下のとおりである。

（検査値）

血圧 140/88 mmHg、心拍数 110 拍/分、BUN 28 mg/dL、血清クレアチニン値 1.4 mg/dL、クレアチニンクリアランス 42 mL/min、LDL 165 mg/dL、HDL 50 mg/dL、TG（トリグリセリド）140 mg/dL

（処方 1 ）

カルベジロール錠 2.5 mg　1 回 2 錠（1 日 2 錠）
1 日 1 回　朝食後　14 日分

（処方 2 ）

エドキサバントシル酸塩水和物口腔内崩壊錠 60 mg　1 回 1 錠（1 日 1 錠）
1 日 1 回　朝食後　14 日分

問 248（薬理）

処方１及び２のいずれかの薬物の作用機序として、適切なのはどれか。**1つ**選べ。

1 アデノシン $P2Y_{12}$ 受容体を遮断して、血小板凝集を抑制する。

2 ビタミン K の代謝サイクルを阻害して、血液凝固を阻害する。

3 第 Xa 因子を阻害して、トロンビン産生を抑制する。

4 トロンボキサン A_2 の合成を阻害して、血小板の活性化を阻害する。

5 プラスミノーゲンをプラスミンに変換して、血栓中のフィブリンを分解する。

問 249（実務）

処方１と２を監査した病棟薬剤師が処方医に提案する内容として、適切なのはどれか。**2つ**選べ。

1 カルベジロールを減量する。

2 エドキサバンを減量する。

3 アスピリンを追加する。

4 アトルバスタチンを追加する。

5 クロピドグレルを追加する。

問 250-251　64 歳女性。8 年前に朝の手指のこわばり、多関節痛が出現し、近医にて関節リウマチと診断された。メトトレキサート（6 mg/週）でコントロールされていたが、1 ヶ月前より関節症状が悪化したため入院した。入院時検査では、腫脹関節10ヶ所、圧痛関節 6 ヶ所、赤血球沈降速度 112 mm/h、CRP 8.5 mg/dL であったことから疾患活動性が高いと判断され、インフリキシマブ（3 mg/kg）を併用することになった。なお、体温 37.2 ℃、血圧 94/58 mmHg、脈拍数 80 拍/分、ALT 80 IU/L、AST 88 IU/L であった。

問 250（実務）

この患者の治療における薬剤師の対応として、誤っているのはどれか。**1 つ**選べ。

1　入院時検査で軽度肝機能障害が認められるので、ホリナートカルシウム錠の投与を医師に提案した。

2　この患者の B 型肝炎ウイルス感染の有無を確認した。

3　この患者の結核の既往の有無を確認した。

4　インフリキシマブ投与により infusion reaction が認められた場合は、メトトレキサートの増量が必要と医師に情報提供した。

5　咳や喉の痛み等風邪の症状が出た場合は、すぐに医師、看護師、薬剤師等に連絡するように患者に伝えた。

問 251（薬理）

メトトレキサートとインフリキシマブを投与した場合、この患者で期待される効果の機序はどれか。２つ選べ。

1　TNF-α の作用が阻害され、関節滑膜細胞の増殖が抑制される。

2　ジヒドロ葉酸還元酵素の阻害によりリンパ球の増殖が抑制され、中和抗体の産生が抑制される。

3　シクロオキシゲナーゼの阻害によりプロスタグランジン合成が抑制され、疼痛が緩和される。

4　IL-6 受容体が遮断され、破骨細胞の分化が抑制される。

5　炎症局所のアデノシン受容体が遮断され、炎症反応が抑制される。

問 252-253　62 歳男性。3 年前、階段を昇る時に息切れを感じるようになり受診したところ、左室肥大と肺うっ血を認め、慢性心不全と診断された。処方 1 ～処方 3 で治療されていたが、慢性心不全の増悪により入院した。その後、処方 4 を追加して病態が安定したため、退院することになった。現在の検査値等は以下のとおりである。

（検査値）

血圧 120/82 mmHg、心拍数 84 拍/分、AST 24 IU/L、ALT 16 IU/L、
BUN 18 mg/dL、血清クレアチニン値 0.9 mg/dL、Na 145 mEq/L、
K 2.9 mEq/L、Cl 102 mEq/L、血清 BNP 410 pg/mL、左室駆出率 EF 33％

（処方 1 ）

エナラプリルマレイン酸塩錠 10 mg	1 回 1 錠（1 日 1 錠）
	1 日 1 回　朝食後　30 日分

（処方 2 ）

ビソプロロールフマル酸塩錠 2.5 mg	1 回 1 錠（1 日 1 錠）
	1 日 1 回　朝食後　30 日分

（処方 3 ）

フロセミド錠 40 mg	1 回 1 錠（1 日 1 錠）
	1 日 1 回　朝食後　30 日分

（処方 4 ）

エプレレノン錠 25 mg	1 回 1 錠（1 日 1 錠）
	1 日 1 回　朝食後　30 日分

問 252（実務）

この患者に対する副作用モニタリングとして、適切なのはどれか。**2つ**選べ。

1　腎機能検査値は基準値内と判断する。

2　血清電解質（Na、K、Cl）値は、いずれも基準値内と判断する。

3　徐脈と判断する。

4　今後、処方3の薬剤による血清ナトリウム値の上昇に注意する。

5　今後、処方1や処方4の薬剤により血清カリウム値が上昇しすぎないか注意する。

問 253（薬理）

この患者に追加された処方4の薬物の作用として、適切なのはどれか。**1つ**選べ。

1　心臓のアドレナリン β_1 受容体を遮断して、BNP値を低下させる。

2　アンジオテンシン変換酵素を阻害して、心筋の線維化を抑制する。

3　心筋に直接作用して心収縮力を高めて、左室駆出率を改善する。

4　ヘンレ係蹄上行脚において Na^+ と Cl^- の再吸収を抑制して、むくみを改善する。

5　遠位尿細管及び集合管においてアルドステロン受容体を遮断して、尿中への K^+ の排泄を抑制する。

問 254-255　45 歳男性。体重 45 kg。10 年前に全結腸型潰瘍性大腸炎と診断され、寛解・再燃を繰り返した後、メサラジン 1,500 mg/日、アザチオプリン 50 mg/日で寛解維持されていた。2 ヶ月前より大腸炎が再燃し、上の処方で効果不十分であったため、以下の処方にて寛解導入することになった。

（処方 1）

タクロリムスカプセル 1 mg　1 回 1 カプセル（1 日 2 カプセル）
1 日 2 回　朝夕食後　3 日分

（処方 2）

アダリムマブ（遺伝子組換え）皮下注 80 mg ペン 0.8 mL　1 回 160 mg
2 本（1 回分）

問 254（実務）

薬剤師のこの患者への説明として、適切なのはどれか。2つ選べ。

1　処方１の薬剤は血中濃度を測定しながら服用カプセル数を調節します。

2　処方１の薬剤は腎障害が起こりやすいので、尿量の減少などがあれば薬剤師に相談してください。

3　処方１の薬剤は低血糖になりやすいので、異常な空腹感や冷や汗、動悸があるときはすぐに糖分を摂取してください。

4　処方２の薬剤は今回のみの使用で終了します。

5　処方２の薬剤の使用直後に、まれにふらつきや息苦しさを感じることがありますが、しばらく安静にすると自然に治まります。

問 255（薬理）

処方１及び２のいずれかの薬物に期待される効果の機序はどれか。2つ選べ。

1　TNF-α に結合して、TNF-α とその受容体の結合を阻害する。

2　ヤヌスキナーゼ（JAK）を阻害して、サイトカイン受容体を介した細胞内情報伝達を抑制する。

3　ロイコトリエンの産生を阻害して、白血球の組織への浸潤を抑制する。

4　プリン塩基の合成を阻害して、リンパ球の増殖を抑制する。

5　カルシニューリンを阻害して、T 細胞における IL-2 などのサイトカイン産生を抑制する。

問 256-257　70 歳女性。高血圧、心筋梗塞の既往あり。処方１～処方３の薬剤を服用していたが、脂質異常症の治療効果不十分のため処方４が追加された。現在の身体所見等は以下のとおりである。

身体所見：身長 155 cm、体重 56 kg、血圧 118/75 mmHg、脈拍数 67 拍/分（整）
血液検査：AST 30 IU/L、ALT 28 IU/L、血清クレアチニン値 0.75 mg/dL、BUN 17 mg/dL、HDL 42 mg/dL、LDL 122 mg/dL、TG（トリグリセリド）110 mg/dL

（処方１）
アスピリン腸溶錠 100 mg　　1回1錠（1日1錠）
ラベプラゾール Na 錠 5 mg　　1回1錠（1日1錠）
　　1日1回　朝食後　7日分

（処方２）
テルミサルタン 40 mg/アムロジピンベシル酸塩 5 mg 配合錠
　　1回1錠（1日1錠）
　　1日1回　朝食後　7日分

（処方３）
ロスバスタチン口腔内崩壊錠 10 mg　　1回2錠（1日2錠）
　　1日1回　朝食後　7日分

（処方４）
エゼチミブ錠 10 mg　　1回1錠（1日1錠）
　　1日1回　朝食後　7日分

問 256（薬理）

処方１～処方４のいずれかの薬物の作用機序に関する記述のうち、正しいのはどれか。**２つ**選べ。

1　胆汁酸を吸着して小腸での再吸収を阻害して、コレステロールの胆汁酸への異化を促進する。
2　アンジオテンシン変換酵素を阻害して、ブラジキニンの分解を抑制する。
3　電位依存性Ｌ型 Ca^{2+} チャネルを遮断して、細動脈を拡張させる。
4　ミクロソームトリグリセリド転送タンパク質（MTP）に結合して、アポタンパク質Ｂへのトリグリセリドの転送を阻害する。
5　シクロオキシゲナーゼを阻害して、血小板におけるトロンボキサン A_2 産生を抑制する。

問 257（実務）

この患者の脂質異常症の治療に関して、適切なのはどれか。**１つ**選べ。

1　HDL の目標値は 45 mg/dL 未満である。
2　LDL の管理目標値は、既往歴のない患者の目標値（140 mg/dL）より低く設定されている。
3　TG が管理目標値以上であるため、ベザフィブラートの追加が必要である。
4　処方３の薬剤の吸収が低下するため、処方４は服用時間を夕食後に変更する。
5　処方４の薬剤の追加で十分な効果が得られない場合は、プロブコールをさらに併用する。

問 258-259　33歳女性。最近体重が減少し、手指振戦、動悸、多汗があるため受診した。身体所見として眼球突出、びまん性甲状腺腫がありバセドウ病と診断され、薬局に以下の処方箋を持参した。なお、患者は動悸や振戦がひどくて辛いと話している。検査値等は以下のとおりである。

（検査値）

脈拍数115拍/分、遊離サイロキシン（FT_4） 4 ng/dL、遊離トリヨードサイロニン（FT_3）10 pg/mL、甲状腺刺激ホルモン（TSH）0.05 μU/mL以下、TSH受容体抗体陽性

（処方1）

チアマゾール錠5 mg	1回3錠（1日3錠）
	1日1回　朝食後　14日分

（処方2）

プロプラノロール塩酸塩錠10 mg	1回1錠（1日3錠）
	1日3回　朝昼夕食後　14日分

問 258（薬理）

処方１及び処方２のいずれかの薬物の作用に関する記述のうち、正しいのはどれか。２つ選べ。

1　分解酵素活性化によるトリヨードサイロニンの分解促進

2　合成酵素阻害によるサイロキシンの合成阻害

3　TSH 受容体遮断による振戦の改善

4　アドレナリン β_1 受容体遮断による動悸の改善

5　アドレナリン β_2 受容体遮断による甲状腺ホルモンの遊離抑制

問 259（実務）

この患者への薬剤師の対応として、適切なのはどれか。２つ選べ。

1　妊娠の有無を再確認する。

2　服用開始後２ヶ月間は原則として２週に１回、白血球や好中球の検査が必要と伝える。

3　手指振戦や動悸の軽減には通常数週間かかると伝える。

4　処方薬服用後に発熱しても、それはバセドウ病の症状であり、薬の副作用ではないと伝える。

5　手指振戦等の自覚症状がなくなったら、両処方の服用は終了すると伝える。

問 260-261　48 歳男性。ぜん息の治療でシムビコートタービュヘイラー（ブデソニド・ホルモテロールフマル酸塩水和物配合）を使用している。人間ドックの眼圧検査により、高眼圧を指摘されたため、眼科を受診した。視力は右眼 0.4、左眼 0.5、眼圧は右 29 mmHg、左 25 mmHg、視神経乳頭陥凹が認められ、原発開放隅角緑内障と診断された。処方 1 で薬物療法を行い、1 ヶ月後の検査で眼圧は両眼ともに 22 mmHg に低下したが、効果不十分として処方 2 が追加された。

（処方 1 ）

ラタノプロスト点眼液 0.005％ 5 mL　1 本

1 回 1 滴　1 日 1 回　夕　両目点眼

（処方 2 ）

ブリンゾラミド懸濁性点眼液 1 ％ 5 mL　1 本

1 回 1 滴　1 日 2 回　朝夕　両目点眼

問 260（実務）

服薬指導時の薬剤師の説明内容として、適切なのはどれか。<u>２つ</u>選べ。

1　結膜嚢内に点眼する。

2　点眼後は瞬きしてよくなじませる。

3　夕に点眼する場合は処方 1 の薬剤から点眼し、1 分後に処方 2 の薬剤を点眼する。

4　点眼後は一時的に目がかすむことがあるので、症状が回復するまで自動車の運転等はしない。

5　十分効果が得られない場合は、1 回 2 滴まで点眼可能である。

問 261（薬理）

処方 2 の追加でも効果不十分であったため、処方 1 及び処方 2 とは作用機序が異なる薬物を処方 3 として追加することとなった。追加する処方 3 の薬物の作用機序として、最も適切なのはどれか。**1 つ**選べ。

1　アドレナリン β_2 受容体を遮断して、房水の産生を抑制する。
2　プロスタノイド FP 受容体を刺激して、ぶどう膜強膜からの房水流出を促進する。
3　炭酸脱水酵素を阻害して、房水の産生を抑制する。
4　Rho キナーゼ（ROCK）を阻害して、シュレム管からの房水流出を促進する。
5　アドレナリン α_2 受容体を遮断して、ぶどう膜強膜からの房水流出を促進する。

問 262-263　48 歳女性。月経あり。乳がん（ER 及び PgR 陽性、HER2 陰性）と診断され左乳房部分切除及び腋窩リンパ節郭清術を受けた。術後化学療法として、シクロホスファミド 600 mg/m^2、エピルビシン 100 mg/m^2 を 3 週毎に 4 サイクルを終了し、パクリタキセル 80 mg/m^2 を 3 週投与、 1 週休薬の 4 サイクルを開始している。卵巣機能は回復しており、術後化学療法終了後にタモキシフェンによる治療を検討中であるが、本患者においてはタモキシフェンと他剤との併用療法も選択可能である。担当薬剤師は 3 次資料を用いて、タモキシフェン単独療法と他剤との併用療法の有用性を調査することにした。

問 262（実務）

この場合用いる資料として優先順位が高いのはどれか。<u>２つ</u>選べ。ただし、これらの資料は調査時の最新版を用いることとする。

1　Drug Interaction: Analysis and Management

2　診療ガイドライン

3　UpToDate

4　Drugs in Pregnancy and Lactation: A Reference Guide to Fetal and Neonatal Risk

5　重篤副作用疾患別対応マニュアル

問 263（薬理）

調べた結果、術後化学療法後に卵巣機能が回復している場合、タモキシフェンに薬物Aを併用することが推奨されていた。なお、薬物Aは、この患者で術後化学療法として用いられた薬物とは作用機序が異なるものであった。薬物Aの作用機序として、最も適切なのはどれか。１つ選べ。

1　微小管タンパク質の脱重合を阻害して、細胞分裂を抑制する。

2　DNA の塩基対にインターカレーションして、DNA 依存性 RNA ポリメラーゼを阻害する。

3　エストロゲン受容体に結合して、内因性のエストロゲンと競合し抗腫瘍作用を発揮する。

4　DNA をアルキル化して、DNA 合成を阻害する。

5　持続的な性腺刺激ホルモン放出ホルモン（GnRH）受容体の刺激により脱感作を引き起して、ゴナドトロピンの遊離を抑制する。

問 264-265　薬剤師が特別養護老人ホームを訪問した時、施設の看護師から入所者が内服薬を服用しないので困っているとの相談を受けた。処方は朝食後にドネペジル塩酸塩錠 10 mg を 1 錠であった。現状を踏まえ、主治医に対し次回からリバスチグミン経皮吸収型製剤への変更を提案した。

問 264（実務）

薬剤変更を提案するにあたって、薬剤師が主治医に確認することとして、適切なのはどれか。２つ選べ。

1　患者が錠剤を飲まない時に貼付し、両剤を併用すること
2　患者が軽・中程度のアルツハイマー型認知症であること
3　患者が過去に貼付剤によってかぶれたことがあるか
4　薬剤変更後、毎週の増量が必要なこと
5　患者に腎機能障害がないこと

問 265（薬剤）

リバスチグミン経皮吸収型製剤の特徴として、誤っているのはどれか。**1つ**選べ。

1　背部又は胸部に貼付したとき、リバスチグミンの吸収には貼付部位間で差が認められない。

2　繰り返し貼付することで血漿中濃度は定常状態に達する。

3　肝初回通過効果を受けない。

4　主たる吸収経路は、皮膚における汗腺や毛穴などの付属器官である。

5　急激な血漿中濃度の上昇が回避される。

問 266-267　17歳男性。病的骨折を起こして精査の中で左脛骨骨肉腫と診断された。左膝関節離断術の後、翌月からメトトレキサート12 g/m^2/日、ドキソルビシン30 mg/m^2/日、シスプラチン120 mg/m^2/日による術後化学療法が開始された（全投与期間16週間、9コースから成るMAP法）。入院時の検査値、持参した一般用医薬品は以下のとおりであった。

（入院時の検査値）

白血球数5,300/μL、好中球数3,000/μL、Hb 12.1 g/dL、血小板数251 × 10^3/μL、AST 21 IU/L、ALT 22 IU/L、血清クレアチニン値0.82 mg/dL、eGFR 107 mL/min/1.73 m^2

（入院時に持参した一般用医薬品）

ファモチジン錠、ロキソプロフェン錠、ポビドンヨードうがい薬、酸化マグネシウム錠

問 266（薬剤）

この患者において、術後化学療法の施行中も、持参した一般用医薬品の服用を継続した場合、発現する可能性が最も高い薬物間相互作用はどれか。**1つ**選べ。

1　ドキソルビシンが、UGT1A1 を介したメトトレキサートのポリグルタミン酸化を阻害する。

2　ファモチジンが、ジヒドロ葉酸還元酵素を介したメトトレキサートの代謝を阻害する。

3　シスプラチンが、尿細管における有機カチオントランスポーター OCT2 を介したメトトレキサートの再吸収を阻害する。

4　酸化マグネシウムが、P-糖タンパク質を介したメトトレキサートの腸肝循環を阻害する。

5　ロキソプロフェンが、尿細管における有機アニオントランスポーター OAT3 を介したメトトレキサートの分泌を阻害する。

問 267（実務）

この患者に対して、第1週目（1コース目）のメトトレキサートを6時間単独静脈内投与することになった。医療チーム内で薬剤師が確認する事項として、適切でないのはどれか。**1つ**選べ。

1　メトトレキサート初回投与翌日より葉酸錠の内服を開始すること

2　メトトレキサート初回投与終了後よりホリナートカルシウム注を静注すること

3　メトトレキサート投与前日よりアセタゾラミド錠を内服していること

4　メトトレキサート投与翌日より24時間おきに3日間治療薬物モニタリング（TDM）を実施すること

5　メトトレキサート投与前日より持参したロキソプロフェン錠を使用中止すること

問 268-269　48歳男性。気管支ぜん息の既往があり、処方１及び処方２の薬剤を継続して使用している。この患者はテオフィリンの治療薬物モニタリング（TDM）を実施しており、定常状態の血中濃度は15 μg/mLであった。しかしここ数日、腹痛や吐き気が強く、今日は仕事も休んでいるとかかりつけ薬剤師に相談があった。聴き取りにより２日前からピロリ菌の除菌療法（処方３）をしていることが判明した。速やかにかかりつけ医を受診するように指示し、当該医師にも連絡を取った。その後、この患者について、受診時のテオフィリンの血中濃度が40 μg/mLであることを医師に確認した。なお、アドヒアランスは良好であることを確認している。

（処方１）

ブデソニド・ホルモテロールフマル酸塩水和物吸入液　１回２吸入
１日２回　朝食後・就寝前　７日分

（処方２）

テオフィリン徐放錠 200 mg	１回１錠（１日２錠）
	１日２回　朝食後・就寝前　７日分

（処方３）

ボノプラザン錠 20 mg	１回１錠（１日２錠）
アモキシシリンカプセル 250 mg	１回３カプセル（１日６カプセル）
クラリスロマイシン錠 200 mg	１回２錠（１日４錠）
	１日２回　朝夕食後　７日分

問 268（実務）

薬剤師がこの患者のテオフィリン中毒の要因と考えた内容として、最も適切なのはどれか。**1つ**選べ。

1 ピロリ菌の除菌療法による胃内環境の変化
2 ボノプラザンによる胃内 pH の上昇
3 腎薬物トランスポーターを介したアモキシシリンとの競合阻害
4 クラリスロマイシンによる肝薬物代謝酵素阻害
5 ぜん息症状によるテオフィリン感受性の増大

問 269（薬剤）

この患者が処方 2 の薬剤の服用を中止し、テオフィリンの血中濃度が 15 μg/mL に低下するまでに要する時間として最も近いのはどれか。**1つ**選べ。

ただし、テオフィリンの血中動態は線形 1-コンパートメントモデルに従うものとし、血中消失半減期は 6.9 時間とする。なお、ln2 = 0.69、ln3 = 1.10 とする。

1 8 時間
2 10 時間
3 12 時間
4 14 時間
5 16 時間

問 270-271　27 歳男性。体重 50 kg。父をドナーとする生体腎移植治療が予定されている。7 日後の移植術を控え、術後に用いるタクロリムスの投与設計を薬剤師が依頼された。

問 270（薬剤）

この患者にタクロリムスを経口投与し、24 時間採血を行った際の血中濃度時間曲線下面積（$AUC_{0-\infty}$）は 120 μg・h/L、一次モーメント曲線下面積（$AUMC_{0-\infty}$）は 1,320 μg・h^2/L であった。また、タクロリムス 0.5 mg を急速静注した直後の血中濃度は 10 ng/mL であった。この患者にタクロリムスを 1 日 1 回経口投与し、定常状態における平均血中濃度を 10 ng/mL としたい。適切な投与量（mg）に最も近い値はどれか。**1 つ**選べ。

ただし、タクロリムスの吸収速度定数を 1.0 h^{-1} とし、バイオアベイラビリティを 0.2 とする。また、タクロリムスの体内動態は線形 1-コンパートメントモデルに従うものとし、反復投与によってタクロリムスの体内動態は変化しないものとする。

1　1.0
2　1.2
3　3.0
4　5.5
5　6.0

問 271（実務）

術前の投与設計によって、タクロリムスカプセルの投与を手術当日夕食後より開始した。7日後に退院予定であるが、病棟担当薬剤師が行う患者への指導内容として、適切なのはどれか。２つ選べ。

1　クロレラの摂取を控える。

2　加熱調理した野菜の摂取を控える。

3　グレープフルーツの摂取を控える。

4　乾燥弱毒生風しんワクチンの接種を控える。

5　インフルエンザ HA ワクチンの接種を控える。

問 272-275　65 歳男性。身長 160 cm、体重 58 kg。開胸心血管バイパス術施行後 4 日目に 38.5 ℃の発熱を来し、喀痰、血液培養、尿、鼻汁を用いたグラム染色の結果、陽性であった。細菌培養の結果が得られるまで 48 時間程度を要することから、院内感染制御チームへのコンサルトの結果、MRSA 感染症を疑い、当日夜よりバンコマイシン点滴静注用の 14 日間投与が決定された。バンコマイシン投与前の検査値を以下に示す。

（検査値）

白血球数 13,000/μL、CRP 7.5 mg/dL、血清クレアチニン値 1.2 mg/dL、BUN 17.6 mg/dL、クレアチニンクリアランス（Ccr）50 mL/min

バンコマイシンの投与量決定には母集団薬物動態解析により得られた以下のパラメータを用いた。

CL(L/hr) = 0.05 × Ccr(mL/min)［Ccr が 85 mL/min 以下の場合］

CL(L/hr) = 3.5［Ccr が 85 mL/min より大きい場合］

Vd(L) = 60.7

問 272（薬剤）

母集団薬物動態解析及びこの患者の投与量決定に関する記述のうち、正しいのはどれか。<u>**2つ**</u>選べ。

1　患者集団の平均的な薬物動態パラメータは、年齢、体重、Vd が同じ患者群を母集団として解析することで得られる。

2　バンコマイシンのクリアランスは最小発育阻止濃度（MIC）により影響をうける。

3　この患者の投与量決定には分布容積 60.7 L とクリアランス 2.5 L/h を用いた。

4　患者の 1 点の血中濃度測定値、患者情報、及び母集団パラメータとその変動要因を用いて、ベイジアン法により患者個々の薬物動態パラメータが推定できる。

5　母集団パラメータを求めるためには、集団ごとに血液採取時間を一定にする必要がある。

問 273（薬理）

バンコマイシンの治療効果及び副作用に関する記述のうち、正しいのはどれか。**２つ**選べ。

1　ペンタペプチドC末端のD-Ala-D-Alaに結合して、細胞壁合成を抑制する。
2　細菌リボソーム30Sサブユニットに結合して、タンパク質合成を抑制する。
3　翻訳過程の50S開始複合体の形成を阻害して、タンパク質合成を抑制する。
4　ヒスタミンの遊離を促進して、レッドネック症候群を引き起こす。
5　非ステロイド性抗炎症薬との併用により、痙れんを引き起こす。

問 274（実務）

バンコマイシン投与後の副作用確認のために薬剤師が行うモニタリングとして、適切なのはどれか。**２つ**選べ。

1　投与直後はアナフィラキシーショックが発現することがあるので、皮疹や呼吸困難の有無を確認する。
2　高血圧が発現しやすいので、朝晩の血圧を確認する。
3　第8脳神経障害の副作用が発現することがあるので、視力を確認する。
4　低アルブミン血症の発現頻度が高いので、面談時に全身のむくみを確認する。
5　腎障害が発現することがあるので、血清クレアチニン値や尿量を確認する。

問 275（実務）

バンコマイシン投与開始後、以下の経過をたどった。

1日目　（バイパス術施行後4日目）バンコマイシン点滴静注用の投与開始。
3日目　血液サンプルの細菌培養でMRSA陽性。そのMICは2.0μg/mL。
7日目　体温36.5℃。
10日目　CRP 0.2 mg/dL。体温36.2℃。白血球数2,500/μL。

以上の治療経過を踏まえた病棟担当薬剤師の主治医への対応について、最も適切なのはどれか。**１つ**選べ。

1　バンコマイシン散への変更を提案した。
2　バンコマイシンの目標血中濃度は15μg/mLにするべきであると提案した。
3　既に解熱しているので、バンコマイシンを全期間投与せず、早期終了を提案した。
4　再度、喀痰、血液、尿、鼻汁の細菌検査を依頼するよう提案した。
5　バンコマイシンによる血球減少を疑い、投与中止を提案した。

問 276-277 55 歳男性。2 型糖尿病。内服薬による血糖のコントロールが不良のため、インスリン導入の目的で教育入院を行い、超速効型インスリンの投与が開始された。しかし退院後、仕事が多忙のため自己注射が不規則になった。現状の改善が図れないことから、かかりつけ薬剤師が処方医にトレーシングレポートを書き、使用製剤の見直しについて処方提案を行った。その結果、次回来局時には以下のように変更された処方箋を持参した。

ライゾデグ配合注フレックスタッチ (注)

1 回 12 単位　1 日 1 回　朝食直前

（注）インスリン デグルデク（遺伝子組換え）・インスリン アスパルト（遺伝子組換え）溶解インスリンアナログ注射液

問 276（実務）

生活の状況を考慮して処方変更となった患者への説明として、適切なのはどれか。**1 つ**選べ。

1　以前処方されていた超速効型インスリンも併用する。
2　注射をする前に、十分に転倒混和して懸濁させる。
3　注射を忘れた日は、空腹時でも注射する。
4　風邪に伴う発熱や悪寒が現われても自己判断で中止しない。
5　血糖値に応じて適宜注入単位を調整する。

問 277（薬剤）

図は、皮下投与後のインスリンアナログの動態を示している。インスリンアナログの動態に関する記述のうち、正しいのはどれか。2つ選べ。

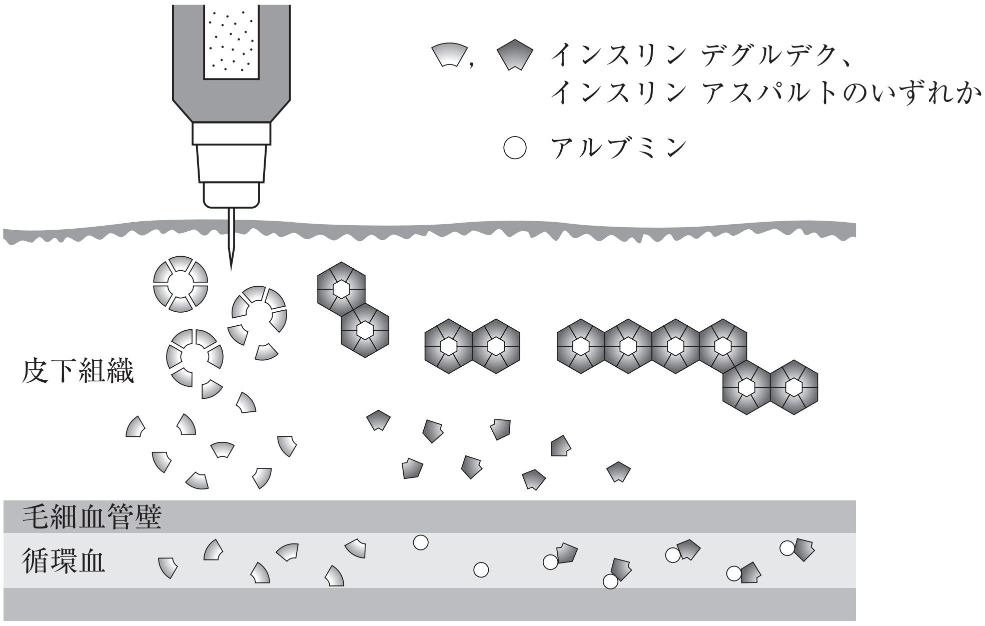

1 本製剤中で、インスリン デグルデクは、難水溶性で安定なダイヘキサマーとして存在する。

2 本製剤中で、インスリン アスパルト及びインスリン デグルデクは、アルブミンと結合し安定化されている。

3 本製剤を皮下投与後、インスリン アスパルトのヘキサマーは、皮下組織において速やかにモノマーに解離する。

4 本製剤を皮下投与後、インスリン デグルデクのダイヘキサマーは、皮下組織でマルチヘキサマーを形成した後、徐々にモノマーに解離する。

5 インスリン アスパルトは、循環血中でアルブミンに強く結合し、標的組織に移行する。

問 278-279　72 歳男性。経口血糖降下薬を用いた治療を受けていたが、健康診断にて腎機能は正常であるが肝機能の異常を指摘され、精査目的で入院となった。病棟担当薬剤師が入院時持参薬の鑑別結果をもとに初回面談の際に指導を行う予定である。

（持参薬）

グリメピリド錠 1 mg	1 回 1 錠（1 日 1 錠）
	1 日 1 回　朝食後　30 日分
ビルダグリプチン錠 50 mg	1 回 1 錠（1 日 2 錠）
メトホルミン塩酸塩錠 250 mg	1 回 2 錠（1 日 4 錠）
	1 日 2 回　朝夕食後　30 日分

問 278（実務）

主治医からの情報で、本患者には肝腫瘍の疑いがあるため、明後日朝にイオパミドール注射液を用いた画像検査が予約されていることが判明した。病棟担当薬剤師が患者に対して行う説明の内容として、適切なのはどれか。2つ選べ。

1 本画像検査に際しメトホルミンの服用は検査前日から休止する。
2 グリセリン浣腸を本画像検査前夜に行う。
3 本剤の主成分はX線吸収能が高いため、腫瘍を明瞭に描出できる。
4 造影剤を使用しても腎機能については特に注意する必要はない。
5 十分な水分補給は画像検査後の強い痒みが出る場合に行う。

問 279（薬剤）

イオパミドール注射液には以下の3種類のバイアル製剤がある。これら注射剤の粘度に関する記述のうち、正しいのはどれか。2つ選べ。

ただし、37℃における水の粘度0.70 mPa・sとする。

製剤	A	B	C
区分	注射液（水溶液）		
日局イオパミドール含有量（mg/mL）	306.2	612.4	755.2
粘度（mPa・s、37℃）	1.5	4.4	9.1
密度（kg/m^3、37℃）	1,171	1,328	1,405

1 製剤Aの動粘度は、製剤Bの動粘度より小さい。
2 製剤Bの相対粘度は、製剤Cの相対粘度より小さい。
3 製剤Cの比粘度は、製剤Bの比粘度より小さい。
4 いずれの製剤も、イオン性造影剤に比べて高粘度のため、組織障害性が低減されている。
5 製剤Cの還元粘度は、製剤Bの還元粘度より小さい。

問 280-281　32 歳女性。全大腸型潰瘍性大腸炎と診断され、プレドニゾロンで加療していたが再燃を繰り返したため、プレドニゾロンをインフリキシマブのバイオシミラー製剤に変更したところ軽快した。インフリキシマブに変更して 6 ヶ月目に全身倦怠感と顔面（頬部）の広範な紅斑を認め、TNF 阻害薬誘発性のループス様症状と診断された。

問 280（薬剤）

インフリキシマブのバイオシミラーに関する記述のうち、正しいのはどれか。**1つ**選べ。

1　先行バイオ医薬品の欠点を改良した完全ヒト化抗体である。

2　先行バイオ医薬品と同一の糖鎖を有する。

3　先行バイオ医薬品と同等／同質の安全性、有効性を有する。

4　臨床試験において、生物学的同等性試験による評価が必要である。

5　先行バイオ医薬品と同一の細胞を用いて製造される。

問 281（実務）

ループス様症状は、インフリキシマブ投与の中止と高用量のプレドニゾロン投与により軽快した。このとき、消化性潰瘍の予防として使用されたのはどれか。**1つ**選べ。

1　メサラジン

2　アレンドロン酸

3　フェブキソスタット

4　エトドラク

5　ランソプラゾール

問 282-283　54 歳女性。体重 60 kg。腋窩リンパ節転移が著明な進行性乳がんと診断され、トラスツズマブを含む化学療法を継続していた。最近の画像検査にて肝転移を認めたため、以下の化学療法を施行することとなった。

薬品名	投与経路	投与時間
①生理食塩液 50 mL	点滴静注	5 分
②トラスツズマブ エムタンシン(注) 3.6 mg/kg ＋ 生理食塩液 250 mL	点滴静注	90 分
③生理食塩液 50 mL	点滴静注	5 分

1 クールの日数：21 日

（注）トラスツズマブに抗がん薬 DM1 が結合した構造を有する薬剤で、白色の塊である。

問 282（実務）

本化学療法レジメンの運用に際して院内で合意された内容のうち、適切なのはどれか。**1つ**選べ。

1　①の生理食塩液は省略できる。
2　②のトラスツズマブ エムタンシンの投与時間は初回に限り30分に短縮できる。
3　②のトラスツズマブ エムタンシンは5％ブドウ糖液で溶解する。
4　③の生理食塩液は省略できる。
5　②のトラスツズマブ エムタンシン溶解液はインラインフィルターを用いて投与する。

問 283（薬剤）

トラスツズマブ エムタンシン製剤に関する記述のうち、正しいのはどれか。**2つ**選べ。

1　有効成分は、分子標的薬である核酸医薬と抗がん薬DM1が結合した核酸薬物複合体である。
2　有効成分は、ヒト上皮増殖因子と抗がん薬DM1が結合したタンパク質薬物複合体である。
3　有効成分は、点滴静注後、ヒト上皮増殖因子受容体2型（HER2）を標的として抗がん薬DM1を能動的にターゲティングする。
4　有効成分が細胞内に取り込まれた後、リソソーム内で抗がん薬DM1を遊離する。
5　有効成分が細胞膜上の受容体に結合した後、遊離した抗がん薬DM1が細胞膜に傷害を引き起こす。

問 284-285　近隣で喫茶店を営む高齢男性から、体調変化に関してかかりつけ薬剤師に相談があった。以前から神経因性疼痛があり、一般用医薬品の芍薬甘草湯を服用しているが、「最近、足がむずむずして気持ち悪いことが多く、夜も寝られないことがある。」とのことだった。近医を紹介し、お薬手帳を持参の上で受診するように提案した。その後、紹介した医師より、ドパミンアゴニスト使用不可のレストレスレッグス症候群（下肢静止不能症候群）と診断され、以下の処方を考えている旨の連絡がこのかかりつけ薬剤師にあった。

ガバペンチン エナカルビル錠（注）300 mg　1回2錠（1日2錠）
1日1回　夕食後　14日分

（注）徐放錠である。

問 284（実務）

かかりつけ薬剤師が、患者や医師に対して行う処方薬に関する説明として適切なのはどれか。２つ選べ。

1 飲みにくい場合は、自身で飲みやすいサイズに分割するように患者に説明する。
2 用量調整が必要になるので、肝機能検査値を医師に確認する。
3 芍薬甘草湯は併用できないので、服用中止を患者に説明する。
4 霧視等の眼障害について、診察時に医師から説明を受けたかについて患者に確認する。
5 眠気等が起こることがあるので自動車の運転は控えることを患者に説明する。

問 285（薬剤）

ガバペンチン エナカルビル及びその製剤に関する記述のうち、正しいのはどれか。２つ選べ。

1 ガバペンチン エナカルビルは、ガバペンチンの経口吸収のばらつきや飽和を改善するプロドラッグである。
2 ガバペンチン エナカルビルは、カルボキシルエステラーゼによる代謝を受けて活性代謝物ガバペンチンに変換される。
3 ガバペンチン エナカルビルは、ガバペンチンと同じ Ca^{2+} チャネルを介して消化管から吸収される。
4 本錠剤には、崩壊剤としてグリセリン脂肪酸エステルが含まれる。
5 本錠剤は、腸溶性を示す。

107

2日目③　　一般問題（薬学実践問題）

【病態・薬物治療、法規・制度・倫理／実務、実務】

◎指示があるまで開いてはいけません。

注　意　事　項

1　試験問題の数は、**問２８６から問３４５までの６０問**。
　１５時３０分から１８時までの１５０分以内で解答すること。

2　解答方法は次のとおりである。

(1)　一般問題（薬学実践問題）の各問題の正答数は、**問題文中に指示されている**。
　問題の選択肢の中から答えを選び、次の例にならって答案用紙に記入すること。
　なお、問題文中に指示された正答数と**異なる数を解答すると、誤りになる**から注意すること。

（例）**問 500**　次の物質中、常温かつ常圧下で液体のものはどれか。**２つ**選べ。

1　塩化ナトリウム　　2　プロパン　　3　ベンゼン
4　エタノール　　5　炭酸カルシウム

正しい答えは「3」と「4」であるから、答案用紙の

問 500 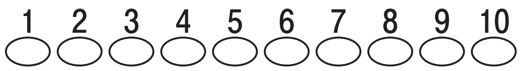 のうち 3 と 4 を塗りつぶして

問 500 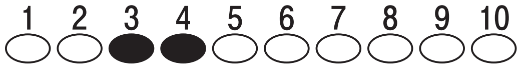 とすればよい。

(2)　解答は、〇の中全体をＨＢの鉛筆で濃く塗りつぶすこと。塗りつぶしが薄い場合は、解答したことにならないから注意すること。

悪い解答例 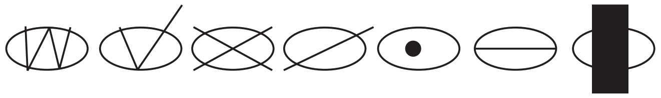 **（採点されない）**

(3)　解答を修正する場合は、必ず「消しゴム」で跡が残らないように完全に消すこと。鉛筆の跡が残ったり、「 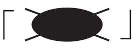 」のような消し方などをした場合は、修正又は解答したことにならないから注意すること。

(4)　答案用紙は、折り曲げたり汚したりしないよう、特に注意すること。

3　設問中の科学用語そのものやその外国語表示（化合物名、人名、学名など）には誤りはないものとして解答すること。ただし、設問が科学用語そのもの又は外国語の意味の正誤の判断を求めている場合を除く。

4　問題の内容については質問しないこと。

一般問題（薬学実践問題）【病態・薬物治療、法規・制度・倫理／実務】

問 286-287　68 歳女性。54 歳の頃、精神科でうつ病と診断され 2 年間ほどセルトラリン塩酸塩錠を服用し、回復した。10 年前（58 歳時）に内科でパーキンソン病と診断され、レボドパ 250 mg・カルビドパ配合錠（1 日 5 錠、朝 2 錠、昼 1 錠、夕 2 錠）で治療を開始した。3 年前（65 歳時）から薬の作用時間が短縮し、服用後時間が経つと安静時振戦や運動緩慢など症状の悪化が見られた。舌突出・異常運動、じっとしていられないなどの症状は出現していなかった。服用回数を 5 回に分割したところ症状は落ち着いた。

問 286（病態・薬物治療）

服用回数を分割する前に、患者に出現していた症状はどれか。**1 つ**選べ。

1　アカシジア
2　急性ジストニア
3　遅発性ジスキネジア
4　on-off 現象
5　wearing-off 現象

問 287（実務）

この患者は、その後、薬を頻回に内服することを考えると気分がすぐれなくなり、うつ病が再発したため、2年前（66歳時）から精神科でセルトラリン塩酸塩錠の服用を再開した。2ヶ月ほど前から、3年前（65歳時）のような症状が起こるようになったと、内科の主治医に相談があった。主治医は、新しく薬物を追加することを検討している。現在の処方は以下のとおりである。

（処方1）

レボドパ 250 mg・カルビドパ配合錠　1回1錠（1日5錠）

1日5回　起床時、10時、14時、18時、22時　28日分

（処方2）

セルトラリン塩酸塩錠 50 mg　1回1錠（1日1錠）

1日1回　朝食後　28日分

この患者に追加する薬物として、適切でないのはどれか。**1つ**選べ。

1　セレギリン
2　ロピニロール
3　イストラデフィリン
4　エンタカポン
5　ゾニサミド

問 288-289　28 歳女性。病院で受付事務の仕事をしている。半年前から起床時の体のこわばりや、手足の関節の痛みを意識するようになった。市販の鎮痛薬を飲んでいたが、徐々に増悪したため、近くの整形外科を受診したところ、関節リウマチと診断された。メトトレキサートでの治療を開始したが、症状は改善しなかった。アダリムマブの自己注射を追加することになり、自己注射について薬剤師の指導を受けるように医師から言われ来局した。

患者の検査値等は以下のとおりである。

赤血球数 280 × 10^4/μL、白血球数 8,000/μL、血小板数 12 × 10^4/μL、
CRP 2.8 mg/dL、血清クレアチニン値 0.7 mg/dL、HbA1c 5.2%（NGSP 値）、
胸部 X 線　異常なし。

問 288（実務）

薬剤師が患者に行う指導として、適切なのはどれか。2つ選べ。

1　注射後、発疹や呼吸困難が現れた場合は、すぐに医師の診察を受ける。
2　注射部位は、大腿部、腹部又は上腕部を選び、毎回、同一の場所に打つ。
3　微熱や咳が続くときには、すぐに医師の診察を受ける。
4　風しんの罹患歴がない場合は、風しんワクチンをすみやかに接種するよう指導する。
5　メトトレキサートは毎日、決まった時刻に服用を続ける。

問 289（病態・薬物治療）

アダリムマブの自己注射を開始後、関節リウマチの症状は軽快して、患者は大変喜んでいたが、約 6 ヶ月後、次第に湿性の咳と全身倦怠感が出現するようになった。

（検査値及び所見）

赤血球沈降速度 30 mm/h（基準値 3 ～15）、CRP 1.0 mg/dL、

HbA1c 5.4%（NGSP 値）、インターフェロンガンマ遊離試験 陽性

この状況に関する記述として、正しいのはどれか。2つ選べ。

1 関節リウマチの再燃が疑われる。

2 糖尿病発症の可能性がある。

3 結核感染が疑われる。

4 アバタセプトの併用を検討する必要がある。

5 アダリムマブの投与中止を検討する必要がある。

問 290-291　58 歳男性。身長 162 cm、体重 88 kg。10 年前から健康診断で、高血圧及び高血糖を指摘されていたが放置していた。喫煙歴 30 年（1 日 10 本程度)、20 歳ごろよりビール大びん 2 本と日本酒 1 合程度をほぼ毎日飲酒していた。数ヶ月前より、全身倦怠感が次第に強くなってきているのを自覚していたが、本日、外出中に駅の階段で動けなくなり、救急搬送された。

(来院時の所見及び検査値)

意識は清明であり、四肢に運動・感覚障害は認めない。
血圧 190/110 mmHg、心拍数 72 拍/分、AST 210 IU/L、ALT 150 IU/L、
γ-GTP 175 IU/L、血清クレアチニン値 0.71 mg/dL、
血清浸透圧 300 mOsm/L、血糖値 310 mg/dL、HbA1c 10.5％(NGSP 値)、
尿糖（3＋)、尿中アルブミン正常、尿蛋白（－)、尿中ケトン体（3＋)、
浮腫（－）

問 290（病態・薬物治療）

この患者に起きている状況として、考えられるのはどれか。**２つ**選べ。

1　高血圧緊急症
2　くも膜下出血
3　糖尿病性腎症
4　高浸透圧高血糖症候群
5　糖尿病性ケトアシドーシス

問 291（実務）

上記患者は、１ヶ月の入院加療後退院し、以下の処方で通院治療を続け、３年が経過した。

（処方１）

メトホルミン塩酸塩錠 500 mg	1 回 1 錠（1 日 3 錠）	
	1 日 3 回　朝昼夕食後	28 日分

（処方２）

シタグリプチンリン酸塩水和物錠 50 mg	1 回 1 錠（1 日 1 錠）	
	1 日 1 回　朝食後	28 日分

（処方３）

アムロジピン錠 10 mg	1 回 1 錠（1 日 1 錠）	
	1 日 1 回　朝食後	28 日分

運動療法及び食事療法も指導されたとおり実践しており、処方された薬剤は指示どおり服薬していたが、飲酒はやめられないと話している。今回の検査で以下の結果となり、再教育のため入院となった。

（所見及び検査値）

入院時体重 68 kg、血圧 140/85 mmHg、心拍数 70 拍/分、AST 35 IU/L、ALT 42 IU/L、γ-GTP 162 IU/L、血清クレアチニン値 2.6 mg/dL、空腹時血糖値 180 mg/dL、HbA1c 8.2%（NGSP 値）、尿糖（＋）、尿蛋白（2＋）、尿中ケトン体（－）、下肢浮腫（－）

この患者に対する処方提案のうち、適切なのはどれか。２つ選べ。

1　メトホルミンの増量
2　シタグリプチンをリナグリプチンに変更
3　フロセミドの追加
4　テルミサルタンの追加
5　アムロジピンの増量

問 292-293　43 歳男性。基礎疾患はない。海外に単身赴任中。一時帰国した 3 日後の夕食時に体調がすぐれず、早めに就寝した。翌朝から 39 ℃の発熱と発疹を認め、近医を受診し、風しんと診断された。

家族の風しん罹患歴、予防接種歴は以下のとおりである。

家族	年齢	身体状況	風しんに関する情報
母	76 歳	健康	子供の頃に罹患歴あり
妻	39 歳	妊娠中	罹患歴なし、抗体価不明
子	6 歳	健康	予防接種 2 回済

問 292（病態・薬物治療）

この患者に関する記述として、考えられるのどれか。2つ選べ。

1　帰国途中又は帰国後に感染した。

2　DNA ウイルスに感染した。

3　リンパ節の膨張が認められる。

4　白血球数が減少している。

5　発熱は 2 週間以上続く。

問 293（実務）

この患者への対応として、適切なのはどれか。2つ選べ。

1　この患者に、療養中に他の医療機関を受診する際は、不安を与えないために風しん感染の情報を伝えないよう勧める。

2　この患者に、妻との濃厚接触を避けて療養するよう伝える。

3　この患者の母は、風しんウイルス抗体を有していると考えられると伝える。

4　この患者の妻は、今すぐ風しんワクチン接種を受ける必要があると伝える。

5　この患者の子は、今すぐ 3 回目の風しんワクチン接種を受ける必要があると伝える。

問 294-295　62 歳男性。身長 161 cm、体重 58 kg。半年ほど前から腋窩のしこりに気づいていたが、徐々に増大してきた。１ヶ月前よりだるさと 38 ℃の発熱が継続し、朝起きたときに下着が濡れているほどの汗をかくようになった。体重も減少してきたため、心配になって病院を受診した。患者の検査値等は以下のとおりである。

（検査値及び所見）

AST 51 IU/L、ALT 38 IU/L、LDH 2,543 IU/L、γ-GTP 224 IU/L、
血清クレアチニン値 1.62 mg/dL、尿酸 8.4 mg/dL、血清 Na 136 mEq/L、
血清 K 4.5 mEq/L、血清 Ca 10.0 mg/dL、血清 P 3.0 mmol/L、
血清アルブミン 4.0 g/dL、HbA1c 5.8%（NSGP 値）、白血球数 15,000/μL、
赤血球数 250 × 10^4/μL、Ht 35%、腋窩の腫瘍径は 5 cm

精査の結果、悪性リンパ腫と診断されたが、リンパ節生検でリード・ステルンベルグ（Reed-Sternberg）細胞などの巨細胞は確認されなかった。

問 294（病態・薬物治療）

この患者の病態及び検査に関する記述のうち、正しいのはどれか。<u>２つ</u>選べ。

1　非ホジキンリンパ腫の症例と考えられる。
2　発熱、大量の寝汗及び体重減少は、B 症状の典型的症状である。
3　血清カルシウム値が高いため、骨破壊が進んでいる可能性が高い。
4　遺伝子検査では、フィラデルフィア染色体が検出される可能性が高い。
5　腎機能低下は、ベンス・ジョーンズタンパク質の増加による可能性が高い。

問 295（実務）

この患者は入院して R-CHOP 療法を施行することになった。

R-CHOP 療法

薬物名・投与量	Day 1	Day 2-5
リツキシマブ 375 mg/m^2	○	
ドキソルビシン 50 mg/m^2	○	
ビンクリスチン 1.4 mg/m^2	○	
シクロホスファミド 750 mg/m^2	○	
プレドニゾロン 30 mg/body	○	○

治療を開始する前の薬剤師の対応として、適切でないのはどれか。**1つ**選べ。

1　ラスブリカーゼの投与を提案する。
2　血糖測定を提案する。
3　B 型肝炎ウイルスへの感染の有無を確認する。
4　ビンクリスチンの累積投与量が上限を超えないことを確認する。
5　心不全がないことを確認する。

問 296-297　54 歳男性。既往歴なし。咳と嗄声が継続していたが、血痰を認めたため近医を受診した。胸部 X 線で右肺腫瘤を指摘され、総合病院呼吸器内科を紹介受診した。精査の結果 cT2N3M1b Stage Ⅳ A の非小細胞肺がん（腺がん）と診断された。パフォーマンスステータス（PS）１。治療薬選択にあたり、遺伝子検査が実施された。*EGFR* 遺伝子変異（陰性）、*ALK* 遺伝子転座（陰性）、*ROS1* 遺伝子転座（陽性）、*BRAF* 遺伝子変異（陰性）、PDL-1 ≧ 50%。

患者に喫煙歴はなく、機会飲酒のみ。就学中の子供がいるため、外来通院治療を希望している。

問 296（実務）

この患者の一次治療薬として、適切なのはどれか。2つ選べ。

1　エルロチニブ
2　クリゾチニブ
3　ゲフィチニブ
4　ペムブロリズマブ
5　アレクチニブ

問 297（病態・薬物治療）

この患者の病態及び治療に関する記述のうち、適切なのはどれか。2つ選べ。

1　腫瘍マーカーの PSA が上昇している。
2　他臓器への遠隔転移がある。
3　子孫に遺伝する変異が検出された。
4　子供と一緒に散歩することができる。
5　手術による根治が可能である。

問 298-299　62 歳女性。身長 153 cm、体重 56 kg。動悸及び息切れを自覚し、近医を受診したところ非弁膜症性心房細動と診断され、以下の処方で治療を開始することになった。患者の検査値等は以下のとおりである。

（所見及び検査値）

血圧 140/86 mmHg、心拍数 160 拍/分、脈拍数 90 拍/分、AST 23 IU/L、ALT 28 IU/L、eGFR 40 mL/min/1.73 m^2

（心電図）

RR 間隔不規則、P 波消失、f 波出現

（処方１）

ワルファリンカリウム錠 1 mg	1 回 2 錠（1 日 2 錠）<br>1 日 1 回　朝食後　14 日分

（処方２）

ビソプロロールフマル酸塩錠 2.5 mg	1 回 1 錠（1 日 1 錠）<br>1 日 1 回　朝食後　14 日分

問 298（病態・薬物治療）

治療薬の処方意図として、適切なのはどれか。２つ選べ。

1　心拍数の調節（レートコントロール）
2　洞調律の維持（リズムコントロール）
3　冠動脈血栓の予防
4　肺塞栓症の予防
5　脳塞栓症の予防

問 299（実務）

３ヶ月経過後、患者が処方箋を持って来局した。処方が以下の内容に変更されていた。

（処方１（変更後））

ダビガトランエテキシラートメタンスルホン酸塩カプセル 110 mg

1 回 1 カプセル（1 日 2 カプセル）

1 日 2 回　朝夕食後　14 日分

（処方２）

ビソプロロールフマル酸塩錠 2.5 mg　　1 回 1 錠（1 日 1 錠）

1 日 1 回　朝食後　　14 日分

処方内容の変更について薬剤師が患者に確認したところ、以下の答えが返ってきた。「薬の量を決めるために検査を繰り返していたが、主治医から食事について質問され、時折青汁を飲んでいることを伝えたところ、薬を変えることになった。」

患者の所見及び検査結果は以下のとおりである。

（所見及び検査値）

血圧 130/85 mmHg、心拍数 120 拍/分、脈拍数 75 拍/分、AST 25 IU/L、ALT 26 IU/L、eGFR 35 mL/min/1.73 m^2、PT-INR 2.3

今回の処方変更について、薬剤師の対応として、正しいのはどれか。**２つ**選べ。

1　ワルファリンカリウム錠の服用を中止し、その翌日よりダビガトランエテキシラートメタンスルホン酸塩カプセルを開始するよう患者に説明する。

2　ダビガトランエテキシラートメタンスルホン酸塩カプセルを服用し忘れた場合、できるだけ早く 1 回量を服用し、次の服用まで 6 時間以上空けるよう指導する。

3　青汁やほうれん草などの緑黄色野菜の摂取は、控えるように患者に指導する。

4　他科や他院で P-糖タンパク質を阻害する薬剤が処方されていないことを確認する。

問 300-301　72 歳男性。S 状結腸穿孔により腹膜炎を発症し、敗血症性ショックの診断で集中治療室（ICU）に入室となった。人工呼吸器管理下でノルアドレナリン注射液、ドブタミン塩酸塩注射液及び注射用メロペネムを使用していたが、皮下出血、血小板数の低下、プロトロンビン時間の延長及びフィブリノゲンの低下が観察され、敗血症性播種性血管内凝固症候群（DIC）と診断された。DIC 診断に伴い、未分画ヘパリンおよびガベキサートの投与が開始された。開始後は活性化部分トロンボプラスチン時間（APTT）の延長が認められたが、数日後 APTT の延長が乏しくなった。現在の所見及び検査値は以下のとおりである。

（所見及び検査値）

体温 37.6 ℃、脈拍数 92 拍/分、呼吸数 22 回/分、赤血球数 500 × 10^4/μL、
白血球数 14,500/μL、血小板数 6.1 × 10^4/μL、CRP 7.8 mg/dL、
アンチトロンビン活性 62%、APTT 18.1 秒（基準対照 32.2)、
フィブリン・フィブリノゲン分解産物（FDP）20.4 μg/mL（基準値 < 5）

問 300（病態・薬物治療）

この患者に関する記述のうち、正しいのはどれか。<u>**２つ**</u>選べ。

1　D ダイマー/FDP の比は低下している。
2　ガベキサートの使用により、出血リスクが高くなっている。
3　細小血管に微小血栓が形成されている。
4　死に至る可能性は極めて低い。
5　腎機能のモニタリングが必要である。

問 301（実務）

今後の治療について、医師より ICU 担当薬剤師に意見を求められた。適切な提案はどれか。2つ選べ。

1　未分画ヘパリンを中止し、トラネキサム酸を投与する。

2　アンチトロンビンガンマを投与する。

3　出血がないことを確認して、トロンボモデュリンアルファを投与する。

4　人赤血球液を投与する。

5　アスピリンを投与する。

問 302-303　59 歳男性。B 型肝炎ウイルス（HBs 抗原）陽性であったが症状もなく長年放置していた。倦怠感や意識障害が強くなり家族に連れられ近医を受診したところ、非代償性肝硬変と診断され、緊急入院となった。下肢にむくみを認めているが、食事の摂取は可能である。入院時の検査値と入院後の処方は以下のとおりである。

（検査値）

AST 26 IU/L、ALT 27 IU/L、血清クレアチニン値 1.2 mg/dL、
総タンパク 6.0 g/dL、血清アルブミン 2.4 g/dL、LDL-C 38 mg/dL、
プロトロンビン時間（PT）19.8 秒、総ビリルビン 1.0 mg/dL、
直接ビリルビン 0.6 mg/dL

（処方 1）

ラミブジン錠 100 mg	1 回 1 錠（1 日 1 錠）
フロセミド錠 20 mg	1 回 1 錠（1 日 1 錠）
スピロノラクトン錠 25 mg	1 回 1 錠（1 日 1 錠）
	1 日 1 回　朝食後　3 日分

（処方 2）

ウルソデオキシコール酸錠 50 mg	1 回 1 錠（1 日 3 錠）
	1 日 3 回　朝昼夕食後　3 日分

（処方 3）

酸化マグネシウム錠 330 mg	1 回 2 錠（1 日 6 錠）
ラクツロースゼリー分包 16.05 g/包	1 回 1 包（1 日 3 包）
分岐鎖アミノ酸配合経口ゼリー剤 20 g/個	1 回 1 個（1 日 3 個）
	1 日 3 回　朝昼夕食後　3 日分

問 302（病態・薬物治療）

入院時、この患者に起こっていることとして、適切なのはどれか。２つ選べ。

1　血清アルブミン濃度の低下

2　血清コレステロール濃度の上昇

3　フィッシャー比の上昇

4　プロトロンビン時間の延長

5　直接ビリルビン濃度の低下

問 303（実務）

この患者に対するアセスメントの内容として、適切なのはどれか。２つ選べ。

1　患者はすでに肝硬変に移行しているため、B 型肝炎に対する治療薬は不要である。

2　利尿薬による過度の脱水は、高アンモニア血症を悪化させる可能性がある。

3　肝臓は正常に機能している。

4　酸化マグネシウム錠とラクツロースゼリー分包の併用により下痢の可能性がある。

5　分岐鎖アミノ酸は、配合経口ゼリー剤が処方されているので、食事による摂取が不要である。

問 304-305　薬剤師が病院薬剤部内の勉強会で、ナトリウム-グルコース共輸送体２（SGLT2 ）阻害薬Ａの心保護作用について発表することになり、以下の文献を入手した。

動脈硬化性心疾患を有する、または、動脈硬化性心疾患リスクが高い２型糖尿病患者を対象に、「Ａ投与群」、「Ａ非投与群」の２群に無作為に割り付けし、心血管死または心不全による入院を主要評価項目として検討したところ、以下の結果を得た。

Outcome	Ａ投与群 (N ＝ 8,582)		Ａ非投与群 (N ＝ 8,578)		Hazard Ratio (95%信頼区間)
	人（%）	発生率	人（%）	発生率	
Cardiovascular death or hospitalization for heart failure	417（4.9）	12.2	496（5.8）	14.7	0.83（0.73-0.95）

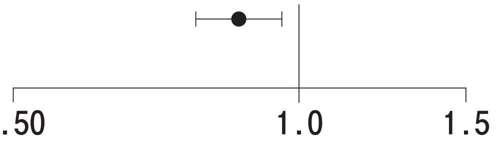

発生率は 1,000 人年[(注)] 当たりの発生数を示す。

（注） １人を１年間観察した場合、１人年に相当する。

New England Journal of Medicine, 380, 347-357, 2019 より一部抜粋・修正

問 304（病態・薬物治療）

この解析に用いられた統計手法として、適切なのはどれか。**1つ**選べ。

1　t 検定

2　Mann-Whitney の U 検定

3　Kruskal-Wallis 検定

4　Cox 回帰分析

5　重回帰分析

問 305（実務）

薬剤師が勉強会で説明する内容として、正しいのはどれか。**2つ**選べ。

1　主要評価項目は、代用エンドポイントを用いています。

2　ハザード比の 95％信頼区間が 1 を含んでいないことから、両群間に統計学的に有意差が認められます。

3　相対リスク減少は 83％です。

4　A の投与は、2 型糖尿病患者において主要評価項目のリスクを減少させるといえます。

5　A の投与は、2 型糖尿病患者において心血管死、心筋梗塞、虚血性脳卒中の心血管イベントの複合項目のリスクを減少させるといえます。

問 306-307　60 歳男性。息切れ、倦怠感が継続するため検査したところ、フィラデルフィア染色体陰性の急性リンパ性白血病と診断され、以下の化学療法を施行した。担当薬剤師が患者から「手や足がピリピリとしびれ、物がつかみづらい」との訴えを受け、副作用が疑われた。

医薬品名・投与量	投与経路・投与時間	投与日
シクロホスファミド水和物注射用<br>1,200 mg/m^2 ＋ 生理食塩液 250 mL	点滴静注（３時間）	１日目
ダウノルビシン塩酸塩注射用 60 mg/m^2 ＋<br>生理食塩液 100 mL	点滴静注（１時間）	１日目
ビンクリスチン硫酸塩注射用 1.3 mg/m^2<br>＋ 生理食塩液 50 mL	点滴静注（10 分）	８、15、22 日目
L-アスパラギナーゼ注射用 3,000 IU/m^2 ＋<br>５％ブドウ糖注射液 500 mL	点滴静注（２時間）	９、11、13、16、<br>18、20 日目
プレドニゾロン錠 60 mg/m^2（１日量）	１日２回　朝昼食後	１～21 日目以後<br>漸減

問 306（実務）

この化学療法で用いられた医薬品のうち、この患者が訴えた症状を引き起こす可能性のあるのはどれか。**１つ**選べ。

1　シクロホスファミド
2　ダウノルビシン
3　ビンクリスチン
4　L-アスパラギナーゼ
5　プレドニゾロン

問 307（法規・制度・倫理）

その後、この患者は歩行困難となり、化学療法による副作用と疑われた。この場合の薬剤師の対応として、正しいのはどれか。2つ選べ。

1 副作用が疑われる医薬品の製造販売業者に副作用の可能性があることを報告した。

2 副作用が疑われる医薬品の製造販売業者に副作用の治療費を請求した。

3 適正に使用された医薬品は、全て副作用被害救済制度の対象になるので、申請を患者に勧めた。

4 医薬品と副作用の因果関係が明確ではなかったが、独立行政法人医薬品医療機器総合機構（PMDA）へ報告した。

5 副作用が疑われる医薬品について、病院のホームページにて患者情報を公開して、注意喚起を行った。

問 308-309　30 歳女性。「この季節になると、くしゃみが出て、鼻が詰まってつらい」との訴えがあり、仕事や家事などで忙しいため、市販薬で対処したいと、薬局に相談に来た。そこで、薬剤師は要指導医薬品のフルチカゾンプロピオン酸エステル点鼻薬を勧めた。

問 308（実務）

当該医薬品を販売する前に、薬剤師が確認する内容として、最も適切なのはどれか。1つ選べ。

1　光線過敏症と診断されたことがあるか。
2　鼻水の色が黄色く鼻腔に痛みがあるか。
3　狭心症、心筋梗塞と診断されたことがあるか。
4　貧血と診断されたことがあるか。
5　危険を伴う機械操作をしているか。

問 309（法規・制度・倫理）

この女性は当該医薬品の購入を希望した。販売時の薬剤師の対応として、正しいのはどれか。２つ選べ。

1　女性に情報提供及び指導を行い、その内容を理解したこと、質問がないことを確認した。

2　母親も同様の症状があるが、仕事で忙しく来局できないとのことだったため、母親の分も販売した。

3　この医薬品は、インターネットでも購入可能であると説明した。

4　仕事が忙しく何回も購入することが面倒とのことだったので、10 箱をまとめて販売した。

5　販売した薬剤師の氏名、薬局の名称、連絡先を女性に伝えた。

問 310-311　20 歳男性。一人暮らし。1ヶ月前に風邪をひいてから、体調が不良となった。現在、治療中の疾患はない。健康サポート薬局の表示を見て来局した。対応した薬剤師は、この男性の自覚症状（口喝、多尿、急激な体重減少、疲労感）などの訴えを聞いて糖尿病の疑いがあると判断して、医療機関への受診勧奨を行った。

問 310（法規・制度・倫理）

「健康サポート薬局」を表示するための基準として、適切なのはどれか。**２つ**選べ。

1　患者の代わりに、医療機関の受診の予約を行う体制を有している。

2　いつでも相談に対応できるように 24 時間開局している。

3　個人情報に配慮した相談窓口を有している。

4　要指導医薬品や介護用品について、助言できる体制を有している。

5　薬局内に無菌調剤室を設置している。

問 311（実務）

この男性は医療機関で１型糖尿病と診断され、血糖コントロールのため入院し、以下の処方で治療を開始することになった。

（処方）

ノボラピッド 50 ミックス注フレックスペン（注）　１本（３mL）

１回４単位　１日２回　朝夕食直前

（注）成分名：インスリンアスパルト（遺伝子組換え）（300 単位/mL）

病棟担当薬剤師がこの男性に指導する内容として、適切なのはどれか。２つ選べ。

1　本製剤は、開封後に常に冷蔵庫で保存する。
2　使用する前に、本製剤を振らないようにする。
3　腹部の部位を決め、なるべく前回注射した場所に注射する。
4　低血糖症状が起きたら、糖分を摂るようにする。
5　注射後には、必ず食事を摂るようにする。

問 312-313　24歳女性。病院の婦人科を受診後、以下の処方箋を持参し来局した。

（処方）

レボノルゲストレル錠1.5 mg　1回1錠（1日1錠）

1日1回　1日分

問 312（実務）

この患者への薬剤師の説明として、適切なのはどれか。**1つ**選べ。

1　この薬は今すぐ服用してください。

2　この薬を服用しても、月経周期や出血の状況に影響はありません。

3　この薬を服用すると、性感染症の発症を予防できます。

4　この薬を服用すると、完全に妊娠を回避できます。

5　この薬は計画的な避妊にも用いられます。

問 313（法規・制度・倫理）

この診療は保険外診療である。この処方箋に記載されていなければならないのはどれか。２つ選べ。

1 患者の年齢
2 保険者番号
3 病院の所在地
4 専門医の資格
5 処方理由

問 314-315　40 歳男性。以下の処方箋を持って来局した。今回、初めての来局で、面談したところ、患者はこれまでも同じ薬を他の薬局で調剤してもらい服用しているとのことであった。この薬局では初回の調剤であったので、薬剤師は調剤した日の 14 日後に、この患者に電話をして服薬状況などについて確認した。

その際、実は、服用回数・服用錠数が多くて面倒だと感じていることが判明した。また、患者から、これまでも同じ薬を飲んでいるので、薬の変更がない場合には薬の説明やその説明書、薬袋は不要であるとの申し出があった。

（処方）

メトホルミン塩酸塩錠 250 mg	1 回 1 錠（1 日 3 錠）		
	1 日 3 回	朝昼夕食後	30 日分
オルメサルタン口腔内崩壊錠 10 mg	1 回 1 錠（1 日 1 錠）		
	1 日 1 回	朝食後	30 日分
アトルバスタチン口腔内崩壊錠 10 mg	1 回 1 錠（1 日 1 錠）		
	1 日 1 回	夕食後	30 日分

問 314（実務）

この患者の「服用回数・服用錠数が多くて面倒だと感じている」ことに関して、アドヒアランスに懸念があると考えた薬剤師の対応として、適切なのはどれか。**2つ**選べ。

1　医師に対し、服薬情報提供書を用いて、アドヒアランスに懸念があることを情報提供した。

2　医師に電話で照会を行い、前回の処方について遡って処方内容を変更してもらった。

3　患者に対し、服用を忘れた場合は、次回服用時に 2 回分まとめて服用するよう指導した。

4　患者に対し、次回受診時に残薬調整をするので、余った薬を持参するように指導した。

5　患者に対し、次回受診時までは、患者の判断で服用量を調整して服用すれば良いと指導した。

問 315（法規・制度・倫理）

この患者が次回来局した際に、患者の申し出に対する薬剤師の対応として、適切なのはどれか。<u>２つ</u>選べ。

1　薬剤の安全使用などのために必要であることから、薬袋、服薬指導、薬剤の説明書についてのルールが法令で定められていることを説明した。

2　今回から薬剤は、表示のない無地の袋にまとめて交付することとした。

3　前回と処方内容が変わらない場合には服薬指導を省略することとした。

4　服薬指導と薬剤の説明書の交付の両方を省略するためには医師への疑義照会が必要であると説明した。

5　服薬指導は薬剤の説明書（電磁的な記録も含む）を用いて行う必要があることを説明した。

問 316-317　85 歳男性。肺がんで入院治療を行っていたが、在宅で緩和ケアを受けることになり退院した。痛みに対して、アセトアミノフェン錠が投与されていたが、先日から痛みが増してきたので、オピオイドが処方されることになった。終末期のため患者家族が服薬について管理している。現在の処方を以下に示す。

（処方１）

オキシコドン徐放錠 5 mg	1 回 1 錠（1 日 2 錠）
	1 日 2 回　12 時間毎に投与　14 日分

（処方２）

オキシコドン塩酸塩水和物散 2.5 mg	1 回 1 包
	痛いとき 20 回分（20 包）

（処方３）

酸化マグネシウム錠 250 mg	1 回 1 錠（1 日 3 錠）
	1 日 3 回　朝昼夕食後　14 日分

（処方４）

プロクロルペラジンマレイン酸塩錠 5 mg	1 回 1 錠（1 日 3 錠）
	1 日 3 回　朝昼夕食後　14 日分

問 316（実務）

患者家族への服薬指導として、適切なのはどれか。<u>２つ</u>選べ。

1　痛みが強い時は、効果をあげるために処方１の薬剤をかみ砕いて服用してください。

2　便に錠剤の一部が排泄されていたら、鎮痛効果が弱まるので、処方２の薬剤を１回分服用してください。

3　処方２の薬剤を追加服用する場合は、５時間以上あけてください。

4　便秘になる可能性があるので、処方３が処方されています。

5　吐き気がおきる可能性があるので、処方４が処方されています。

問 317（法規・制度・倫理）

この患者家族が、在宅で調剤済みのオキシコドンを管理する場合、麻薬及び向精神薬取締法に照らし合わせ、正しい説明はどれか。**1つ**選べ。

1　「家庭麻薬」として管理する。

2　かぎのかかる堅固な保管庫での保管が必要である。

3　管理者を決めて病院又は薬局に届け出る必要がある。

4　医師の許可があれば海外旅行に携帯できる。

5　不要となった残薬は調剤した薬局に返却できる。

問 318-319　75歳女性。一人暮らし。数年来処方１で治療していた。１ヶ月前に家の廊下で転倒し腰を痛め、痛くて眠れないとの訴えがあったため、処方２が追加となった。本日の服薬指導時に患者は「腰はもう治り、痛みもない。夜もよく眠れるようになってよかった。しかし、腰を打ってからほとんど家で横になっている。食欲がなくて、水もあまり飲んでいないためか、トイレに行く回数が減っている。」と話していた。また、薬剤師は会話中に患者の手が震えており、両下肢にむくみがあることに気づいた。なお、血圧は正常にコントロールできている。

（処方１）

アムロジピン錠 5 mg	1回1錠（1日1錠）		
	1日1回	朝食後	30日分
酸化マグネシウム錠 250 mg	1回1錠（1日3錠）		
	1日3回	朝昼夕食後	30日分
メトクロプラミド錠 5 mg	1回1錠（1日3錠）		
	1日3回	朝昼夕食前	30日分

（処方２）

ロキソプロフェン Na 錠 60 mg	1回1錠（1日3錠）		
	1日3回	朝昼夕食後	30日分
ゾルピデム酒石酸塩錠 5 mg	1回1錠（1日1錠）		
	1日1回	就寝前	30日分

問 318（実務）

医師への処方見直しや確認の提案に向け、薬剤師がアセスメントする項目として、適切なのはどれか。2つ選べ。

1　アムロジピン錠の長期服用による乏尿
2　酸化マグネシウム錠の長期服用による錐体外路症状
3　メトクロプラミド錠の長期服用による錐体外路症状
4　ロキソプロフェン Na 錠による腎機能低下
5　ゾルピデム酒石酸塩錠による乏尿

問 319（法規・制度・倫理）

このように、追加された処方薬が漫然と継続されることで、薬剤の副作用や重複投与の可能性、さらには、服用できない薬剤の増加による残薬の問題など、ポリファーマシーに関連した様々な課題が発生している。これまで、ポリファーマシーの対策として、国が実施した施策はどれか。2つ選べ。

1　「高齢者の医薬品適正使用の指針」の作成を行った。
2　ポリファーマシーによる問題がある患者数を半減するとの数値目標を設定した。
3　医療保険上の処方箋に記載できる薬剤の剤数の上限を設けた。
4　患者の服用する薬剤を減らした場合の取組みについて診療報酬で評価した。
5　同時に使用する薬剤の剤数が 10 を超えた分の薬剤費の自己負担割合を増やした。

問 320-321　62 歳男性。パーキンソン病にて治療をしていたところ、症状が進行し嚥下が困難になったので、経管投与が開始となった。この患者の妻が薬局に以下の処方箋を持参した。処方箋を受け取った薬剤師は、医師に簡易懸濁法で投与することを提案したところ受け入れられた。薬剤師は、妻に簡易懸濁法による投与方法について指導することにした。なお、今回の処方薬はすべて簡易懸濁法により投与可能である。

（処方 1 ）

ビペリデン塩酸塩錠 1 mg	1 回 1 錠（1 日 2 錠）	
	1 日 2 回　朝夕食後	14 日分

（処方 2 ）

レボドパ・ベンセラジド塩酸塩錠	1 回 1 錠（1 日 3 錠）	
	1 日 3 回　朝昼夕食後	14 日分

（処方 3 ）

ランソプラゾールカプセル 15 mg	1 回 1 カプセル（1 日 1 カプセル）	
	1 日 1 回　朝食後	14 日分

問 320（実務）

この患者の妻に対する薬剤師の指導の内容として、最も適切なのはどれか。**1 つ**選べ。

1　朝は処方 1 ～ 3 までの薬剤を、夕は処方 1 と 2 の薬剤を、まとめて懸濁してください。

2　処方 3 の薬剤はカプセルを外してから、懸濁してください。

3　処方 1 と 2 の薬剤は粉砕してから、懸濁してください。

4　懸濁には、90 ℃以上の熱いお湯を用いてください。

5　薬剤が溶解したのを確認してから、投与してください。

問 321（法規・制度・倫理）

その後、介護が大変になったと妻より相談があり、薬剤師が介護保険について情報提供することとした。薬剤師の説明として、正しいのはどれか。2つ選べ。

1　ご主人の疾患の場合は 65 歳にならなくても介護保険が申請できます。

2　申請書類は薬局に提出してください。

3　要介護の認定は、心身の状態と主治医の意見をもとに判定されます。

4　要介護状態は、要介護 1 と 2 の 2 つに区分されています。

5　要介護認定を受けた場合は、介護保険と医療保険のどちらを適用するかは、薬局と患者の相談で決めます。

問 322-323　60 歳代の男性が薬剤師会が主催する健康相談会にやってきた。相談内容は以下のとおりである。

「昔一緒に働いていた友人が悪性中皮腫っていうがんになった。アスベスト（石綿）が原因だと聞いた。最近、自分も同じような症状がでてきて心配だ。」

問 322（実務）

アスベスト（石綿）による健康被害を疑った薬剤師の対応として、適切なのはどれか。**２つ**選べ。

1　潜伏期間が比較的短いため、友人と働いていた時期がいつなのかを確認した。

2　友人と働いていた場所が、工事現場などの曝露のおそれがある場所なのか確認した。

3　呼吸困難、咳、胸痛などの自覚症状があるか確認した。

4　飛沫感染によって他人にうつすおそれがあることを説明した。

5　吸い込んだアスベスト（石綿）による一過性の炎症反応のため、心配いらないと説明した。

問 323（法規・制度・倫理）

この男性に医療機関への受診勧奨を行ったところ、「今でも年金でぎりぎりの生活をしているのに治療費まで出せないよ」と言われ、薬剤師は、「アスベストが原因での病気の治療は、公費負担医療制度の対象になる可能性がある」と説明した。公費負担医療制度の内容として、正しいのはどれか。２つ選べ。

1　税金を基礎として医療費給付を行う。
2　高額な医療が必要と判断された場合に利用する。
3　国や地方自治体が運用する。
4　社会保険方式による制度である。
5　保険薬局であれば、どこでも取り扱うことができる。

問 324-325　63 歳女性。10 年ほど前から股関節の痛みを感じ、整形外科を受診し変形性股関節症と診断された。処方 1 の薬剤の服用で様子を見ていたが、症状が悪化し、杖なしでは歩けなくなったため、医師から人工股関節置換術を勧められ入院して手術を受けることになった。患者が入院する病院は Diagnosis Procedure Combination（DPC）制度対象病院で、手術後から処方 2 の薬剤を服用予定である。

（処方 1 ）

ロキソプロフェン Na 錠 60 mg	1 回 1 錠（ 1 日 2 錠）
	1 日 2 回　朝夕食後　14 日分

（処方 2 ）

エドキサバントシル酸塩水和物錠 30 mg	1 回 1 錠（ 1 日 1 錠）
	1 日 1 回　朝食後　　14 日分

問 324（実務）

この患者への処方 2 に関する服薬指導として、適切なのはどれか。**２つ**選べ。

1　手術後に傷口の血の流れをよくして治りを早くする薬です。
2　この薬は人工股関節置換術後に起こる合併症の予防のために使用します。
3　手術後病室に戻ったらすぐに服用を始めてもらいます。
4　この薬の服用中はグレープフルーツジュースを服用しないでください。
5　あざができたり、歯ぐきから出血したら、すぐに教えてください。

問 325（法規・制度・倫理）

服薬指導の際に、患者から入院費用について質問を受けた。手術を受ける病院での公的医療保険制度での医療費の支払いに関する説明として、正しいのはどれか。２つ選べ。

1　手術前に患者が希望すれば、医療費の支払いを全額出来高払いに変更できる。

2　人工股関節置換術の入院基本料は医療費の包括払いのため入院日数の上限が決まっている。

3　入院中に服用する処方１と処方２の薬剤費は医療費の包括払いに含まれている。

4　退院後のリハビリテーション料は医療費の包括払いに含まれている。

5　個室への入院を希望する場合は差額ベッド代が必要になる。

一般問題（薬学実践問題）【実務】

問 326 67 歳女性。身長 156 cm、体重 52 kg。2 型糖尿病、持続性心房細動及び高血圧症で自宅近くの病院に通院し、以下の薬剤を服用している。15 日前の定期受診時の血清クレアチニン値は 0.92 mg/dL、eGFR 47.0 mL/min/1.73 m^2 であった。気温 30 ℃以上の中、庭の草刈りを行っていたところ、頭痛とめまいの症状が出てきたため、今回来院した。そこで、脱水症と診断され、入院して酢酸リンゲル液を投与することとなった。

（処方）

メトホルミン塩酸塩錠 250 mg	1 回 1 錠（1 日 2 錠）
	1 日 2 回　朝夕食後　30 日分
オルメサルタン口腔内崩壊錠 20 mg	1 回 1 錠（1 日 1 錠）
ビソプロロールフマル酸塩錠 2.5 mg	1 回 1 錠（1 日 1 錠）
リバーロキサバン錠 10 mg	1 回 1 錠（1 日 1 錠）
ランソプラゾール口腔内崩壊錠 15 mg	1 回 1 錠（1 日 1 錠）
	1 日 1 回　朝食後　30 日分

（来院時所見）

意識は清明。体温 36.8 ℃、血圧 128/80 mmHg、脈拍数 108 拍/分、
尿量 20 mL/h

（検査値）

赤血球数 359 × 10^4/μL、Hb 13.4 g/dL、Ht 40%、白血球数 3,700/μL
血小板数 17 × 10^4/μL、BUN 53 mg/dL、血清クレアチニン値 1.2 mg/dL
eGFR 35.1 mL/min/1.73 m^2、Na 145 mEq/L、K 5.6 mEq/L、Cl 105 mEq/L

この患者の持参薬のうち、薬剤師が入院時に服用中止を提案する薬剤として、適切なのはどれか。２つ選べ。

1　メトホルミン塩酸塩錠
2　オルメサルタン口腔内崩壊錠
3　ビソプロロールフマル酸塩錠
4　リバーロキサバン錠
5　ランソプラゾール口腔内崩壊錠

問 327　67 歳男性。身長 160 cm、体重 50 kg。パフォーマンスステータス（PS）０、非ホジキンリンパ腫に対する全身化学療法として、リツキシマブとベンダムスチンの併用療法を検討している。本治療開始前に、薬剤師が確認又は提案する事項として、適切なのはどれか。<u>２つ</u>選べ。

（投与量）

リツキシマブ（遺伝子組換え）：(375 mg/m^2）Day 1

ベンダムスチン塩酸塩：(90 mg/m^2）Day 2 、 3

1 サイクル 28 日

（検査値）

総ビリルビン 1.0 mg/dL、AST 21 IU/L、ALT 17 IU/L、BUN 22.9 mg/dL、
血清クレアチニン値 1.2 mg/dL、赤血球数 216 × 10^4/μL、
白血球数 3,750/μL、好中球数 1,880/μL、Hb 11.3 g/dL、
血小板数 21.4 × 10^4/μL

1　前投与薬として抗ヒスタミン剤、解熱鎮痛剤が処方されていることを確認する。

2　リツキシマブの減量を提案する。

3　ベンダムスチン塩酸塩の減量を提案する。

4　*EGFR* 遺伝子変異が陽性であることを確認する。

5　腫瘍崩壊症候群のリスク評価の実施を提案する。

問 328　45 歳女性。身長 152 cm、体重 40 kg。アルコールの慢性的な大量摂取に伴う慢性肝炎治療のため、これまで入退院を繰り返してきた。今回、Child-Pugh 分類でB（８点）の肝硬変と診断され、治療目的のため入院となった。

（入院時の検査値等）

脳症Ⅱ度、腹水 2 L、総ビリルビン 2.5 mg/dL、血清アルブミン 3.0 g/dL、
PT-INR 2.0、AST 85 IU/L、ALT 80 IU/L、γ-GTP 21 IU/L、
アンモニア 420 μg/dL、血清クレアチニン値 0.7 mg/dL、
eGFR 71.0 mL/min/1.73 m^2、Na 142 mEq/L、K 4.8 mEq/L

（入院時の持参薬）

レボカルニチン内用液
プレドニゾロン錠
ウルソデオキシコール酸錠
ラクツロースシロップ
レバミピド錠

脳症及び腹水貯留の改善が思わしくないことから、追加を提案する薬剤として、適切なのはどれか。**２つ**選べ。

1　ラクチトール水和物散
2　バンコマイシン散
3　リファキシミン錠
4　トルバプタン錠
5　アセタゾラミド錠

問 329　45 歳女性。人間ドックで膵がんの疑いを指摘され、大学病院を受診し、検査の結果、膵がん（遠隔転移あり）と診断された。1 次化学療法として FOLFIRINOX 療法による治療を開始するにあたり、遺伝子多型を検査したところ、*UGT1A1*6* ヘテロ接合体であった。初回投与（1 コース目）は以下の投与量で実施した。その後、下痢（1 日 2 回程度）が見られたが、止瀉薬を内服することで対応可能であった。しかし、血液検査の結果、2 コース目の化学療法は 1 週間延期された。

（1 コース目投与量）

FOLFIRINOX：オキサリプラチン点滴静注（85 mg/m^2）
イリノテカン塩酸塩水和物点滴静注（180 mg/m^2）
フルオロウラシル急速静注（400 mg/m^2）
フルオロウラシル持続静注（2,400 mg/m^2）
レボホリナートカルシウム水和物点滴静注（200 mg/m^2）

（2 コース目投与予定日の血液検査値）

総ビリルビン 1.1 mg/dL、AST 24 IU/L、ALT 22 IU/L、BUN 22.9 mg/dL、
血清クレアチニン値 0.9 mg/dL、赤血球数 270 × 10^4/μL、
白血球数 1,690/μL、好中球数 820/μL、Hb 12.2 g/dL、
血小板数 21.4 × 10^4/μL

（2 コース目投与予定日から 1 週間延期した日の血液検査値）

総ビリルビン 0.8 mg/dL、AST 27 IU/L、ALT 23 IU/L、BUN 18.5 mg/dL、
血清クレアチニン値 0.5 mg/dL、赤血球数 355 × 10^4/μL、
白血球数 4,230/μL、好中球数 1,910/μL、Hb 13.6 g/dL、
血小板数 28.6 × 10^4/μL

２コース目投与予定日から１週間延期した日の血液検査結果をもとに、カンファレンスを実施した。薬剤師が医師に提案する内容として、最も適切なのはどれか。**1つ**選べ。

1　イリノテカン塩酸塩水和物を減量して投与する。

2　レボホリナートカルシウム水和物を減量して投与する。

3　化学療法当日に人血小板濃厚液を投与し、イリノテカン塩酸塩水和物は初回と同量で投与する。

4　化学療法当日にG-CSF製剤を投与し、イリノテカン塩酸塩水和物は初回と同量で投与する。

5　今回も化学療法は延期する。

問 330　48 歳女性。体重 60 kg。原因不明の急性腎不全で入院し、腹膜透析導入となった。腹膜透析カテーテル挿入術を終え、入院 3 日目より透析液 A で腹膜透析を開始した。入院 10 日目に除水効果のより高い透析液 B に変更して 30 分ほど経過した時点で、両下肢に広範囲にわたる皮疹と呼吸困難をともなう急激な血圧低下を認めた。なお、入院の 10 日前より開始したスクロオキシ水酸化鉄チュアブル錠の服用を継続している。

透析液の組成

成分	透析液 A	透析液 B
イコデキストリン（g/L）	－	75
ブドウ糖（g/L）	38.6	－
塩化ナトリウム（g/L）	5.38	5.35
乳酸ナトリウム（g/L）	4.48	4.48
塩化カルシウム（g/L）	0.183	0.257
塩化マグネシウム（g/L）	0.051	0.051
浸透圧（理論値）（mOsm/L）	483	282

両下肢の皮疹を認めた直後に調べた血液検査で好酸球数が増加していた。主治医から薬剤師への相談に対し、今回の有害事象に関連する提案として、正しいのはどれか。**２つ**選べ。

1　スクロオキシ水酸化鉄に対するアレルギー反応が疑われるため、一時的に同薬剤の使用を中止する。
2　透析液変更による反応と考えられることから、透析液 B にブドウ糖を追加して浸透圧を透析液 A と合わせる。
3　透析液 B のイコデキストリンに対するアレルギー反応が疑われるため、透析液 B から A に再度変更する。
4　アドレナリンを静注する。
5　トシリズマブを静注する。

問 331　63 歳男性。体重 58 kg。頻脈性不整脈を契機に発症したうっ血性心不全に対し、先月より開始した処方１に処方２が今回追加されることとなり、３日後と８日後の診察が予約された。処方２開始当日朝の検査値等は以下のとおりであった。

（処方１）

ピルシカイニド塩酸塩カプセル 50 mg	1 回 1 錠（1 日 3 錠）
	1 日 3 回　朝昼夕食後　30 日分

（処方２）

ジゴキシン錠 0.25 mg	1 回 1 錠（1 日 1 錠）
スピロノラクトン錠 25 mg	1 回 1 錠（1 日 1 錠）
	1 日 1 回　朝食後　30 日分

（検査値及び所見）

血清中ピルシカイニド濃度 0.5 μg/mL、
クレアチニンクリアランス 90 mL/min、
K 4.0 mEq/L、血圧 140/92 mmHg、心電図 異常なし

ピルシカイニド、ジゴキシンの有効血中濃度域は、それぞれ 0.2〜0.9 μg/mL、0.5〜1.5 ng/mL、本患者における消失半減期はそれぞれ５時間、36 時間とする。

以下の記述のうち、適切でないのはどれか。**1つ**選べ。

1　ジゴキシン追加にあたり、患者の消化器症状に注意する。
2　３日後の診察時にジゴキシンの治療薬物モニタリング（TDM）を実施する。
3　スピロノラクトンが併用されているためジゴキシンの血中濃度上昇に注意を払う。
4　ピルシカイニドの TDM は月に１回程度とする。
5　血清カリウム濃度の上昇に注意する。

問 332　43 歳女性。入院精査の結果、右乳がんのため、術前化学療法として AC 療法が開始となった。

（AC 療法）

ドキソルビシン塩酸塩

シクロホスファミド注射液

３週間に１回点滴投与

（支持療法）

以下の３剤を抗がん薬投与前に点滴静注

ホスアプレピタント注射液

デキサメタゾンリン酸エステルナトリウム注射液

グラニセトロン塩酸塩注射液

外来化学療法室の担当薬剤師が２コース目の治療開始前に患者と面談し、１コース目終了後の有害反応として、確認すべき症状はどれか。２つ選べ。

1　口内炎

2　霧視

3　爪囲炎

4　嘔気

5　皮膚の乾燥

問 333　33 歳男性。B 型慢性肝炎治療中。交通事故で病院に搬送された。この患者の血液で汚染された物品等と、消毒に適用されるものの組合せとして、正しいのはどれか。２つ選べ。

	物品等	消毒薬
1	医療用拡大鏡レンズ	クロルヘキシジングルコン酸塩
2	手術用器具	グルタラール
3	病室の床	次亜塩素酸ナトリウム
4	処置室の流し台	ポビドンヨード
5	搬送用ストレッチャー	ベンザルコニウム塩化物

問 334　精神神経科医師より統合失調症治療薬パリペリドンパルミチン酸エステルを処方したいので採用してほしいとの申請が医薬品情報室に提出された。本剤については、過去に安全性速報が発出されている。そこで、本剤の採用にあたり、医薬品情報担当薬剤師による対応として、適切でないのはどれか。**1つ**選べ。

1　直ちに精神神経科に安全性速報の内容を情報提供し、追って院内にも周知するよう努める。

2　パリペリドンパルミチン酸エステル水懸筋注が処方される患者・家族に安全性速報の内容を含めて説明する。

3　調剤室の掲示板に目立つように安全性速報を掲示し、調剤業務に役立てる。

4　急激な精神興奮などの治療を要する患者への使用に限るよう院内に周知する。

5　リスペリドン持効性懸濁注射液を使用中の患者リストを作成の上、医師と共有し、パリペリドンパルミチン酸エステル水懸筋注への今後の変更について検討する。

問 335　原疾患に対して用いられる薬物が、併発症の欄に記載されている疾患を有する場合であっても使用可能なのはどれか。**1つ**選べ。

	原疾患	薬物	併発症
1	高血圧症	カルベジロール	気管支ぜん息
2	胃痙れん	チキジウム臭化物	閉塞隅角緑内障
3	統合失調症	クエチアピンフマル酸塩	糖尿病
4	下痢症	ベルベリン塩化物	低カリウム血症
5	過活動膀胱	ミラベグロン	重篤な心疾患

問 336　3歳男児。家族で登山に行き、大量の汗をかいた。当日の夕方に帰宅後、首の周りや額に赤みを伴った小さな丘疹が現れた。以前も汗を大量にかいた後には首の周りに同様の症状が現れたことがあり、今回も赤くなった部位に同時に強いかゆみも出現したため父親が近隣の薬局を訪れて相談した。

相談された薬剤師は、男児にアレルギー歴がないことを確認し、店頭にある一般用医薬品の外用薬の中から今回の症状を緩和させる医薬品を選択した。選択した医薬品の成分として、最も適切なのはどれか。**1つ**選べ。

1　アシクロビル
2　ジフェンヒドラミン塩酸塩、酸化亜鉛、グリチルレチン酸
3　ナイスタチン、クロラムフェニコール、フラジオマイシン硫酸塩
4　インドメタシン、トコフェロール酢酸エステル、アルニカチンキ
5　ベタメタゾン吉草酸エステル

問 337　月齢５ヶ月、体重 7.4 kg の患児に以下の処方が出された。

（処方）

アセトアミノフェン坐剤小児用[(注)] 50 mg　１回 1.5 個

発熱時（38.5 ℃以上）　４回分（全８個）

（注）添加物はハードファット

患児の保護者への坐薬の使用方法の説明として、適切なのはどれか。<u>２つ</u>選べ。

1　固くなるので、冷蔵庫は避けて部屋の棚の中などで保管してください。

2　容器から坐剤を取り出した後、図１のように先端から肛門内に深く挿入してください。

3　半分にする場合は、図２のように切ってください。

4　挿入してから４〜５秒、肛門部をティッシュ等で押さえてください。

5　２時間経過しても効果がない場合は、すぐに１回分を追加してください。

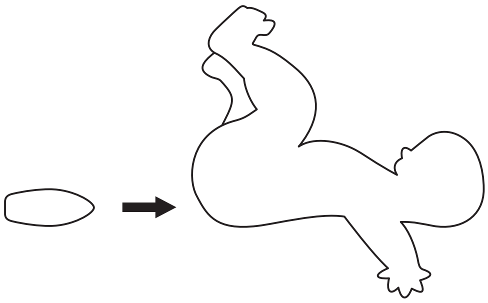

図 1

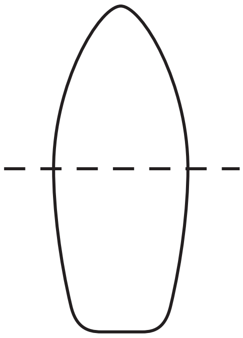

図 2

問 338　10 歳男児。体重 30 kg。１週間前より微熱と咳嗽があり、近医を受診した。感冒との診断を受け、咳止めと解熱剤を処方されて帰宅した。２、３日前より咳嗽と喀痰が次第に強くなり、微熱も持続するため再度受診し胸部レントゲン検査を実施したところ炎症所見が観察され、大学病院へ搬送入院となった。入院後 PCR 検査の結果、マイコプラズマ肺炎の診断を受けた。以下が入院日の処方である。

（処方１）

クラリスロマイシンシロップ用 10%	１回 1.0 g（１日 3.0 g）
	１日３回　朝昼夕食後　３日分

（処方２）

カルボシステインドライシロップ用 50%	１回 0.6 g（１日 1.8 g）
	１日３回　朝昼夕食後　３日分

（処方３）

サルブタモール硫酸塩吸入液 0.5%	１回 0.3 mL（１日 1.8 mL）
	１日６回　４時間毎　ネブライザー吸入

薬剤師が訪室し、母親に服薬指導を実施することになった。服薬指導する内容として、適切なのはどれか。２つ選べ。

1　処方１を内服する場合、水で飲めない場合にはジュースやヨーグルトでもよい。

2　処方１と処方２を一緒に内服すると苦味が強くなる可能性があるため、間隔をあけて飲ませる。

3　処方２は、痰が出なくなったら中止してもよい。

4　処方３を吸入中は、顔色や呼吸の様子に注意を払い、気分が悪くなったり呼吸が苦しくなったりしたら中止する。

5　処方３の１回の吸入終了後も、咳がひどいようであれば、直ちに２回分吸入を実施してもよい。

問 339　次の薬物について、治療薬物モニタリング（TDM）を実施する上での適切な採血のタイミングと、中毒域に達した場合に起こり得る副作用（中毒症状）の組合せとして、適切なのはどれか。２つ選べ。

	薬物<br>(疾患等)	採血の<br>タイミング	副作用<br>(中毒症状)
1	タクロリムス水和物<br>(腎移植)	トラフ	腎障害
2	テイコプラニン<br>(敗血症)	ピーク	肝障害
3	ボリコナゾール<br>(肺アスペルギルス症)	ピーク	肝障害
4	バルプロ酸ナトリウム<br>(てんかん)	トラフ	意識障害
5	メトトレキサート<br>(骨肉腫)	トラフ	骨髄抑制

問 340　6月に健康サポート薬局の薬剤師が地域住民を対象に健康相談会を開催したところ、参加者から食中毒に関する質問が多く寄せられた。その質問に対して薬剤師は衛生管理を含めた助言を行った。その内容として、適切なのはどれか。**2つ**選べ。

1　下痢が続いている間は、経口補水液の摂取を控えてください。

2　鮮度が落ちたサバ等の青魚を食べると、じん麻疹などのアレルギー症状がでるので注意してください。

3　サルモネラ菌による食中毒を予防するため、十分に加熱調理しましょう。

4　腸管出血性大腸菌 O157 は、食品を一度冷凍・自然解凍することで死滅させることができます。

5　特に夏季はノロウイルスが原因となる食中毒が多いので、貝類を食べるのは控えてください。

問 341　以下の処方により調製された薬剤を鑑査するにあたり、分包紙の重さを含む薬剤の全量を秤量した時の正しい重量はどれか。**1つ**選べ。ただし1包量が 0.1 g 以下の場合は、1包あたり 0.2 g の乳糖を賦形することとし、分包紙は 0.5 g/包とする。

（処方）

ロートエキス散 10%　1回 10 mg（1日 20 mg）【原薬量】
1日2回　朝夕食後　14 日分

1　15.4 g
2　16.8 g
3　19.6 g
4　21.6 g
5　22.4 g

問 342　8歳女児、体重 30.0 kg。発熱のため近医を受診し、以下の内容の処方箋を薬局に持参した。母親が水剤を希望したため処方医に相談し、アセトアミノフェンシロップ小児用2%へ変更となった。調剤時に計量するアセトアミノフェンシロップ小児用2%の全量として、正しいのはどれか。**1つ**選べ。

（変更前の処方）

アセトアミノフェンドライシロップ小児用 20%　1回 1.5 g
発熱時　5回分

1　7.5 mL
2　15 mL
3　45 mL
4　75 mL
5　150 mL

問 343　65 歳男性。意識障害により経口摂取困難となったため、非経口投与による栄養管理を開始することになった。主治医より高カロリー輸液の処方設計の依頼があり、以下の処方提案をした。この高カロリー輸液の非タンパク質カロリー/窒素量（NPC/N）を 150 にするための脂肪乳剤の液量 X に最も近い値はどれか。**1 つ**選べ。

（提案した処方）

50%ブドウ糖含有基本液	400 mL
20%脂肪乳剤（2 kcal/mL）	X mL
10%総合アミノ酸製剤	600 mL（総窒素量 9 g）
総合ビタミン剤	5 mL
微量元素製剤	2 mL

1　60
2　150
3　275
4　550
5　690

問 344　53 歳男性。入院中に発症した頻脈性不整脈に対して以下の処方が開始となった。

（処方）

シベンゾリンコハク酸塩錠 100 mg　1 回 1 錠（1 日 3 錠）

1 日 3 回　朝昼夕食後　4 日分

4 日後、朝服用して 2 時間後に血中濃度を測定すると 920 ng/mL となっていた（目標域：800 ng/mL 以下）。なお、患者の eGFR は 30 mL/min/1.73 m^2 であり、肝機能値は正常範囲内であった。この症例に対する薬剤師が医師に行う提案として、適切でないのはどれか。**1つ**選べ。

1　徐脈や血圧低下の可能性があるため、脈拍数と血圧をモニターする。

2　低血糖が現れる可能性があるため、血糖値を測定する。

3　PQ の延長や QRS 幅の増大などの心電図異常が認められる可能性があるため、心電図をモニターする。

4　腎機能に応じた投与量の減量をする。

5　過量投与への対応として 5 %ブドウ糖液とフロセミドを使用する。

問 345　65 歳男性。農作業で薬剤散布中、突然、呼吸困難を訴えたため救急搬送された。家族が持参した褐色ビンの貼付ラベルから商品名は読み取れず、一般名はイソフルロフェートと記載があった。なお、構造式は以下のとおりであることが判明した。

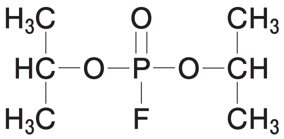

薬物中毒が疑われる本症例に対して用いる最も適切な解毒薬はどれか。**1 つ**選べ。

1　薬用炭
2　チオ硫酸ナトリウム
3　プラリドキシムヨウ化物
4　フルマゼニル
5　ジメルカプロール
