# Supplementary material for: ChatGPT (GPT-4) passed the Japanese National License Examination for Pharmacists in 2022, answering all items including those with diagrams: a descriptive study
Source: J Educ Eval Health Prof. 2024 Feb 28;21:4. doi: 10.3352/jeehp.2024.21.4 (PMC10948916; doi:10.3352/jeehp.2024.21.4)
Supplement: Supplementary file 4 — Supplement 3. The correct answers of the 107th Japanese National License Examination for Pharmacists (JNLEP) (in Japanese). [file jeehp-21-04-suppl3.pdf]

資料2

第107回薬剤師国家試験合格基準及び正答について

令 和 ４ 年 ３ 月 ２４ 日
厚生労働省医薬・生活衛生局

1. 第107回薬剤師国家試験の合格基準

以下のすべての基準を満たした者を合格とする。

・全問題の得点が434点以上

・必須問題について、全問題への配点の70%以上で、かつ、構成する各科目の得点がそれぞれ配点の30%以上

・禁忌肢問題選択数は2問以下

（注） 配点は1問2点（690点満点）

2. 試験問題正答

必須問題 【問No.１～９０】

1日目①

問No	科目	正答
1	物理	4
2	物理	1
3	物理	3
4	物理	3
5	物理	1
6	化学	5
7	化学	2
8	化学	2
9	化学	2
10	化学	4
11	生物	1
12	生物	1
13	生物	3
14	生物	4
15	生物	4
16	衛生	5
17	衛生	4
18	衛生	2
19	衛生	2
20	衛生	3
21	衛生	2
22	衛生	3
23	衛生	2
24	衛生	3
25	衛生	4
26	薬理	2
27	薬理	2
28	薬理	3
29	薬理	1
30	薬理	1

問No	科目	正答
31	薬理	5
32	薬理	4
33	薬理	4
34	薬理	1
35	薬理	3
36	薬理	5
37	薬理	5
38	薬理	5
39	薬理	3
40	薬理	2
41	薬剤	4
42	薬剤	2
43	薬剤	5
44	薬剤	3
45	薬剤	3
46	薬剤	4
47	薬剤	5
48	薬剤	3
49	薬剤	2
50	薬剤	4
51	薬剤	5
52	薬剤	3
53	薬剤	5
54	薬剤	4
55	薬剤	2
56	病態	2
57	病態	5
58	病態	4
59	病態	1
60	病態	1

問No	科目	正答
61	病態	1
62	病態	3
63	病態	4
64	病態	4
65	病態	3
66	病態	5
67	病態	5
68	病態	3
69	病態	4
70	病態	3
71	法規	3
72	法規	4
73	法規	3
74	法規	1
75	法規	3
76	法規	5
77	法規	5
78	法規	4
79	法規	2
80	法規	4
81	実務	4
82	実務	1
83	実務	5
84	実務	2
85	実務	1
86	実務	3
87	実務	3
88	実務	2
89	実務	5
90	実務	4

一般問題（薬学理論問題）
【問No.９１～１９５】

1日目②

問No	科目	正答	
91	物理	1	5
92	物理	2	3
93	物理	1	5
94	物理	2	
95	物理	2	4
96	物理	5	
97	物理	2	5
98	物理	4	5
99	物理	2	
100	物理	4	5
101	化学	1	
102	化学	3	4
103	化学	2	
104	化学	5	
105	化学	3	
106	化学	3	5
107	化学	2	
108	化学	2	
109	化学	4	
110	生物	1	4
111	生物	3	
112	生物	1	4
113	生物	4	5
114	生物	2	3
115	生物	1	4
116	生物	1	3
117	生物	1	5
118	生物	3	4
119	生物	2	5
120	衛生	3	5

問No	科目	正答	
121	衛生	1	4
122	衛生	3	
123	衛生	4	
124	衛生	3	
125	衛生	3	
126	衛生	2	
127	衛生	1	5
128	衛生	1	3
129	衛生	2	5
130	衛生	2	4
131	衛生	3	4
132	衛生	1	2
133	化学	1	
134	法規	2	
135	衛生	3	
136	衛生	1	
137	衛生	3	4
138	衛生	1	3
139	衛生	4	5
140	衛生	1	3
141	衛生	1	3
142	法規	1	4
143	法規	4	
144	法規	2	3
145	法規	1	
146	法規	1	4
147	法規	4	5
148	法規	2	3
149	法規	1	2
150	法規	4	

一般問題（薬学実践問題）【問No.196～345】

1日目③

問No	科目	正答	
151	薬理	2	3
152	薬理	2	3
153	薬理	4	5
154	薬理	2	4
155	薬理	2	4
156	病態	3	4
157	薬理	1	5
158	薬理	3	4
159	病態	4	5
160	薬理	1	3
161	薬理	2	5
162	薬理	2	4
163	薬理	1	2
164	薬理	1	5
165	病態	1	5
166	薬理	3	5
167	薬理	3	5
168	薬理	2	5
169	薬剤	4	
170	薬剤	3	
171	薬剤	2	4
172	薬剤	3	
173	薬剤	2	5
174	薬剤	1	4
175	薬剤	2	
176	薬剤	3	4
177	薬剤	2	4
178	薬剤	2	
179	薬剤	1	5
180	薬剤	1	3
181	薬剤	1	5
182	薬剤	1	4
183	薬剤	2	5
184	病態	3	5
185	病態	1	4
186	病態	2	3
187	病態	3	4
188	病態	3	4
189	病態	4	5
190	病態	2	4
191	病態	2	
192	病態	2	3
193	病態	1	5
194	病態	3	4
195	病態	5	

2日目①

問No	科目	正答	
196	実務	2	3
197	物理	1	
198	実務	3	
199	物理	1	3
200	実務	2	5
201	物理	5	
202	実務	4	
203	物理	3	4
204	実務	2	5
205	実務	1	5
206	物理	1	5
207	化学	4	
208	実務	2	
209	化学	3	
210	実務	1	3
211	化学	2	5
212	実務	2	4
213	化学	5	
214	化学	2	
215	実務	2	
216	生物	1	
217	実務	3	
218	実務	4	
219	生物	1	2
220	実務	5	
221	生物	2	
222	生物	1	
223	実務	3	
224	生物	2	3
225	実務	5	
226	実務	1	
227	衛生	5	
228	実務	3	4
229	衛生	3	4
230	実務	4	
231	衛生	3	
232	実務	1	
233	衛生	4	
234	衛生	1	2
235	実務	2	
236	実務	3	
237	衛生	1	4
238	衛生	2	
239	実務	2	
240	実務	3	
241	衛生	4	
242	実務	5	
243	衛生	1	4
244	実務	3	
245	衛生	5	

2日目②

問No	科目	正答	
246	薬理	1	5
247	実務	1	4
248	薬理	3	
249	実務	2	4
250	実務	4	
251	薬理	1	2
252	実務	1	5
253	薬理	5	
254	実務	1	2
255	薬理	1	5
256	薬理	3	5
257	実務	2	
258	薬理	2	4
259	実務	1	2
260	実務	1	4
261	薬理	4	
262	実務	2	3
263	薬理	5	
264	実務	2	3
265	薬剤	4	
266	薬剤	5	
267	実務	1	
268	実務	4	
269	薬剤	2	
270	薬剤	5	
271	実務	3	4
272	薬剤	3	4
273	薬理	1	4
274	実務	1	5
275	実務	5	
276	実務	4	
277	薬剤	3	4
278	実務	1	3
279	薬剤	1	2
280	薬剤	3	
281	実務	5	
282	実務	5	
283	薬剤	3	4
284	実務	4	5
285	薬剤	1	2

2日目③

問No	科目	正答	
286	病態	5	
287	実務	1	
288	実務	1	3
289	病態	3	5
290	病態	1	5
291	実務	2	4
292	病態	3	4
293	実務	2	3
294	病態	1	2
295	実務	4	
296	実務	2	4
297	病態	2	4
298	病態	1	5
299	実務	2	4
300	病態	3	5
301	実務	2	3
302	病態	1	4
303	実務	2	4
304	病態	4	
305	実務	2	4
306	実務	3	
307	法規	1	4
308	実務	2	
309	法規	1	5
310	法規	3	4
311	実務	4	5
312	実務	1	
313	法規	1	3
314	実務	1	4
315	法規	1	5
316	実務	4	5
317	法規	5	
318	実務	3	4
319	法規	1	4
320	実務	1	
321	法規	1	3
322	実務	2	3
323	法規	1	3
324	実務	2	5
325	法規	3	5
326	実務	1	2
327	実務	1	5
328	実務	解なし	
329	実務	1	
330	実務	3	4

問No	科目	正答	
331	実務	2	
332	実務	1	4
333	実務	2	3
334	実務	4	
335	実務	4	
336	実務	2	
337	実務	2	4
338	実務	2	4
339	実務	1	4
340	実務	2	3
341	実務	5	
342	実務	4	
343	実務	3	
344	実務	5	
345	実務	3	
